# Synergy between Enterobacteriaceae and diet mediates competition between dominant Bacteroidales in the human gut

**DOI:** 10.1038/s41564-026-02500-6

**Published:** 2026-10-02

**Authors:** Caroline Tawk, Youssef El Mouali, Kun D. Huang, Sebastiaan Valkiers, Vitor Heidrich, Achim Gronow, Lisa Osbelt, Johanna Rapp, Hannes Link, Nicola Segata, Athanasios Typas, Till Strowig

**Affiliations:** 1 Department of Microbial Immune Regulation, Helmholtz Centre for Infection Research, Braunschweig, Germany; 2 Department of CIBIO, University of Trento, Trento, Italy; 3 German Center for Infection Research (DZIF), partner site Hannover-Braunschweig, Braunschweig, Germany; 4 Interfaculty Institute for Microbiology and Infection Medicine Tübingen, University of Tübingen, Tübingen, Germany; 5 Cluster of Excellence ‘Controlling Microbes to Fight Infections’, University of Tübingen, Tübingen, Germany; 6 M3 Research Center, University of Tübingen, Tübingen, Germany; 7 IEO, Istituto Europeo di Oncologia IRCSS, Milan, Italy; 8 European Molecular Biology Laboratory, Molecular Systems Biology Unit, Heidelberg, Germany; 9 Cluster of Excellence RESIST (EXC 2155), Hannover Medical School, Hannover, Germany; 10 Center for Individualized Infection Medicine (CiiM), a joint venture of Hannover Medical School and HZI, Hannover, Germany; 11 Present Address: IrsiCaixa, Badalona, Barcelona Spain

**Keywords:** Microbial ecology, Microbiome

## Abstract

Healthy human gut microbiota is dominated by Bacteroidaceae in industrialized populations and by Prevotellaceae in those with traditional lifestyles, but diet alone is insufficient to explain this dominance of different Bacteroidota. Here we screened 94 dietary components in synthetic gut communities and find that these two bacterial families compete for overlapping nutritional niches. *Segatella copri*, a key member of Prevotellaceae-rich communities, outcompeted Bacteroidaceae when preferred polysaccharides such as arabinan were available, but this advantage depended on community composition, particularly the presence of Enterobacteriaceae. Metatranscriptomics and targeted metabolomics further revealed that Enterobacteriaceae synergized with dietary components to promote *Segatella* dominance in human and mouse gut communities. These interactions were validated by using multiple *E. coli* and *Segatella* isolates. Analyses of global metagenomes also showed a positive correlation between *Segatella*-rich non-industrialized microbiomes and Enterobacteriaceae abundance. Our results establish an ecosystem-level dynamic interplay between dietary components and key gut commensals that favour the dominance of Prevotellaceae.

## Abstract (web summary)

Multiomic experiments using defined and native gut communities reveal that complex dietary glycans mediate the dominance of Prevotellaceae in non-industrialized gut metagenomes through signalling-mediated cross-feeding with Enterobacteriaceae.

## Main

Profiling of the human gut microbiota at a global scale revealed diverse compositional structures of the healthy microbiota^1,2^. These distinct signatures have been described as enterotypes or bacterial guilds and were attributed to specific lifestyles and dietary patterns^3–5^. However, the factors that determine common microbiota signatures or enterotypes remain experimentally uncharacterized. Establishing clear causal links between intrinsic or extrinsic factors and microbiota signatures has proven challenging due to the inherent complexity and variability of the microbiome^6^. The use of synthetic defined gut communities has emerged as a key approach for investigating microbial ecological interactions, such as cross-feeding and antagonism, and community functional capacities, such as responses to drug perturbations, colonization resistance and community resilience^7–10^.

Two widely observed gut microbiota signatures, shaped by the abundant Bacteroidota phylum, notably diverge at the bacterial family level. Some individuals are dominated by Bacteroidaceae species, while others are dominated by Prevotellaceae species^11^. Bacteroidaceae are prevalent mainly in industrialized populations, correlate with diets rich in animal fat and processed foods, and have been the subject of extensive investigation^12,13^. Prevotellaceae, namely, members of the *Segatella copri* species complex (formerly *Prevotella copri*), are prevalent in non-industrialized populations, correlate with plant-rich diets and rural lifestyles, and are much less understood^14^. Interestingly, dietary patterns alone do not fully account for the distinct microbiota signatures associated with these bacterial families, suggesting that there are additional unidentified factors, such as competition for similar dietary glycans, that influence their establishment^12,15–18^. Specifically, bacteria belonging to these two families encode for a large repertoire of glycan utilization loci named polysaccharide utilization loci (PULs) and numerous Carbohydrate-Active enZYmes (CAZymes). *Segatella* spp. encode an average of 25 PULs and 40 CAZymes predicted to mainly degrade plant-derived polysaccharides^19,20^, whereas species in the Bacteroidaceae family often encode hundreds of PULs and CAZymes specialized in the degradation of plant- and animal-derived polysaccharides^21^. Thus, complex carbohydrates may influence competition and dominance among *Segatella* spp. and Bacteroidaceae. However, the precise interactions between these remain unclear.

Here we designed synthetic gut communities to resolve the specific interactions between *Segatella copri* and Bacteroidaceae species within a complex microbial network. We demonstrate that Prevotellaceae and Bacteroidaceae compete within the community, where *S. copri* outcompete Bacteroidaceae depending on preferred dietary glycans and the presence of other commensals in the community. Specifically, Enterobacteriaceae species largely promote *S. copri* within the community. In our metagenomic analysis of human cohorts, we found that Enterobacteriaceae are both prevalent and diverse in *Segatella*-rich, non-industrialized human microbiomes and foods. Together, our study reveals that the interplay between dietary fibre and Enterobacteriaceae shapes the competitive dynamics of dominant Bacteroidota in the gut.

## Results

### Dietary components induce major shifts in a gut community

**Fig. 1:**
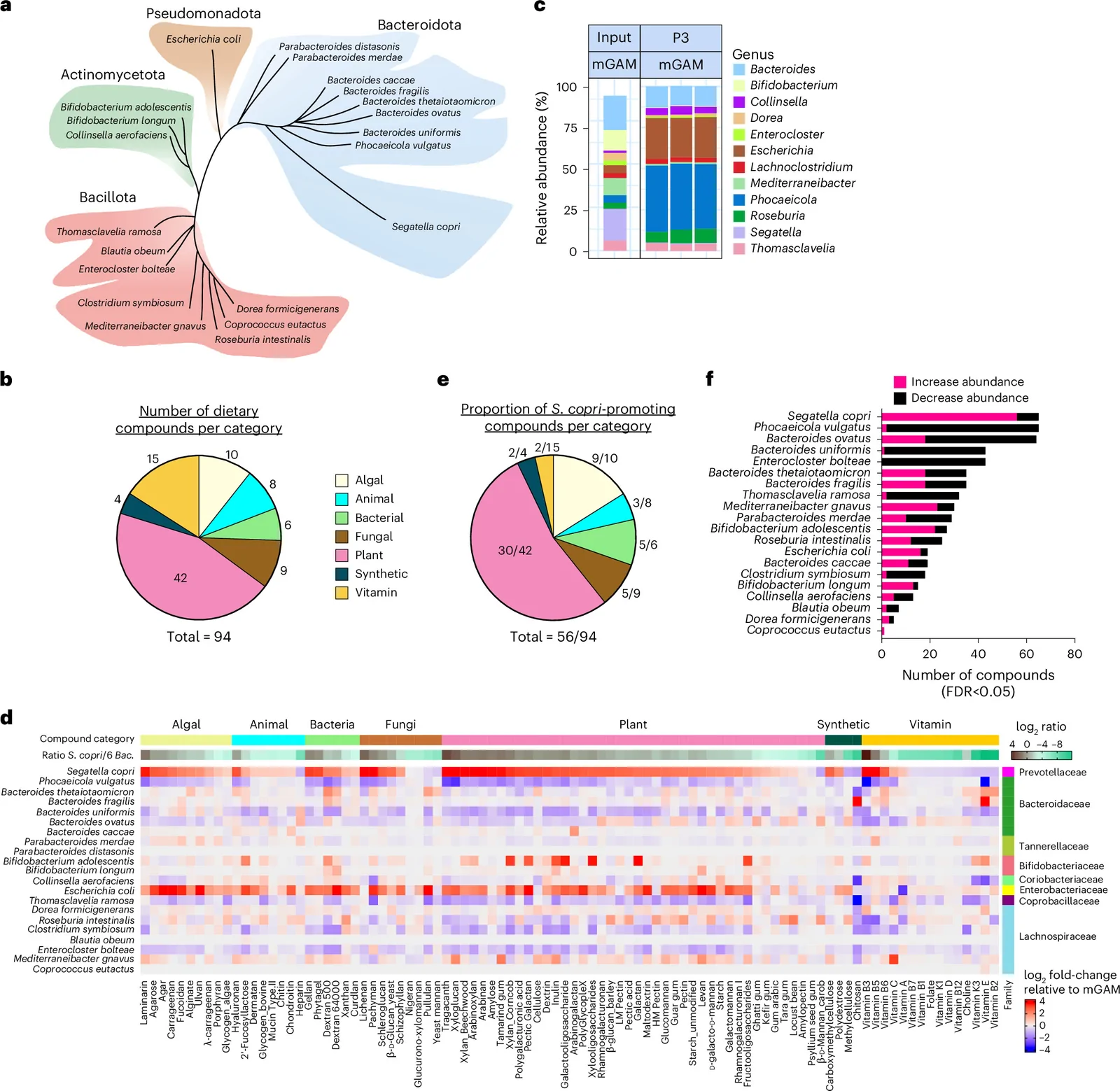
Food-derived complex carbohydrates and vitamins promote *Segatella copri* in a gut community model. **a**, Phylogenetic representation of the 21 commensal human isolates comprising the defined gut community. **b**, Pie chart representing the number of dietary compounds tested in each category. **c**, Taxonomic barplots showing the relative abundance of the top 12 community genera at passage 3 and the input in mGAM. Data are from 16S rRNA amplicon sequencing with 3 biological replicates shown. **d**, Heat map showing the log_2_ fold-change of the relative abundance of each member within the community in response to each dietary compound compared to the base rich media (mGAM). The log ratio of the relative abundance of *S. copri* over the sum relative abundance of the 6 Bacteroidaceae strains has been calculated for each condition (ratio *S. copri*/6Bac.). Data represent the average from 3 replicates. **e**, Pie chart representing the proportion of dietary compounds from each category that significantly increase the relative abundance of *S. copri* in the community (FDR < 0.05; Kruskal–Wallis with false discovery rate correction). **f**, Total number of dietary compounds that decrease or increase the relative abundance of each commensal member within the community (*n* = 3; FDR < 0.05; Kruskal–Wallis with false discovery rate correction). Source data

**Extended Data Fig. 1:**
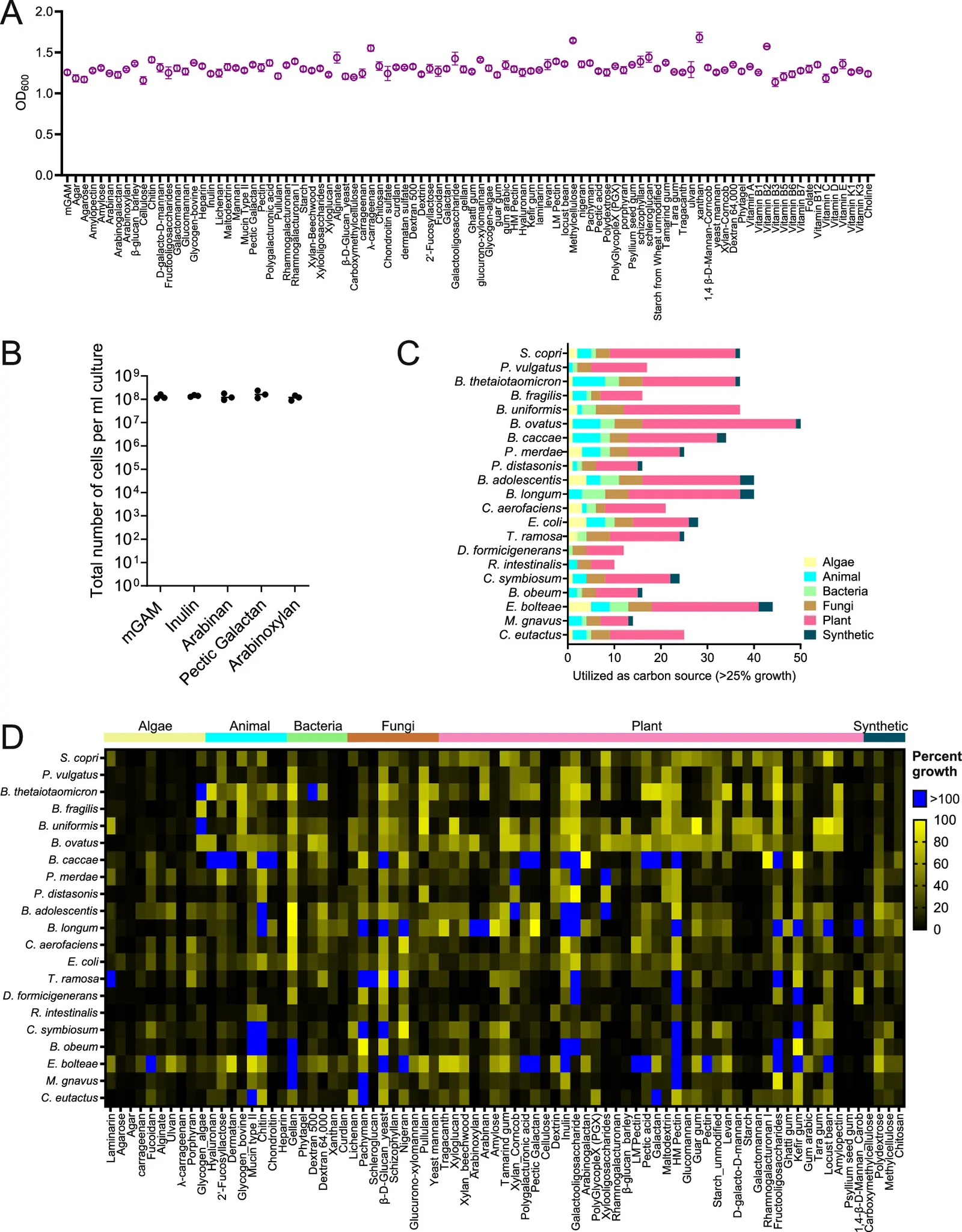
Growth of individual commensal community members on food-derived polysaccharide. (**A**) Final OD600 of the defined community at P3 after 24h growth in the different supplemented mGAM conditions. Dash represents median value with SD, n = 3 biological replicates. (**B**) Total number of cells determined by flow cytometry in the defined community after 24h growth in mGAM alone or supplemented with a complex polysaccharide. n=3 biological replicates. (**C**) Total number of complex polysaccharides from each category that are utilized as a carbon source for growth (>25 % growth as a fraction of growth in mGAM) by individual commensal member. (**D**) Heatmap representing the percentage of growth of individual community members on food-derived polysaccharides as a sole carbon source compared to growth in mGAM (See Methods; Supplementary Table 8). Purple color (>100%) represents better growth on a specific complex polysaccharide compared to growth in mGAM. Data represents the average from n=3. Source data

To investigate the specific factors that drive the predominance of Prevotellaceae within a bacterial gut community, we designed a synthetic commensal community comprising 21 human gut isolates representing the 4 major phyla of the human microbiota (Fig. 1a and Supplementary Table 1; see Methods). We tested a total of 94 dietary components, including 79 complex dietary glycans from diverse sources for their effect on gut community structure and composition (Fig. 1b). We included 15 essential vitamins into our analysis due to their potential influence on bacterial competition in the gut community^22^. The community was grown in modified Gifu anaerobic medium (mGAM), a rich medium where the main defined carbohydrate sources are starch (0.5%) and glucose (0.05%). The glycans were added to mGAM at a concentration range of 0.04–0.2%, and the vitamins were added at their specific physiological supplementation ranges in dietary interventions (Supplementary Table 2; see Methods). The communities were first assembled in mGAM, and the relative abundance of each member was quantified using 16S rRNA amplicon sequencing at the initial timepoint (Input) and at passage 3 (P3) once the community had stabilized. Bacteroidaceae (represented by 5 species from the *Bacteroides* genus: *Bacteroides thetaiotaomicron, Bacteroides fragilis, Bacteroides uniformis, Bacteroides ovatus* and *Bacteroides caccae*, and one species from the *Phocaeicola* genus: *Phocaeicola vulgatus*) predominated over Prevotellaceae (represented by *Segatella copri*) within the community in mGAM alone (Fig. 1c). Supplementation with a single glycan or vitamin was sufficient to induce large changes in the community composition (Fig. 1d). Most components did not significantly affect the final optical density or total cell number of the community after 24 h of growth (Extended Data Fig. 1a,b). A majority of glycans (56/94), particularly those of plant, algal and bacterial origin, led to a significant expansion of *S. copri* (Fig. 1d,e). Interestingly, for most of these compounds, *S. copri* expanded at the expense of Bacteroidaceae strains, shifting the balance towards a higher relative abundance of *S. copri* compared with the Bacteroidaceae strains (ratio *S. copri*/6Bac., Fig. 1d). The addition of vitamin B3 and B5 was also sufficient to increase *S. copri* relative abundance in the gut community (Fig. 1d,e). Notably, the bacterial species whose relative abundance was significantly affected by most compounds belonged to the Bacteroidales order: *S. copri* and the Bacteroidaceae species, as well as *Enterocloster boltae* (Fig. 1f). *E. coli* relative abundance exhibited considerable variability in response to multiple compounds within the community, although only a minority of these changes reached statistical significance (*n* = 17; 38% of the compounds; Fig. 1d,f). Interestingly, the relative abundance of *S. copri* was increased by the largest number of compounds (*n* = 56) (Fig. 1f). In contrast, the relative abundance of *P. vulgatus* was decreased by the largest number of compounds (*n* = 63), followed by *B. ovatus* (*n* = 46) and *B. uniformis* (*n* = 42) (Fig. 1f).

To determine which species in the community are capable of utilizing the different compounds, we tested each of the 21 individual community members for their capacity to grow on each of the 79 dietary glycans as a sole carbon source (*n* = 1,659 bacteria–glycan pairs; Extended Data Fig. 1c,d and Supplementary Table 2). We identified that 5 Bacteroidales, *Bifidobacteria* and *E. boltae* grew on most compounds (Extended Data Fig. 1c). Interestingly, despite *B. ovatus* growing on the largest number of compounds and other Bacteroidaceae utilizing a similar number of compounds to *S. copri*, most glycans promoted *S. copri* growth in the community at the expense of the Bacteroidaceae (Fig. 1d (ratio *S. copri*/6Bac.) and Extended Data Fig. 1c). In addition, the capacity of the different species to utilize the glycans did not always predict the direction of Prevotellaceae/Bacteroidaceae ratios within the community. For instance, pectic galactan is utilized by *B. caccae*, *B. thetaiotaomicron*, *B. ovatus*, *B. uniformis*, *P. vulgatus* and *S. copri* with calculated growth rates 0.62 h^−1^, 0.5 h^−1^, 0.5 h^−1^, 0.39 h^−1^, 0.18 h^−1^ and 0.13 h^−1^, from highest to lowest, respectively. When pectic galactan is supplemented to the community, it leads to an increase in *S. copri* within the community and not the Bacteroidaceae species (Fig. 1d and Extended Data Fig. 1d). Further, *B. thetaiotaomicron*, *P. vulgatus* and *S. copri* all utilize arabinan (at descending growth rates of 0.5 h^−1^, 0.42 h^−1^ and 0.12 h^−1^), and when added, it leads to an expansion of *S. copri* and a depletion of the Bacteroidaceae members (Fig. 1d and Extended Data Fig. 1d). These results show that complex carbohydrates and vitamins strongly affect gut community structure, particularly the Prevotellaceae/Bacteroidaceae ratio, but that assessing the utilization capacity of dietary components by individual commensal species in monoculture does not reliably predict the overall community dynamics.

### Community members influence *Segatella copri* and Bacteroidaceae competition

**Fig. 2:**
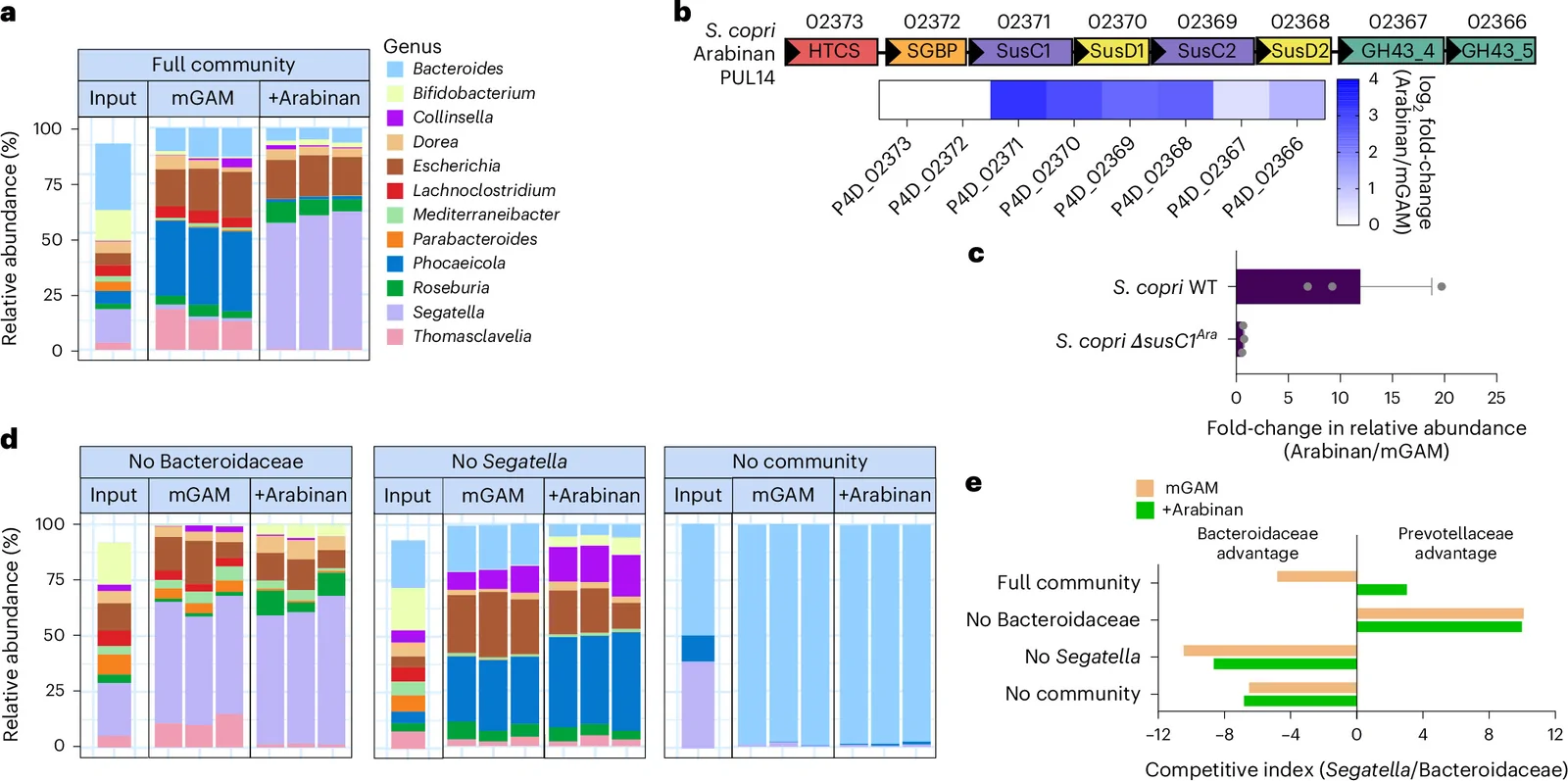
*S. copri* competitive advantage over Bacteroidaceae species in arabinan depends on other commensal members of the gut community. **a**, Taxonomic barplots depicting the relative abundance of the top 12 community genera at passage 3 and the input in mGAM and mGAM with arabinan (5 mg ml^−1^). Data are from 16S rRNA amplicon sequencing, with 3 biological replicates shown. **b**, Top: schematic representation of the genes in the *S. copri* arabinan utilization PUL14. Bottom: heat map displaying the log_2_ fold-change in gene expression of *S. copri* PUL14 genes at 20 h within the community in the presence of arabinan compared to mGAM alone. **c**, log_2_ fold-change of the relative abundance of *S. copri* WT and Δ*susC1*^*Ara*^ mutant within the community in the presence of arabinan compared to mGAM alone. Bars represent the average ± s.d., *P < 0.05 (unpaired two-sided Student’s *t*-test, *n* = 3 biological replicates). **d**, Taxonomic barplots depicting the relative abundance of the top 12 community genera at passage 3 and the input, in the presence of arabinan or mGAM alone. Data are from 16S rRNA amplicon sequencing, with 3 biological replicates shown. ‘No Bacteroidaceae’ communities exclude 6 Bacteroidaceae species, ‘No *Segatella*’ communities exclude *Segatella copri*, ‘No community’ includes only 6 Bacteroidaceae species and *Segatella copri*. **e**, Calculated competitive index between Prevotellaceae and Bacteroidaceae in community assemblies from **a** and **d** (see Methods). In **a** and **d**, data shown are from representative experiments. Source data

**Extended Data Fig. 2:**
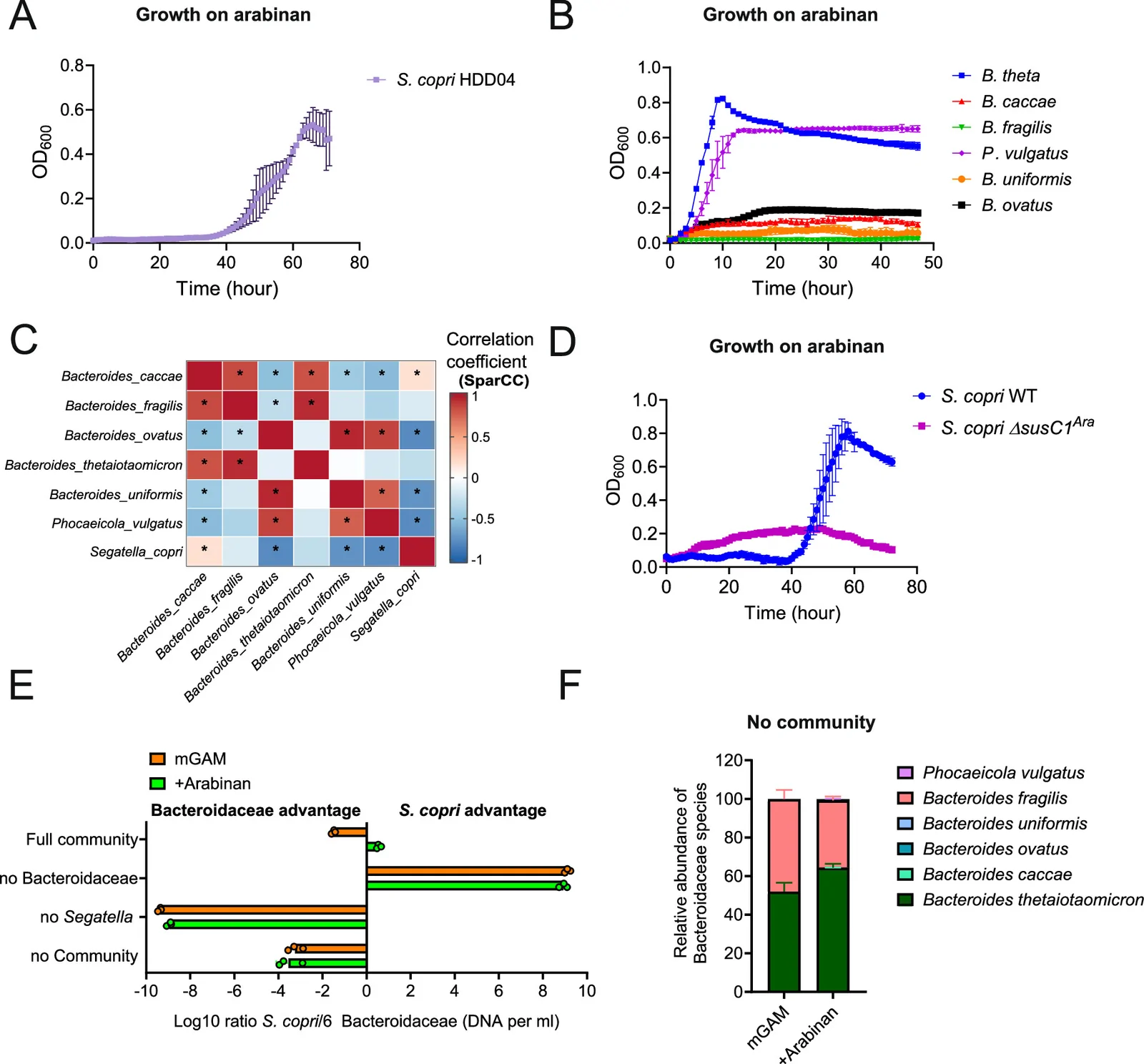
Competition of *Segatella sp*. with Bacteroidaceae within the defined gut community. (**A**) Growth of *S. copri* HDD04 in minimal media with arabinan as a sole carbon source. Data represents the average ±SD, n=3 biological replicates. (**B**) Growth of the six Bacteroidaceae individual strains in minimal media with arabinan as a sole carbon source. Data represents the average ±SD, n=3 biological replicates. (**C**) Correlations between community members across community settings in Fig. 2 were estimated using SparCC^76^ with 20 inference iterations. P-values were assessed by empirical bootstrap/permutation test using the SparCC utilities MakeBootstraps.py and PseudoPvals.py with 20 simulations (https://github.com/dlegor/SparCC). (**D**) Growth of *S. copri* WT and Δ*susC1*Ara mutant in minimal media with arabinan as a sole carbon source. Data represents the average ±SD, n=3 biological replicates. (**E**) Log ratio of the absolute DNA concentration of *S. copri* over the sum of the six Bacteroidaceae quantified with qPCR in the different communities in Fig. 2 (see Methods), n=3. (**F**) Relative abundance determined by quantitative PCR of the different Bacteroidaceae species when competing with S. copri in the absence of other community members (“No community”; Fig. 2d). Data represents the average ±SD, n=3 biological replicates. Source data

We next investigated whether *S. copri* and the Bacteroidaceae species compete within the community. We chose arabinan supplementation as a model, as arabinan promotes *S. copri* over the Bacteroidaceae species, and the PUL specific for arabinan utilization is known for *S. copri* and *B. thetaiotaomicron*, and is predicted for *P. vulgatus*^19,23^. Moreover, *B. thetaiotaomicron*, *P. vulgatus* and *S. copri* grow efficiently on arabinan (Extended Data Fig. 2a,b). Compared to mGAM, supplementation of arabinan led to a ~35-fold increase in *S. copri* relative abundance (1.7% vs 59.6%) and >6-fold decrease in overall Bacteroidaceae (47% vs 7.3%) (Fig. 2a). Thus, *S. copri* has a clear advantage within the community in the presence of arabinan and shows a negative correlation with most members of the Bacteroidaceae (Fig. 2a,e and Extended Data Fig. 2c). Metatranscriptome analysis revealed that the SusC/D-like gene pairs in the *S. copri* arabinan-specific PUL14 are upregulated up to ~16-fold compared to mGAM alone, suggesting active arabinan metabolization by *S. copri* within the community (Fig. 2b and Supplementary Table 3). Of note, genes in the *B. thetaiotaomicron* arabinan PUL were also upregulated (Supplementary Table 3). Deletion of SusC1 in the arabinan PUL14 renders *S. copri* unable to utilize arabinan^19^ (Extended Data Fig. 2d). Unlike *S. copri* WT, the *S. copri* Δ*susC1* mutant strain was unable to expand within the community in the presence of arabinan (Fig. 2c). These results show that *S. copri* gains a competitive advantage within the community by directly utilizing arabinan.

Next, to determine whether *S. copri* and Bacteroidaceae compete within the community, we assembled the communities by omitting either *S. copri* or all six Bacteroidaceae species. We found that in the absence of Bacteroidaceae, *S. copri* occupies the full Bacteroidales niche both in mGAM and with arabinan (Fig. 2d,e and Extended Data Fig. 2e). Conversely, Bacteroidaceae occupied the full Bacteroidales niche in both media in the absence of *S. copri* (Fig. 2d,e and Extended Data Fig. 2e). This indicates that *S. copri* and Bacteroidaceae species directly compete within the community and have distinct advantages in the presence of different complex carbohydrates (for example, arabinan vs starch). We hypothesized that *S. copri* could scavenge arabinan more efficiently than *P. vulgatus* and *B. thetaiotaomicron*, thereby gaining a competitive advantage within the community. To test this, we directly competed *S. copri* with the six Bacteroidaceae species in the absence of other community members. Surprisingly, *S. copri* was fully outcompeted by the Bacteroidaceae species, primarily *B. thetaiotaomicron* and *B. fragilis*, in the presence of arabinan when other commensals were absent (Fig. 2d,e and Extended Data Fig. 2e,f). Together, these results indicate that while direct arabinan utilization is necessary for *S. copri* expansion within the community, it is not sufficient; interactions with community members are required for *S. copri* to dominate in the presence of a preferred complex glycan.

### *S. copri* expansion within the community depends on *E. coli*

**Fig. 3:**
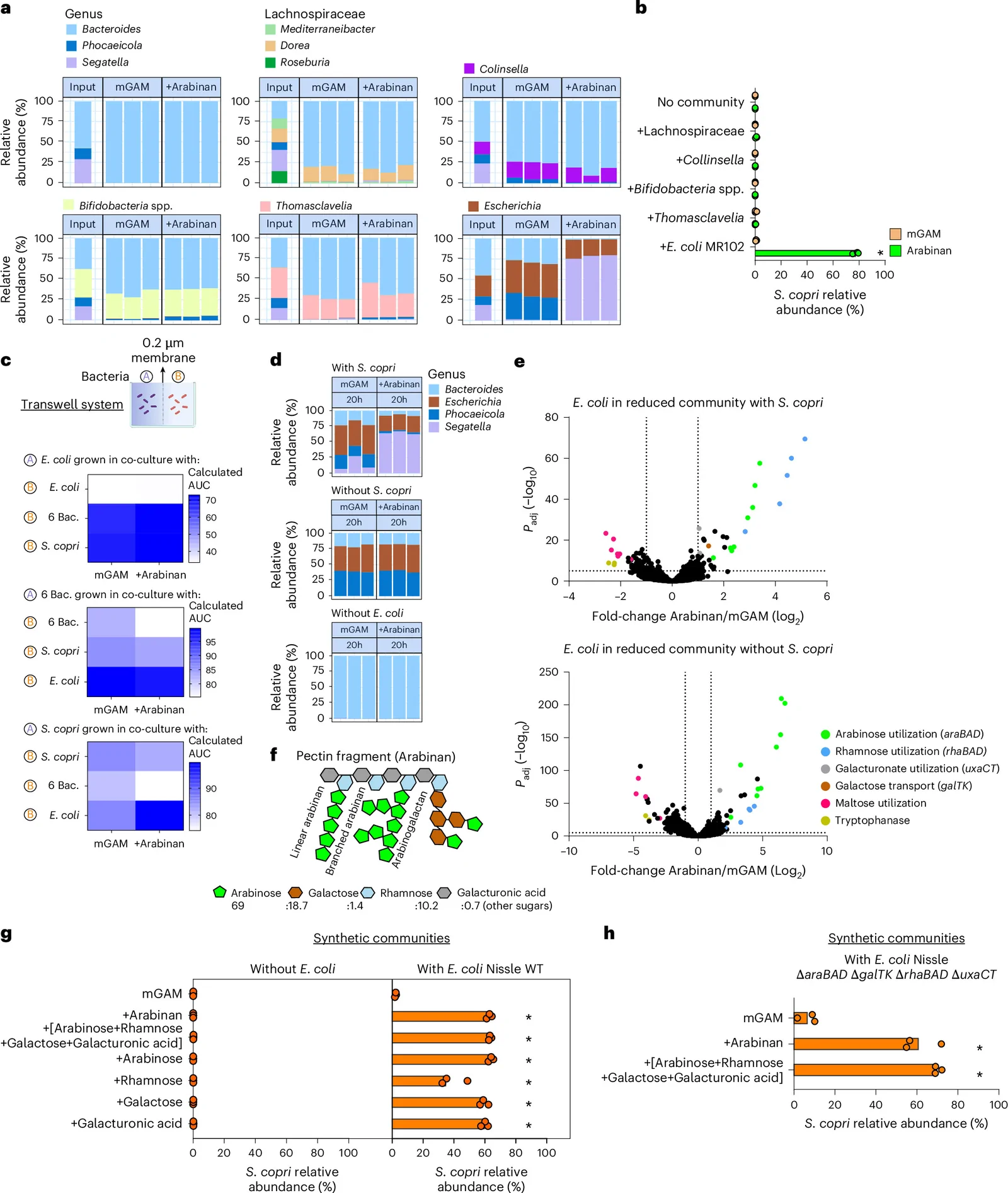
*E. coli* provides a competitive advantage to *S. copri* over Bacteroidaceae. **a**, Taxonomic barplots at the genus level showing the relative abundance of the community members in the input and at passage 3 with arabinan or mGAM alone. Data are from 16S rRNA amplicon sequencing, with 3 biological replicates shown. **b**, *S. copri* relative abundance in the competition with Bacteroidaceae, with the addition of different species in the presence or absence of arabinan. *P < 0.0001 from two-sided *t*-test with correction for multiple comparisons, *n* = 3 biological replicates. **c**, Schematic of transwell co-culture platform (top). Area under the curve (AUC) calculated from growth curves in transwell co-cultures (bottom). Each species was co-cultured with itself as a monoculture control. The data are representative of 3 independent experiments (Extended Data Fig. 3a,b). **d**, Relative abundance of the genera in the different community assemblies: ‘with *S. copri*’, ‘without *S. copri*’ and ’without *E. coli*’. Data are from 16S rRNA amplicon sequencing, with 3 biological replicates shown. **e**, Volcano plots showing gene expression changes in *E. coli* MR102 within communities ‘with *S. copri*’ or ‘without *S. copri*’ in the presence of arabinan compared to mGAM control at 8 h. Gene clusters required for the utilization of the respective sugars are indicated. Wald test was used to determine the *P* value and the Benjamini–Hochberg to correct *P* values for multiple testing (*P*_adj_). **f**, Schematic representation of a pectin fragment depicting the monosaccharides constituting arabinan polysaccharides. **g**, Competitions between *S. copri* and 6 Bacteroidaceae in the presence or absence of *E. coli* Nissle in mGAM supplemented with the different sugars. The relative abundance of *S. copri* in the communities is shown (*n* = 3 biological replicates). *P < 0.0001 by two-way analysis of variance (ANOVA) with Dunnett’s correction for multiple comparisons. **h**, Competitions between *S. copri* and 6 Bacteroidaceae in the presence *E. coli* Nissle Δ*araBAD* Δ*rhaBAD* Δ*galTK* Δ*uxaCT*, that is unable to metabolize the arabinan-derived simple sugars, in mGAM supplemented with the different sugars. The relative abundance of *S. copri* in the communities is shown (*n* = 3 biological replicates). *P < 0.0001 by one-way ANOVA with Holm–Sidak correction for multiple comparisons. Source data

To identify the community member(s) that provide a competitive advantage to *S. copri*, we employed an add-in strategy where we competed *S. copri* with the six Bacteroidaceae species and added commensal members that displayed >0.1% relative abundance in the community in the presence of arabinan (Fig. 2a). Species from the remaining three phyla were tested; Bacillota (three Lachnospiraceae spp. or *Thomasclavelia*) or Actinomycetota (two *Bifidobacteria* spp. or *Collinsella*) did not alter the competitive advantage of Bacteroidaceae over *Segatella* in all conditions (Fig. 3a,b). Strikingly, addition of *E. coli* was sufficient to shift the competitive advantage in favour of *Segatella* in the presence of arabinan (Fig. 3a,b). This shows that *E. coli* alone is sufficient to provide *S. copri* with a competitive advantage over Bacteroidaceae species in the presence of arabinan.

**Extended Data Fig. 3:**
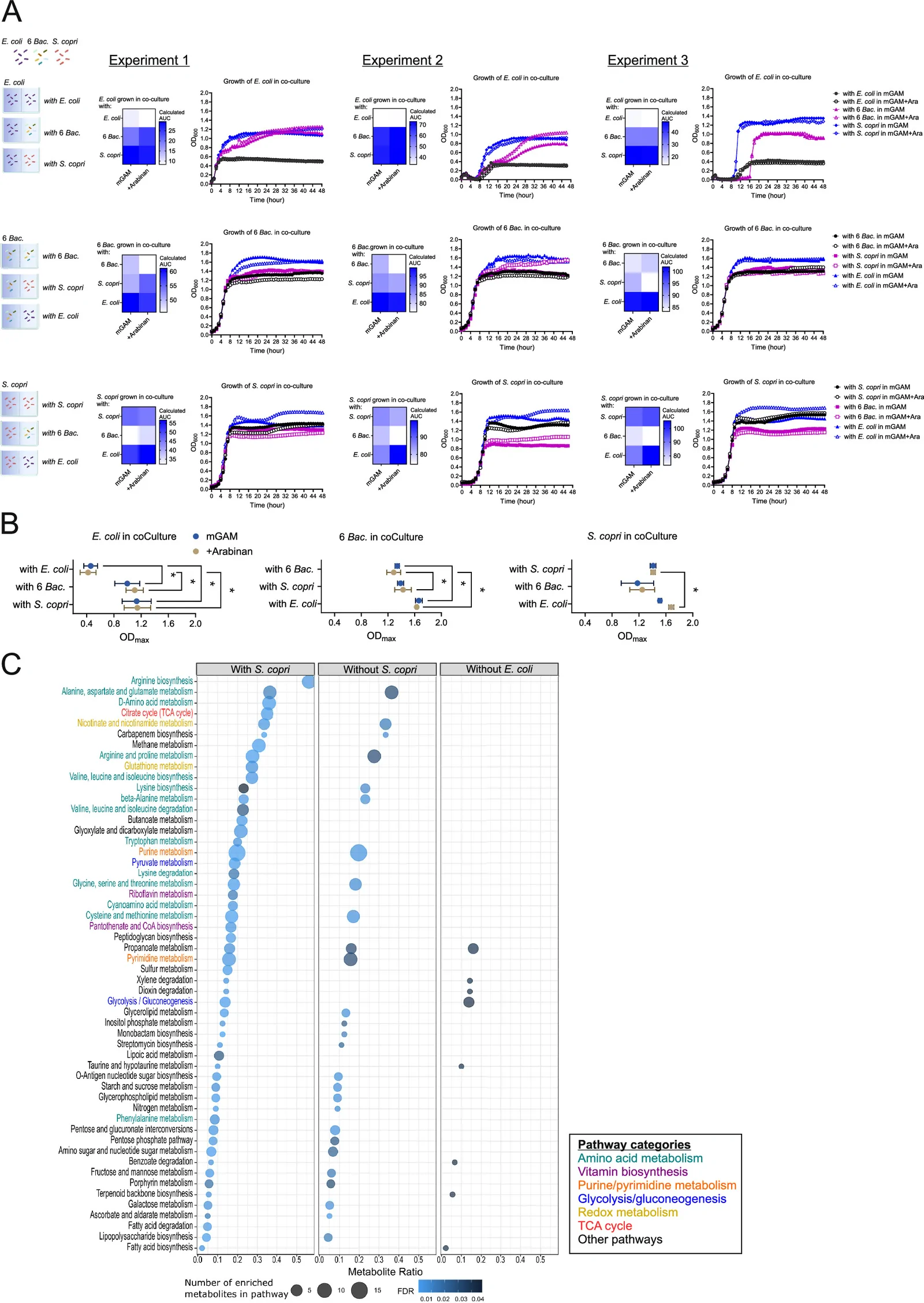
Cross-feeding between the Bacteroidales and *E. coli* in transwell co-cultures. (**A**) Schematic of the transwell co-cultures setup (left). Growth curves of *E. coli*, 6 Bacteroidaceae species (6 Bac.), and *S. copri* in the different co-culture conditions and the corresponding calculated Area Under the Curve (calculated AUC). The data is from three independent co-culture experiments. Schematic of experimental design was generated in BioRender. (**B**) ODmax from the growth curves in A for each *E. coli*, 6 Bacteroidaceae species (6 Bac.), and *S. copri*. n=3, data are presented as mean values +/- SD. *p-value<0.05 by two-way ANOVA with Benjamini and Hochberg correction for multiple comparisons. (**C**) Dot plot displaying enrichment of metabolic pathways upon arabinan supplementation compared to the mGAM control for each community assembly shown in Fig. 3d. "Metabolite Ratio" denotes the proportion of significantly changed metabolites relative to the total metabolites in the pathway. Metabolic pathway enrichments with FDR <0.05 were considered significant. n = 3. Source data

To determine the influence of *E. coli* on the growth of the Bacteroidales and vice versa, we performed transwell co-culture assays allowing nutrient exchange with different combinations of *S. copri*, *E. coli* and the six Bacteroidaceae compared to monocultures (Fig. 3c and Extended Data Fig. 3a,b). Interestingly, we observed that *E. coli* displayed improved growth when co-cultured with *S. copri* or the six Bacteroidaceae species in mGAM and in the presence of arabinan (Fig. 3c and Extended Data Fig. 3a,b). No inhibition of Bacteroidaceae growth was observed under any condition (Extended Data Fig. 7a). *S. copri* growth was modestly inhibited by co-culture with Bacteroidaceae, and remarkably, *S. copri* growth was boosted when co-cultured with *E. coli* in the presence of arabinan compared to mGAM alone (Fig. 3c and Extended Data Fig. 3a,b). These results are consistent with *E. coli* and arabinan synergizing to promote *S. copri* within the community.

### Cross-feeding and sugar-dependent signalling occur between Bacteroidales and *E. coli*

To uncover potential nutrient exchange mechanisms, we investigated the metabolic and transcriptomic landscape of representative community states. First, we performed targeted metabolomics on the different community assemblies with or without arabinan supplementation. Arabinan altered the greatest number of metabolic pathways when supplemented to the community containing both *S. copri* and *E. coli* (‘with *S. copri*’) (Fig. 3d, Extended Data Fig. 3c, and Supplementary Table 4 and Information).

**Extended Data Fig. 4:**
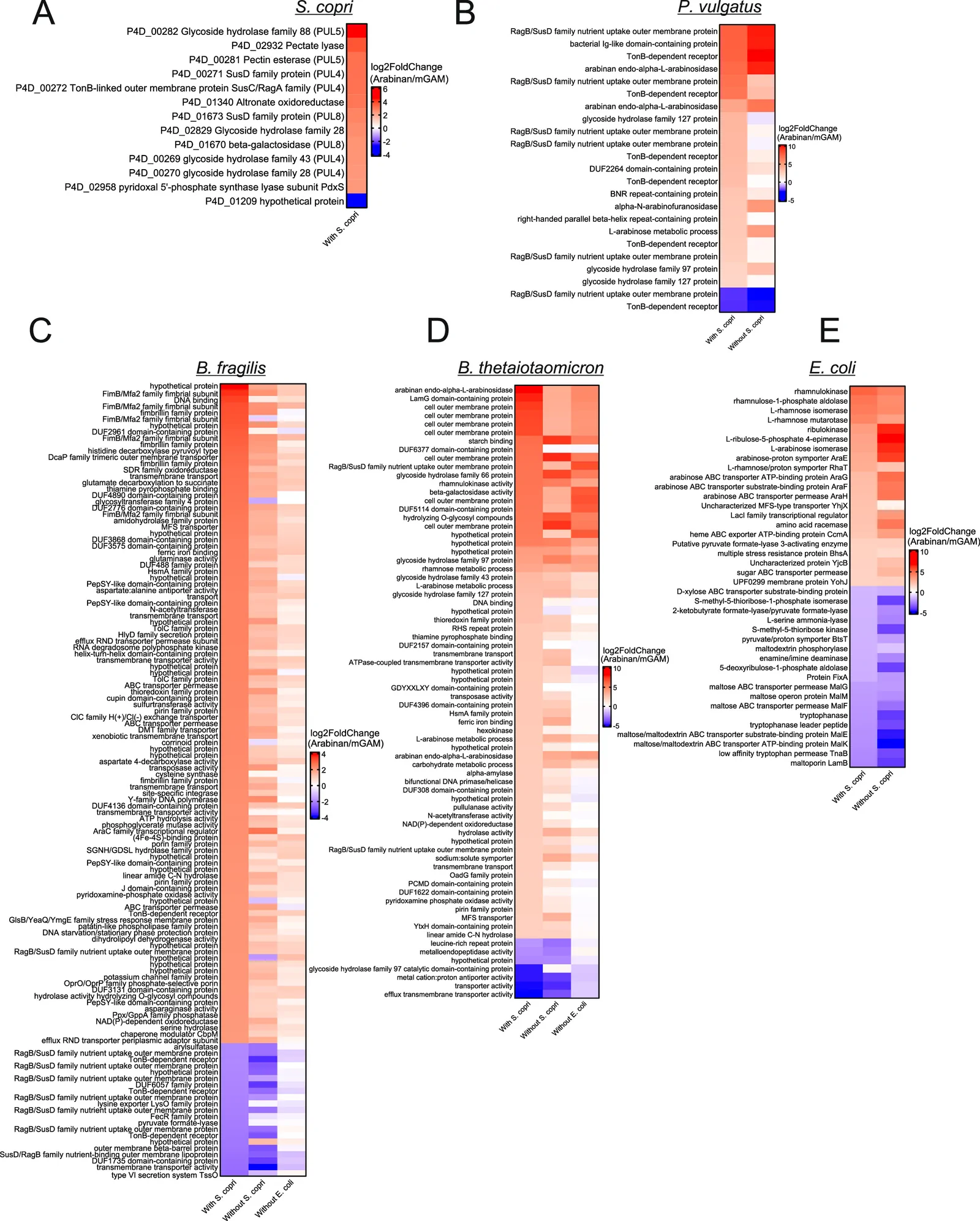
*E. coli* cross-feeding on simple sugars from arabinan degradation by the Bacteroidales. (**A**-**E**) Heatmap representing the Log2 fold-change in gene expression of *S. copri* (**A**), *P. vulgatus* (**B**), *B. fragilis* (**C**), *B. thetaiotaomicron* (**D**), and *E. coli* (**E**) within the community assemblies “with *S. copri*”, “without *S. copri*” and “without *E. coli*” in the presence of arabinan, compared to the mGAM control at 8 h. Displayed changes correspond to Log_2_ fold-change values ≤ -2 or ≥ 2 and an adjusted p-value (Padj) <0.01, n=3. Wald test was used to determine the P-value and the Benjamini-Hochberg to correct p-values for multiple testing (P-adj).

Next, we performed metatranscriptomics to analyse the gene expression of individual members. We analysed gene expression at 8 h for *S. copri* and the three most abundant Bacteroidaceae species in the community assemblies: *B. thetaiotaomicron*, *B. fragilis* and *P. vulgatus*. Arabinan induced nutrient-acquisition genes in *S. copri*, including PUL14, transporters and glycosyl hydrolases, consistent with a shift from starch to arabinan utilization. The three dominant Bacteroidaceae showed similar responses, with differential regulation of PULs, transporters and glycosyl hydrolases; *B. fragilis* also upregulated fimbrial genes (Extended Data Fig. 4, and Supplementary Table 5 and Information). Interestingly, gene expression analysis of *E. coli* in communities with and without *S. copri* at 8 h showed an upregulation of genes involved in arabinose, rhamnose, galacturonate and galactose utilization, and a downregulation of genes involved in maltose utilization and tryptophanase in the presence of arabinan (Fig. 3e,f, Extended Data Fig. 4e and Supplementary Table 5). This suggests that *E. coli* is engaged in cross-feeding on monosaccharides deriving from polysaccharide degradation in mGAM (maltose from starch), and switches upon arabinan supplementation (arabinose, rhamnose, galacturonate and galactose) (Fig. 3e,f). These results indicate that *E. coli* cross-feeds on monosaccharides derived from arabinan degradation by *S. copri* in the ‘with *S. copri*’ community, and by *B. thetaiotaomicron* and *P. vulgatus* ‘without *S. copri*’.

**Extended Data Fig. 5:**
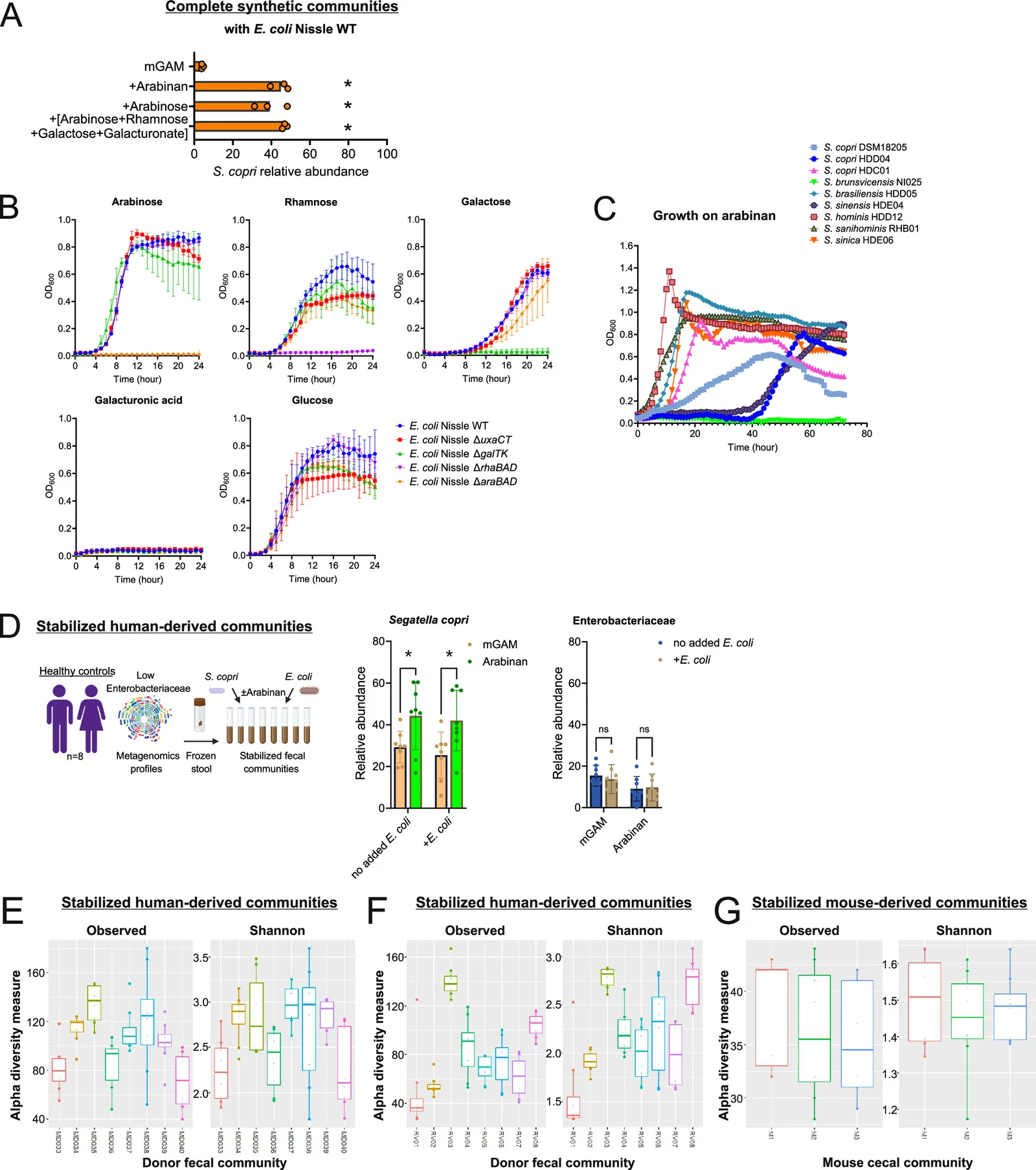
Growth of *E. coli* strains on different carbohydrates. (**A**) *S. copri* relative abundance within the full synthetic community with *E. coli* Nissle WT in mGAM supplemented with the different sugars (n=3 biological replicates), *p-value<0.0001 by one-way ANOVA with Holm-Sidak correction for multiple comparisons. (**B**) Growth of *E. coli* strains in minimal M9 media with a single carbon source provided. Data represents the average ±SD, n=3 biological replicates. (**C**) Growth of individual *Segatella* strains in minimal media with arabinan as a sole carbon source. Data represents the average of three replicates. (**D**) Eight donors with undetectable Enterobacteriaceae in the corresponding metagenomic datasets were selected. Fecal communities were stabilized from frozen stool and coalesced with *S. copri* HDD04 with or without *E. coli* MR102 in the presence of arabinan or mGAM alone. Data is from 16S rRNA amplicon sequencing. *p-value<0.05 from unpaired two-sided t-test with correction for multiple comparisons. n=8, Bars represent the average ±SD. (**E**-**G**) Calculated alpha diversity (observed richness and Shannon index) of stabilized gut communities derived from (**E**) human fresh stool n=8, (**F**) human frozen stool n=8, and (**G**) mouse cecum n=3. Box plot are presented as: centre line indicates the median, the box bounds represent the first and third quartiles (25th and 75th percentiles), and the whiskers extend to the most extreme values within 1.5 × the interquartile range; points beyond the whiskers are plotted as outliers. Cohort schematics have been created with Biorender. Source data

We next supplemented the simple sugars derived from arabinan degradation directly into the *S. copri*/Bacteroidaceae competition, with or without *E. coli* Nissle. Interestingly, adding individual sugars or their mix (arabinose, rhamnose, galactose and galacturonic acid) promoted *S. copri* expansion when *E. coli* was present, but not when it was absent, as well as in the full community (Fig. 3g and Extended Data Fig. 5a). Finally, to determine whether *E. coli* needs to use these sugars to promote *S. copri* expansion, we generated a quadruple *E. coli* Nissle mutant (Δ*araBAD* Δ*rhaBAD* Δ*galTK* Δ*uxaCT*) that cannot utilize the arabinan-derived sugars (Fig. 3e,f and Extended Data Fig. 7b–e). Interestingly, this mutant still promoted *S. copri* in the presence of arabinan or the sugars, even though it is unable to metabolize them (Fig. 3h and Extended Data Fig. 5b). Overall, these findings suggest that the presence of simple sugars, rather than their utilization by *E. coli*, is sufficient to promote *S. copri* within the community in an *E. coli*-dependent manner.

### *Segatella* spp. competitive advantage depends on dietary components and Enterobacteriaceae

**Fig. 4:**
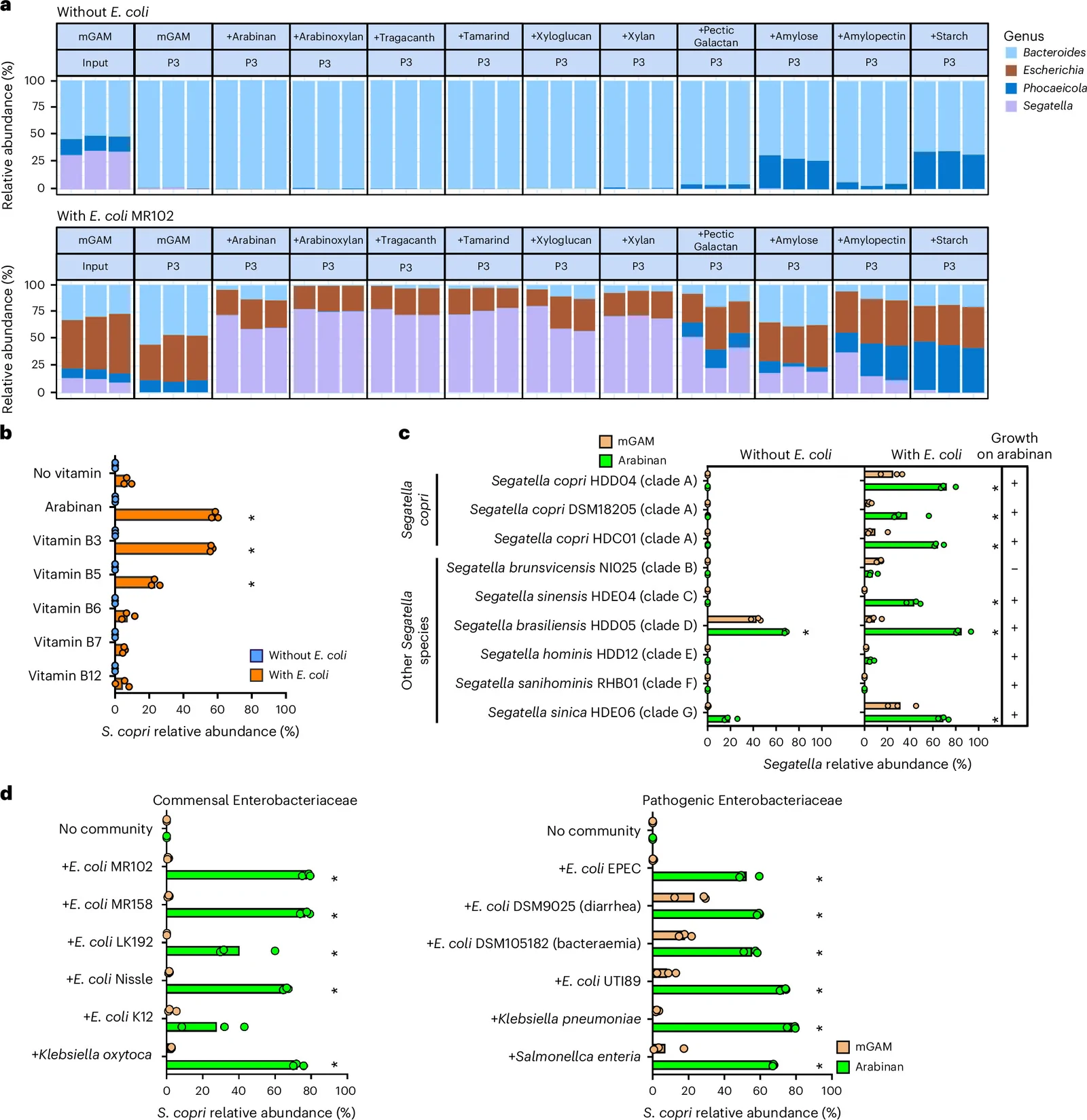
Enterobacteriaceae provide a competitive advantage to *S. copri* in the presence of various complex polysaccharides. **a**, Taxonomic barplots at the genus level showing the relative abundance of the community members in mGAM or with a supplemented polysaccharide. Data are from 16S rRNA amplicon sequencing, with 3 biological replicates shown. **b**, Competitions between *S. copri* and 6 Bacteroidaceae in the presence or absence of *E. coli* in mGAM supplemented with the different vitamins (Supplementary Table 2). The relative abundance of *S. copri* in the communities is shown (*n* = 3 biological replicates). *P < 0.05 by two-way ANOVA with Dunnett’s correction for multiple comparisons. **c**, Relative abundance of *Segatella* spp. in the competitions with Bacteroidaceae with or without *E. coli* MR102 in the presence or absence of arabinan. Growth on arabinan as a sole carbon source was tested for every *Segatella* strain in monocultures as shown in the right panel (Extended Data Fig. 5c). *n* = 3 biological replicates. **d**, *S. copri* relative abundance in the competitions with Bacteroidaceae, with the addition of different Enterobacteriaceae species in the presence or absence of arabinan. **c**,**d**, *P < 0.05 from unpaired two-sided *t*-test with correction for multiple comparisons, *n* = 3 biological replicates. Source data

We next evaluated whether complex glycans and vitamins other than arabinan could give *S. copri* an *E. coli*-dependent competitive advantage. All eight additional plant-derived polysaccharides tested promoted *S. copri* over Bacteroidaceae in an *E. coli*-dependent manner, including the starch derivatives amylose and amylopectin, but not starch (Fig. 4a). Interestingly, vitamin B3 and vitamin B5, but not other B vitamins, also promoted *S. copri* in an *E. coli*-dependent manner (Fig. 4b). This shows that *E. coli* synergizes with a variety of complex carbohydrates and vitamins to provide *S. copri* with a competitive advantage over Bacteroidaceae.

We next investigated whether the fibre-dependent interaction with *E. coli* is conserved beyond the *S. copri* HDD04 strain. *S. copri* strains DSM18205^T^ and HDC01 similarly gained an *E. coli*-dependent advantage in the presence of arabinan (Fig. 4c). We then extended our analysis to other *Segatella* species. We observe that conservation of this mechanism is somewhat associated with phylogenetic proximity^24^: the *S. copri* closely related species, *S. brasiliensis*, *S. sinensis* and *S. sinica* retain the *E. coli*-dependent competitive advantage in the presence of arabinan, with *S. brasiliensis* also outcompeting Bacteroidaceae in the absence of *E. coli* or arabinan, probably reflecting superior resource acquisition relative to other *Segatella* species (Fig. 4c and Extended Data Fig. 7f). In contrast, more distantly related species representatives (*S. brunsvicensis*, *S. hominis*, *S. sanihominis*) neither exhibit *E. coli*-dependent interactions nor outcompete Bacteroidaceae under any tested condition (Fig. 4c and Extended Data Fig. 5c). Collectively, these findings indicate that the fibre synergy with *E. coli* is conserved among *S. copri* strains and is present in other closely related *Segatella* species while absent in distantly related members, reflecting the extensive phylogenetic and ecological diversity within this genus.

Finally, we tested whether this observed positive interaction between *S. copri* and *E. coli* occurs with genetically diverse *E. coli* strains. We found that *S. copri* relative abundance increased in the presence of arabinan with all tested *E. coli* strains, including other commensal (MR158, LK192, Nissle)^25^ and laboratory strains (K-12), as well as pathogenic isolates (DSM9025, DSM105182, UTI89 and O127:H6) (Fig. 4d). Strikingly, other Enterobacteriaceae species, namely, a commensal isolate of *Klebsiella oxytoca*, a patient-derived *Klebsiella pneumoniae* strain and a *Salmonella enterica* strain also led to an increase in *S. copri* relative abundance in the presence of arabinan (Fig. 4d and Extended Data Fig. 7g). Altogether, these results show that there is a broadly conserved mechanism of positive interaction between *Segatella copri* and Enterobacteriaceae species in the presence of chemically diverse complex carbohydrates and vitamins.

### *E. coli* synergizes with arabinan to drive *S. copri* expansion in complex human and mouse gut communities

**Fig. 5:**
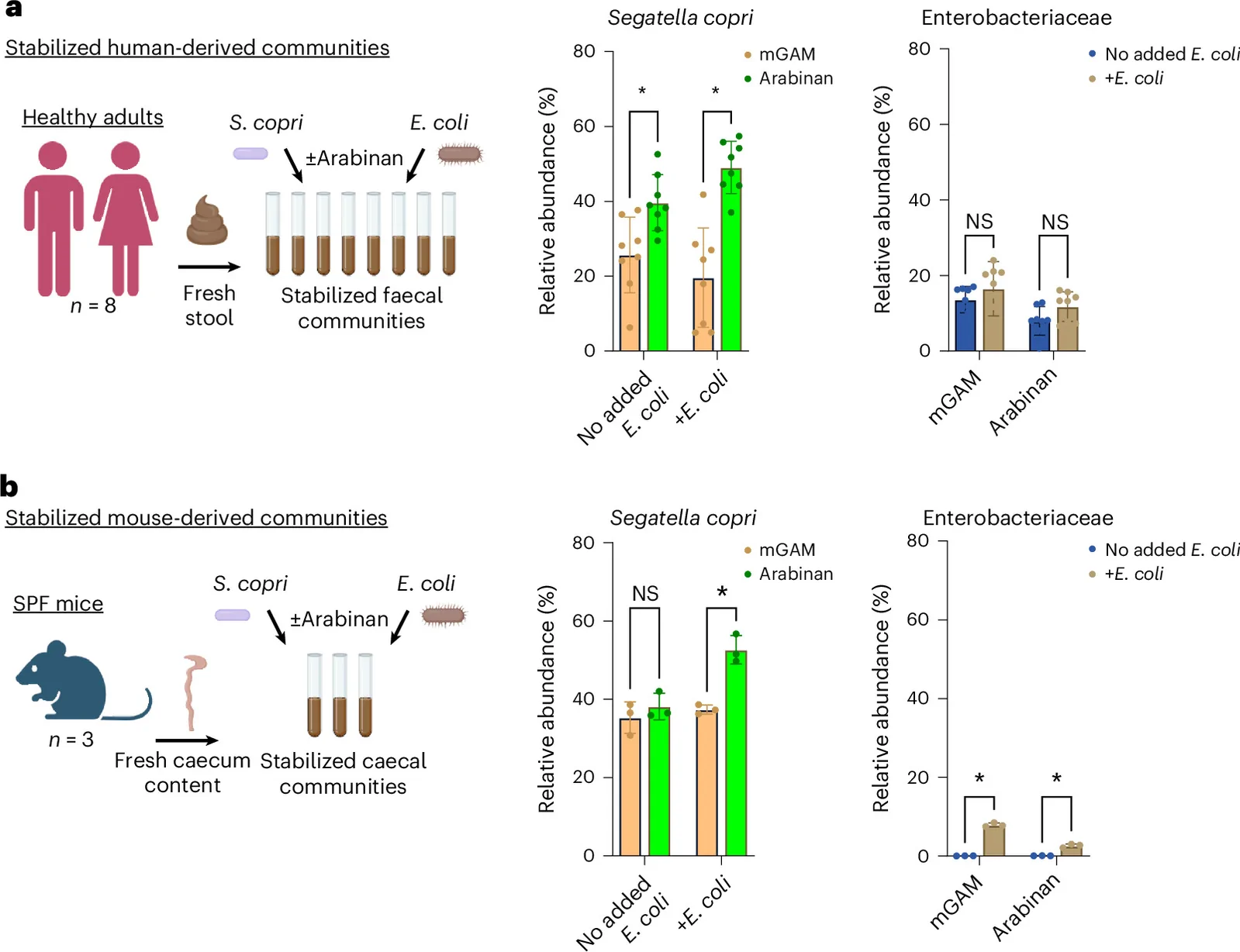
*E. coli* promotes *S. copri* within complex gut-derived communities in the presence of arabinan. **a**, Faecal communities (*n* = 8) were stabilized from fresh stool obtained from 8 healthy adult donors and coalesced with *S. copri* HDD04 with or without *E. coli* MR102 in the presence of arabinan or mGAM alone. **b**, Caecal communities were stabilized from fresh SPF mice caecal content (*n* = 3) and coalesced with *S. copri* HDD04 with or without *E. coli* MR102. **a**,**b**, Data are from 16S rRNA amplicon sequencing. The relative abundance of *S. copri* and Enterobacteriaceae in the gut-derived communities is shown. Bars represent the average ± s.d. *P < 0.05; NS, not significant; from unpaired two-sided *t*-test with correction for multiple comparisons. Cohort schematics were created with Biorender. Source data

Since synthetic communities are less complex than the native gut microbiota, we investigated whether the observed synergistic interaction also occurs in more complex, human-derived stabilized faecal communities (Fig. 5a)^26^. *S. copri* alone or in combination with *E. coli* was then introduced into the stabilized communities, with or without arabinan supplementation. Arabinan supplementation consistently promoted a marked expansion of *S. copri* in stabilized human faecal communities, recapitulating dynamics observed in synthetic communities (Fig. 5a and Extended Data Fig. 5d). However, Enterobacteriaceae levels in these communities were not significantly altered by exogenous *E. coli* addition, limiting the ability to attribute this effect specifically to *E. coli* (Fig. 5a and Extended Data Fig. 5d).

Finally, to overcome this limitation and further evaluate the specific *E. coli*–*S. copri* interaction in a background devoid of Enterobacteriaceae, we used caecal communities from in-house-bred specific pathogen-free (SPF) mice, which in this case naturally lack Enterobacteriaceae (Fig. 5b; ref. ^27^). Notably, in these communities, we observed a specific *E. coli*- and arabinan-dependent expansion of *S. copri* (Fig. 5b). Alpha-diversity analysis of the stabilized faecal and caecal communities revealed significantly greater community complexity compared with the synthetic communities (average observed richness 38–110; average Shannon index 1.5–3.0; Extended Data Fig. 5e–g). These experiments demonstrate that *S. copri* expansion is reproducible across complex human faecal and mouse gut communities and depends on both arabinan and *E. coli*.

### Enterobacteriaceae species are prevalent in non-industrialized microbiomes

**Fig. 6:**
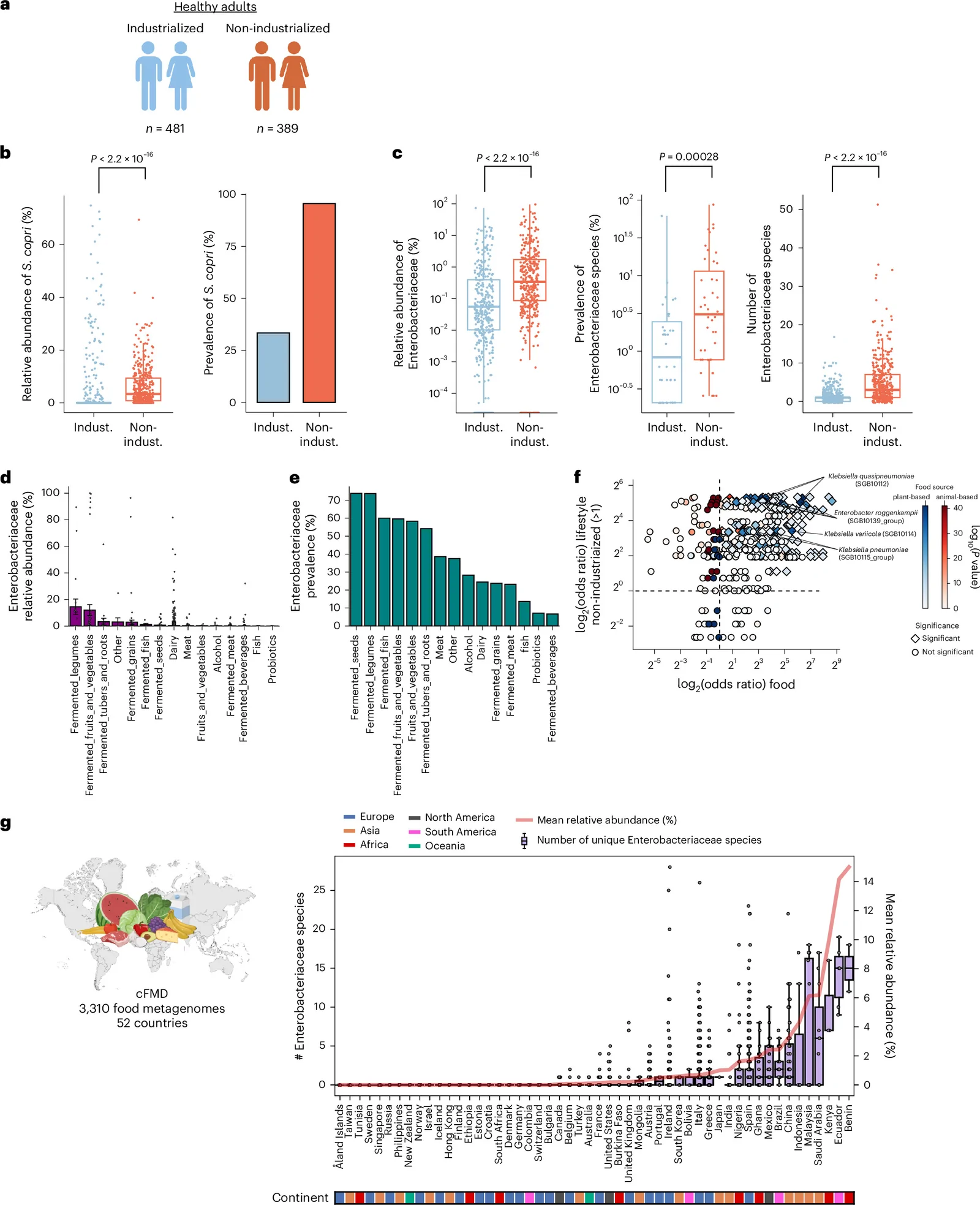
Enterobacteriaceae are prevalent in non-industrialized microbiomes. **a**, Schematic representing the healthy adult cohort with industrialized and non-industrialized lifestyles (Supplementary Table 6). **b**, Relative abundance and prevalence of *S. copri* in the gut microbiome of industrialized and non-industrialized healthy adults. *P* values were determined using two-tailed Wilcoxon rank-sum test. **c**, Relative abundance, prevalence and number of Enterobacteriaceae species in the gut microbiome of industrialized and non-industrialized healthy adults. *P* values were determined using two-tailed Wilcoxon rank-sum test. **d**,**e**, Relative abundance (**d**) and prevalence (**e**) of Enterobacteriaceae in the different categories of food microbiomes. In **d**, data are presented as mean ± s.e.m. **f**, Enrichment of SGBs assigned to Enterobacteriaceae in specific food categories, as well as lifestyle (industrialized vs non-industrialized), using two-sided Fisher’s exact test. *P* values were corrected for multiple hypothesis testing using the Benjamini–Hochberg procedure. **g**, Prevalence and relative abundance of Enterobacteriaceae species in food microbiomes from different countries, data from the cFMD database. Boxplots are presented as: middle line, median; lower and upper hinge, first and third quartiles; lower and upper whiskers, the smallest and largest value no further than 1.5× the interquartile range from the hinge; data beyond the whiskers are outlying points. In **g**, a total of *n* = 3,310 food metagenomes were analysed. Cohort schematics, maps and food items were created with Biorender.

**Extended Data Fig. 6:**
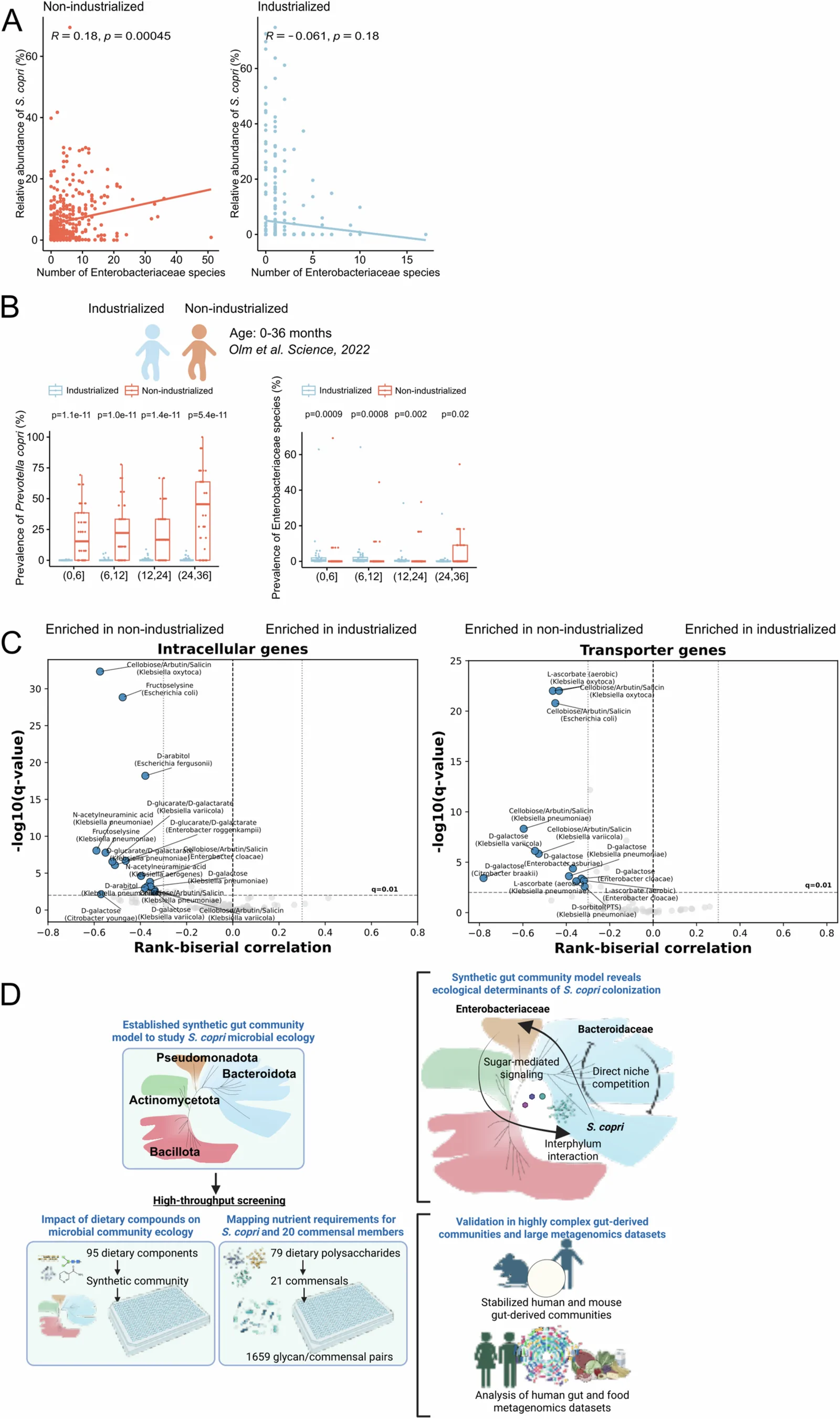
Non-industrialized microbiomes have a higher prevalence of Enterobacteriaceae. (**A**) Spearman correlation analysis of the relative abundance of *S. copri* with the number of Enterobacteriaceae species in non-industrialized and industrialized microbiomes. (**B**) Prevalence of *Prevotella copri* (left) and Enterobacteriaceae (right) species in the gut microbiome of non-industrialized and industrialized infants, data from 28. P-values were determined using a two-tailed Wilcoxon rank-sum test. Box plots were presented as: middle line, median; lower hinge, first quartile; upper hinge, third quartile; lower whisker, the smallest value at most 1.5× the interquartile range from the hinge; upper whisker, the largest value no further than 1.5× the interquartile range from the hinge; data beyond the whiskers are outlying points. n=1900 fecal samples from infants were analyzed. (**C**) Enrichment of Enterobacteriaceae carbohydrate metabolism pathways was compared between non-industrialized and industrialized samples using the two-tailed Mann-Whitney U test. p-values were corrected post hoc using the Benjamini-Hochberg. Schematics have been created with Biorender. (**D**) Schematic illustration of the experimental approaches and the main findings from this study.

**Extended Data Fig. 7:**
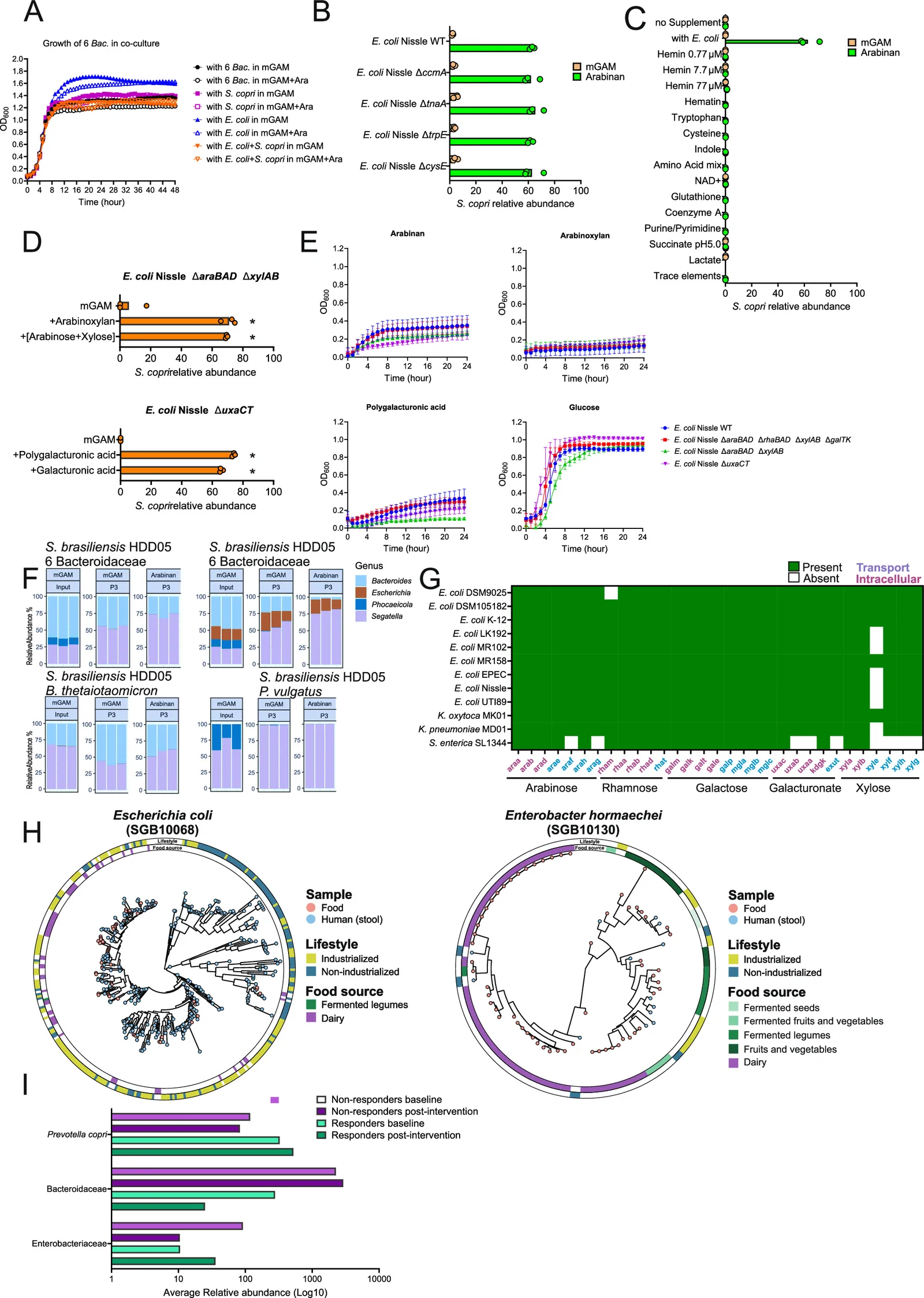
Sugar-signaling and cross-feeding between *S. copri* and *E. coli*. (**A**) Growth curves of the six Bacteroidaceae species grown in co-culture with: six Bacteroidaceae, with *S. copri*, with *E. coli*, and with *E. coli* + *S. copri* in the presence or absence of arabinan. (**B**) Competitions between *S. copri* and six Bacteroidaceae in the presence of different *E. coli* mutant strains. The relative abundance of *S. copri* in the communities is shown (n=3). (**C**) Supplementation of different components into the competition between *S. copri* and six Bacteroidaceae. The relative abundance of *S. copri* in the communities is shown (n=3). (**D**) Competitions between *S. copri* and six Bacteroidaceae in the presence of different *E. coli* Nissle strains, fibres and derived simple sugars. The relative abundance of *S. copri* in the communities is shown (n=3). *p-value<0.05 by two-way ANOVA with Dunnett’s correction for multiple comparisons. (**E**) Growth curves of the *E. coli* Nissle strains in minimal M9 media with the fibers arabinan, arabinoxylan, polygalacturonic acid, or glucose as control. (**F**) *S. brasiliensis* was competed either against the six Bacteroidaceae species in the presence or absence of *E. coli* and with or without arabinan, or in direct 1:1 competition with either *B. thetaiotaomicron* or *P. vulgatus*. Data is from 16S rRNA amplicon sequencing with three replicates shown. (**G**) Heatmap showing conservation (≥50% protein identity) of genes involved in simple sugar transport, sensing, and metabolism across the 12 Enterobacteriaceae species tested in this study. (**H**) Phylogenetic trees representing reconstructed strain-level phylogenies for *E. coli* (SGB10068) and *Enterobacter hormaechei* (SGB10130), which showed significant joint enrichment to a food category and stool samples of Non-industrialized lifestyle. (**I**) Average relative abundances of *Prevotella copri*, Bacteroidaceae, and Enterobacteriaceae are shown from the dietary intervention study in Kovatcheva-Datchary et al.^40^.

Next, we analysed publicly available gut metagenomes of ~1,000 healthy adult individuals with either industrialized or non-industrialized lifestyles to examine whether Enterobacteriaceae species are more prevalent in non-industrialized microbiomes, potentially supporting a predominance of Prevotellaceae (Fig. 6a and Supplementary Table 6). Consistent with previous reports, non-industrialized individuals harbour a higher prevalence and abundance of *S. copri* compared with industrialized individuals (Fig. 6b)^14^. Interestingly, non-industrialized individuals also harboured a significantly higher prevalence, diversity and abundance of Enterobacteriaceae species compared with industrialized individuals (Fig. 6c). Finally, we observed a positive correlation between *S. copri* relative abundance and the number of Enterobacteriaceae species in non-industrialized individuals but not in industrialized individuals (Spearman *R* = 0.18, *P* = 0.00045 and *R* = −0.061, *P* = 0.18; Extended Data Figs. 6a and 7i). The distinct Prevotellaceae-dominated and Bacteroidaceae-dominated microbiome signatures appear early in life (6–36 months of age), following an initial *Bifidobacteria*-dominated microbiome probably driven by exclusive milk consumption (0–6 months)^28^. As reported, non-industrialized infants exhibit a higher prevalence of *Segatella copri* from early life compared with industrialized infants (Extended Data Fig. 6b)^28^. Interestingly, we observed that Enterobacteriaceae prevalence differs among infants with different lifestyles. Despite a higher Enterobacteriaceae prevalence in the early life of industrialized infants, there is a significantly higher prevalence of Enterobacteriaceae in non-industrialized infants at 2–3 years of age (Extended Data Fig. 6b). This suggests that as *S. copri* becomes dominant, a prominent niche for Enterobacteriaceae also tends to form. The interaction between these species may become more relevant as dietary fibres preferred by *S. copri* become available.

Enterobacteriaceae are generally a minor inhabitant of the healthy human gut in terms of abundance, but they are major faecal contaminants that can survive and spread through the environment, including foods^29–34^. We questioned whether Enterobacteriaceae are more common in non-industrialized environments, especially in food products. To address this hypothesis, we used an extensive dataset of food metagenomes from the curatedFoodMetagenomicData (cFMD) database^35^. Strikingly, we found that plant-based foods, rich in fibre, have the highest relative abundance and prevalence of Enterobacteriaceae (Fig. 6d–f and Extended Data Fig. 7h). Moreover, Enterobacteriaceae members enriched in plant foods also exhibited concordant enrichment in stool microbiomes of individuals following non-industrialized lifestyles (Fig. 6f). We also observed that foods containing a significantly higher relative abundance and diversity of Enterobacteriaceae originate from countries characterized by predominantly non-industrialized lifestyles (Fig. 6g). In addition, we found that the gut microbiome of non-industrialized populations is significantly enriched in Enterobacteriaceae genes encoding simple sugar utilization functions, including those for sugar transport, sensing and metabolism, compared to industrialized microbiomes (Extended Data Fig. 6c). Altogether, these results show that Enterobacteriaceae are highly prevalent in non-industrialized microbiomes of healthy individuals and environmental settings such as foods.

## Discussion

The distinct prevalence of *Segatella* spp. in the human gut microbiomes has been documented; however, the underlying mechanisms remain largely unexplored experimentally^24^. Nevertheless, the predominance of *Segatella* spp. in specific global populations emphasizes the necessity for a more comprehensive understanding of these bacteria. The reasons underlying the preference for Bacteroidaceae over *Segatella* in industrialized microbiomes remain ambiguous. While a correlation with dietary patterns has been suggested^36,37^, other studies have failed to find a link between *Segatella* abundance and short-term dietary interventions or habitual diets, indicating that diet alone does not explain *Segatella* predominance^12,15,17,38^. Our synthetic gut community design, coupled with our previously established genetic tools and *Segatella* isolates, has illuminated the mechanisms governing *Segatella* predominance in the gut microbiota. Utilizing our experimental model, we demonstrate that *Segatella* preferentially assimilates a wide variety of complex dietary glycans, which act synergistically with Enterobacteriaceae to promote its expansion within a gut microbial community (Extended Data Fig. 6d).

A competitive relationship between *Segatella* and *Bacteroides* species has been suggested; however, it has not been experimentally validated^39^. In reported studies involving either 1:1 competitions or within commensal communities, *Bacteroides* spp. generally outcompete *S. copri*, or both species co-exist^40–43^. Whereas microbiota-accessible carbohydrates (MACs) facilitate *S. copri* colonization, they do not confer a competitive advantage for *S. copri* over *B. thetaiotaomicron* alone^42^. We observed that within the commensal community, *S. copri* and Bacteroidaceae compete. Specifically, Bacteroidaceae prevailed over *S. copri* in the presence of various complex glycans, but *S. copri* overcame this competitive disadvantage by leveraging support from other community members. The introduction of the community, or solely *E. coli* provided a single *Segatella* strain with a competitive advantage over multiple Bacteroidaceae species (including *Bacteroides* and *Phocaeicola*) in the presence of a preferred polysaccharide or vitamin. Although, nutrient-mediated mutualistic or antagonistic pairwise interbacterial relationships within the microbiome have been well documented^44–46^, this study provides an example of more complex interactions, in which both nutrient acquisition and bacterial cross-talk are required for *Segatella* to dominate the microbial community.

Our findings reveal that besides its specific preference for plant-derived fibres, *S. copri* also exhibited expansion in response to most fibres derived from microbial and algal sources. As previously noted, only a small portion of animal or host-derived glycans enhanced the abundance of *S. copri* in the community^20,42^. This suggests that *S. copri* possesses a broad preference for complex glycans from various sources despite having a significantly smaller number of glycan-processing enzymes when compared with *B. thetaiotaomicron* and *B. ovatus*^19,20,47^. This may imply a greater versatility of *S. copri*’s PULs and CAZymes or indicate the existence of additional unknown loci involved in diverse complex glycan utilization. This provides an important resource for future studies on *Segatella* carbohydrate utilization and its effects on colonization. In addition to dietary glycans, vitamin B3 and B5 promote *S. copri* expansion while decreasing Bacteroidaceae abundance within the community in an *E. coli*-dependent manner (Figs. 1 and 4). *S. copri* and various Bacteroidaceae species are predicted to synthesize most B vitamins, which are essential for their colonization^48–50^. It has been shown that dietary supplementation with these vitamins increases the relative abundance of *Prevotella* and decreases that of *Bacteroides* in lactating women^51^. However, the mechanisms underlying vitamin-mediated interactions and the concentrations available in the distal gut, particularly those from dietary intake, remain poorly understood.

As previously shown for *Bacteroides* spp.^52–55^, *S. copri* also engages in nutrient cross-talk with pathogenic and commensal Enterobacteriaceae (Fig. 4). We show that release of simple sugars from polysaccharide degradation, independent of their utilization, is sufficient to drive an *E. coli*-dependent expansion of *S. copri*, indicating a signalling rather than nutritional interaction. Polysaccharide-derived sugars have been shown to function beyond their nutritional role in gut bacteria, acting as interbacterial signalling molecules that directly influence gene expression and colonization capacity^44,55,56^. This signalling appears to be triggered by diverse nutrients; however, the underlying signalling cascade in *E. coli* will require further detailed studies. Interestingly, this mutualistic interaction with *S. copri* is conserved among Enterobacteriaceae. Therefore, the diversity of Enterobacteriaceae species within the microbiome may play an important role in determining *S. copri* establishment and could help create a broader supportive niche in response to diverse dietary components. Indeed, our human stool metagenomics analysis shows that the prevalence, diversity and abundance of Enterobacteriaceae in non-industrialized microbiomes are significantly higher than in industrialized microbiomes. Although we did not observe a correlation between the relative abundance of Enterobacteriaceae and *S. copri*, consistent with previous studies, we did find a significant correlation between the relative abundance of *S. copri* and the number of Enterobacteriaceae species, specifically in non-industrialized microbiomes. The enrichment of Enterobacteriaceae in non-industrialized microbiomes may reflect greater environmental exposure^30,57^. Consistently, analysis of a global food dataset^35^ revealed a higher prevalence of Enterobacteriaceae in foods from several non-industrialized countries, particularly plant-based foods. This aligns with the association of *Segatella* with plant-based diets and its anti-correlation with processed foods in industrialized populations. In addition, Enterobacteriaceae strain exchange can occur with other environmental sources, such as drinking water^33,58^. Finally, our findings suggest that establishing microbiota signatures by dominant gut Bacteroidales goes beyond simple dietary ingredients. It rather involves nutrient-driven commensal cross-talk and competition, as well as environmental exposures that may promote microbiome enrichment.

Synthetic and human-derived faecal communities are valuable models to study microbial networks despite their reduced complexity relative to in vivo systems. Experimental designs such as ours can uncover interactions among xenobiotics, keystone species and primary or secondary degraders, yielding mechanistic insights into microbiome assembly and responses to perturbation. Our findings may also inform dietary interventions that account for both supplemented components and baseline microbiome composition, thereby supporting personalized combinatorial pre- and probiotic strategies.

## Methods

### Bacterial strains and culture conditions

All strains used in this study are listed in Supplementary Table 1.

Gut commensal isolates and community assemblies were cultured in a flexible anaerobic chamber (Coy Laboratory Products) containing 10% CO_2_, 5% H_2_ and 85% N_2_ at 37 °C in liquid modified Gifu Anaerobic broth (mGAM; Hyserve, 05433) and grown on brain heart infusion (BHI; Oxoid, CM1135B) agar (BACTO agar; BD, 214010) supplemented with 10% defibrinated sheep blood (Thermo Scientific, 10516583) and 1 μg ml^−1^ Menadione (vitamin K_3_; Sigma-Aldrich, M5625).

### Defined gut community design and assembly

To select members of the commensal community, isolates were chosen on the basis of several criteria: (1) they are commonly reported as human gut isolates and generally recognized as commensals, (2) they grow well (within 24 h) in mGAM broth under anaerobic conditions and (3) they remain stable within the community after the first passage. Furthermore, the phylum-level composition of the community reflects that of the human gut, with proportional representation including representatives from eight major human gut bacterial families and with a species richness comparable to that of existing model communities. The final community is based on several iterations informed by previous studies and on the successful establishment of *Segatella* sp.^10,41,59–61^.

To assemble communities, strains were grown overnight in mGAM from single colonies. Each strain was adjusted to a final optical density at 600 nm (OD_600_) of 0.01 and pooled into fresh media. After 24 h of growth (P0), the strains were passaged at a 1:100 dilution into fresh mGAM or mGAM supplemented with the corresponding dietary component. Communities were collected at passage 3 (P3), identified as the timepoint when the community composition reached stability. At P3, 1 ml of the culture was centrifuged to pellet the cells, which were then immediately frozen and stored for subsequent analysis, unless otherwise specified. For subsequent competition assays, arabinan, other polysaccharides, and individual simple sugars were supplemented into mGAM at a final concentration of 5 mg ml^−1^. For combinations of sugars, the total concentration of all added sugars was adjusted to 5 mg ml^−1^.

### Human faecal-derived complex communities

All stool donations were obtained with the informed consent of the donors and in accordance with the approved study protocol by the Ethics Committee Lower Saxony (Permit No. 8628_BO_K_2019). Fresh stool was obtained from 8 healthy adult donors enrolled in the ongoing Mikrodivers cohort. Frozen stool from 8 control donors with undetectable Enterobacteriaceae levels in corresponding metagenomic datasets was obtained from the Rheuma-VOR German cohort, which recruited treatment-naïve patients with new-onset rheumatic disorders. All stool samples were processed as previously described^26,61^. Briefly, fresh stool samples (<500 mg) were immediately transferred into an anaerobic chamber and homogenized 1:1 (w/v) with 40% glycerol in PBS supplemented with 0.5 g l^−^^1^ cysteine. The homogenate was stirred thoroughly and aliquoted into 700 µl glycerol stocks. For each donor, one aliquot was inoculated into 50 ml mGAM, serially diluted to 1:1,000 and incubated anaerobically at 37 °C for 24 h. After incubation, the 1:1,000 dilution cultures were used to generate stabilized communities. Glycerol stocks were prepared by mixing 800 µl of culture with 200 µl 50% glycerol and stored at −80 °C. For community stabilization, 1:1,000 dilution cultures were passaged by inoculating 1:100 into fresh mGAM and incubating anaerobically at 37 °C for 24 h. This procedure was repeated for a total of 4 passages. After passage 4, stabilized communities were used for coalescence assays with *S. copri* HDD04 and *E. coli* MR102 strains^62^.

Frozen stool samples were transferred directly into the anaerobic chamber, resuspended 1:1 (w/v; <500 mg) in mGAM, serially diluted to 1:1,000 and incubated anaerobically at 37 °C for 24 h. Subsequent stabilization and passaging steps were performed as described for fresh stool samples.

For community-strain coalescence experiments, *S. copri* HDD04 and *E. coli* MR102 were each inoculated from a single colony into mGAM and grown anaerobically overnight at 37 °C. Overnight strain cultures were then combined with stabilized stool-derived communities at community-to-strain ratios of 1:10 or 1:1,000 in 2 ml of fresh mGAM, either unsupplemented or supplemented with arabinan. Cultures were incubated anaerobically at 37 °C for 24 h. Following the initial coalescence step, assemblies were serially passaged three times by transferring 1:100 into fresh medium and incubating for 24 h at each passage. Data corresponding to the 1:1,000 community-to-strain ratio are presented in Fig. 5 and Extended Data Fig. 5.

### Mouse caecal-derived complex communities

Experiments with mouse caecal material were performed in agreement with the guidelines of the Helmholtz Center for Infection Research, Braunschweig, Germany, the National Animal Protection Law (Tierschutzgesetz (TierSchG)), the animal experiment regulations (Tierschutz-Versuchstierverordnung (TierSchVersV)) and the recommendations of the Federation of European Laboratory Animal Science Association (FELASA). Male and female mice were housed under standard SPF conditions under a 12 h light/12 h dark cycle, with controlled temperature and humidity, and provided ad libitum access to food and water (permit AZ 42508-01-003). Mice were maintained on a standard chow diet and used at 12 weeks of age. SPF mice were killed, and caecal contents were aseptically collected and immediately transferred into an anaerobic chamber. Samples (<500 mg) were resuspended 1:1 (w/v) in mGAM, serially diluted to 1:1,000 and incubated anaerobically at 37 °C for 48 h. Following initial outgrowth, 100-fold dilution cultures were passaged by inoculating 1:100 into fresh mGAM and incubated anaerobically at 37 °C for 48 h. Passaging was repeated for a total of 3 passages to stabilize the caecum-derived communities.

Community-strain coalescence assays with *S. copri* HDD04 and *E. coli* MR102 were performed as described for human stool-derived communities. Stabilized mouse communities were combined with strain cultures at community-to-strain ratios of 1:10 and 100:1 in fresh mGAM and incubated for 24 h. Cultures were serially passaged 3 times at 1:100 dilutions for 24 h. Data corresponding to the 1:10 community-to-strain ratio are shown in Fig. 5.

### Screen of dietary components

The stock solutions of the dietary components were prepared as indicated in Supplementary Table 2. To prepare the supplemented media, 2× mGAM, dissolved in MilliQ water and filter sterilized, was pooled with the stock solution of the dietary compound (v/v) (Supplementary Table 2). Dietary glycans were prepared as described and used at final concentrations ranging from 0.04% to 0.2%, adjusted on the basis of properties such as gelling, solubility, viscosity and accessible amounts. Vitamins were prepared separately as indicated and used at concentrations corresponding to those previously tested in vivo^63^. The media were aliquoted into sterile 96-deep-well plates and inoculated at 1:100 with the community assembly previously grown for 24 h in mGAM (P0). The plates were covered with breathable membranes and passaged at 1:100 into fresh media until passage 3 using an Eppendorf epMotion 96 with mixing. The endpoint OD_600_ at each passage was measured for all conditions and normalized to media-only background.

### Flow cytometry analysis

For flow cytometry analysis, 500 μl of community culture was collected at P3 in microcentrifuge tubes and washed twice with 1 ml PBS (2,200 × *g*, 4 min at 4 °C). After the second wash, cells were resuspended in 1 ml propidium iodide (PI)/DAPI (1 µg ml^−1^) solution, 25 μl of cell counting control beads were added, and the cells were gated on the basis of forward and side scatter to exclude debris. Viability gating was performed using DAPI-positive cells to identify total bacteria and PI staining to distinguish dead or membrane-compromised cells (PI-positive). Absolute cell counts were calculated by comparing the number of bacterial events to the known concentration and number of counting beads detected, according to manufacturer instructions.

### Growth on sole carbon source

The stock solutions for growth on complex dietary glycans as sole carbon source were prepared as indicated in Supplementary Table 2. A 2× concentration of filter-sterilized minimal media was prepared as in ref. ^19^ and pooled with each dietary glycan stock solution (v/v). Minimal media were prepared as follows: 100 mM KH_2_PO_4_ (pH 7.2), 15 mM NaCl, 8.5 mM (NH_4_)_2_SO_4_, 100 μM MgCl_2_, 1.4 μM FeSO_4_·7H_2_O, 50 μM CaCl_2_, 1.9 μM haematin, 1 mg l^−1^ vitamin K3, 5 μg l^−1^ vitamin B12, 0.5 g l^−1^ cysteine. In addition, it contains 10 ml l^−1^ amino acid mix solution (250 mg each of l-alanine, l-arginine, l-asparagine, l-aspartic acid, L-cysteine, L-glutamic acid, L-glutamine, glycine, L-histidine, L-isoleucine, L-leucine, L-methionine, L-phenylalanine, L-proline, L-serine, L-threonine, L-tryptophan, L-tyrosine, L-valine and 312 mg of L-lysine monohydrochloride into 1 l distilled water), 10 ml l^−1^ purine/pyrimidine solution (200 mg each of adenine, guanine, thymine, cytosine and uracil into 1 l distilled water, pH 7.0), 10 ml l^−1^ ATCC Vitamin Mix, 10 ml l^−1^ ATCC Trace Mineral Mix and 1 μl l^−1^ vitamin K1. The commensal strains were grown overnight in mGAM from single colonies and inoculated into the minimal media at a starting OD_600_ of 0.05 in 96-well plates. The cultures were grown for 70 h anaerobically at 37 °C with OD_600_ measurement at 1-h intervals after 45 s of orbital mixing using a Biotech Epoch2 reader.

To calculate the percent growth, the OD_max_ at each condition was calculated as a fraction of the OD_max_ for each strain in mGAM (Supplementary Table 8):

1 $$\mathrm{Percent\; growth}=({\mathrm{OD}}_{\mathrm{maxminimalmedia}}/{\mathrm{OD}}_{\mathrm{maxmGAM}})\times 100$$

To account for background growth, we averaged the extent of residual growth across species and conditions and defined >25% growth relative to mGAM as a threshold for specific utilization of a polysaccharide. A few substrates such as gellan, pachyman, β-D-glucan, nigeran, HM pectin, kefir gum and inulin (which shows growth patterns similar to fructooligosaccharides), exhibited robust growth across most species, suggesting higher levels of contaminating simple sugars. Overall, for the majority of polysaccharides, contaminating sugars are unlikely to be a major confounding factor, as they are rapidly depleted and do not support sustained or robust growth.

*E. coli* strains were grown in minimal M9 media (90 mM Na_2_HPO_4_, 22 mM KH_2_PO_4_, 8.6 mM NaCl, 18.8 mM NH_4_Cl, 2 mM MgSO_4_, 0.1 mM CaCl_2_, 0.5% carbon source). The cultures were grown for 24 h anaerobically at 37 °C with OD_600_ measurement at 1-h intervals after 45 s of orbital mixing using a Biotech Epoch2 reader.

For growth rate calculation, the exponential phase was identified for each strain under each condition. OD_600_ values were log transformed and fitted by linear regression against time with *R*^2^ ≥ 0.98–0.99, and the slope ($\mu =\frac{\Delta\mathrm{ln}({\mathrm{OD}}_{600})}{\Delta{t}}$) was determined to calculate the specific growth rate (h^−^^1^).

### Transwell co-culture experiments

Strains were grown overnight in mGAM from a single colony and used to inoculate fresh mGAM or mGAM supplemented with arabinan at a starting OD_600_ of 0.05. The 6 Bacteroidaceae strains were pooled to a final starting OD_600_ of 0.05. Co-culture experiments were conducted using the Cerillo Duet Co-Culture Platform (CCU1000), which enables real-time monitoring of bacterial interactions while maintaining fluidic contact between compartments through a porous physical barrier (0.2 µm size exclusion filter). The Duet devices were inoculated with the respective bacterial strains designated for co-culture (800 µl volume per well). The plates were incubated for 48 h at 37 °C with OD_600_ measurement at 1-h intervals after 45 s of orbital mixing using a custom Cerillo plate template on a Biotech Epoch2 reader. Area under the curve (AUC) for each growth curve was calculated using GraphPad Prism 10 with a *Y* = 0 baseline for all curves.

### Genomic DNA extraction and amplicon 16S rRNA sequencing

Genomic DNA was extracted from community samples using the ZymoBIOMICS DNA Miniprep kit, following manufacturer protocol. For 16S rRNA amplicon sequencing, the V4 hypervariable region of the 16S rRNA gene was amplified using primers F515 (GTGCCAGCMGCCGCGGTAA) and R806 (GGACTACHVGGGTWTCTAAT), on the basis of established protocol^64^. Briefly, input DNA was normalized to 25 ng μl^−1^ before PCR amplification, with unique 12-base Golay barcodes incorporated via specific primers (Sigma) to enable sample multiplexing.

Following PCR, amplicons were pooled and normalized to 10 nM before sequencing on an Illumina MiSeq platform using 250 bp paired-end reads (PE250) or on an Illumina NextSeq platform using 150 bp paired-end reads (PE150). Raw sequencing reads were demultiplexed using idmp (https://github.com/yhmu/idemp) according to barcode sequences. Library processing included merging paired-end reads, quality filtering, dereplication, singleton removal, denoising and chimaera checking using the USEARCH pipeline v.11.0.66751.

Specifically, reads were merged with the ‘fastq_mergepairs’ command (parameters: maxdiffs=30, pctid=70, minmergelen=200, maxmergelen=400), followed by quality filtering with ‘fastq_filter (maxee=1)’. Unique sequences were identified using ‘fastx_uniques’ with a minimum unique size of 2. Biological sequences (ASVs/zOTUs) were predicted and chimaeras removed using ‘unoise3’ (parameters: minsize=10, alpha=2). Amplicon quantification was performed with ‘usearch_global (strand=plus, ID=0.97, maxaccepts=10, top_hit_only, maxrejects=250)’.

Taxonomic assignment was carried out using ‘Constax2’, which integrates classifiers RDP, SINTAX and BLAST, referencing the GreenGenes database. Resulting taxonomic data were summarized into a biom file for visualization in phyloseq and further downstream analyses. Operational taxonomic units (OTUs) with an abundance below 0.5% across all samples were excluded. For synthetic communities, a closed-reference database comprising the defined community members was employed for classification.

### Genetic manipulation of *E. coli*

Deletion strains were generated by standard gene replacement as previously described^65^. Briefly, antibiotic resistance cassettes carrying either KmR or CmR resistance genes were amplified from pKD4 and pKD3, respectively^65^. Primers used include a 40-bp sequence complementary to the region in which the insertion was desired. Purified fragments were electroporated into strains carrying pKD46. Positive clones were selected in the presence of the required antibiotic and validated by genotype PCR. To create multiple loci mutants in *E. coli* Nissle, phage P1 lysates from single mutant strains were generated, and mutations were mobilized into *E. coli* Nissle by P1 transduction as described previously^66^. When required, the antibiotic cassette was removed by the expression of Flp recombinase from pCP20 as previously described to generate markerless gene deletions^67^. Oligonucleotides used for strain construction are listed in Supplementary Table 9.

### qPCR quantification

The relative abundance of all bacteria was monitored at indicated timepoints by qPCR of purified DNA using species-specific primers targeting unique sequences within single-copy genes. qPCR was performed using a CFX96 instrument (BioRad) and SYBR FAST universal master mix (KAPA Biosystems). Mean strain quantities were determined using standard curves generated with primers on purified genomic DNA from each strain. Standard curves showed amplification efficiencies >85%, slopes between –3.1 and –3.8, and *R*^2^ > 0.98. Relative changes were calculated using the efficiency-corrected Δ*C*_q_ method^68^. All primer sequences used in this study are listed in Supplementary Table 9.

### Competitive index calculation

The competitive index between Prevotellaceae (*Segatella copri*) and Bacteroidaceae (6 species) was calculated using their relative abundances derived from 16S amplicon sequencing data, or as absolute abundances using DNA concentrations obtained from qPCR quantification. To account for zero values arising from the absence of taxa, zeros were replaced with a small pseudocount (10^−^^6^) before calculation according to the following equation:

2 $$\mathrm{Competitive}\,\mathrm{index}=\mathrm{log}\,\frac{(S.\,{copri}\,\mathrm{abundance}+0.000001)}{(6\,\mathrm{Bacteroidaceae}\,\mathrm{abundance}+0.000001)}$$

### RNA processing and metatranscriptomics sequencing

RNA was processed and sequencing performed as in ref. ^44^. Briefly, communities were collected after 8 h and 20 h of growth after passaging in mGAM and mGAM+arabinan, and a biomass of 4 units of OD_600 nm_ was collected 5:1 (v/v) with stop solution (95% ethanol, 5% phenol) and snap frozen in liquid nitrogen. Bacterial total RNA was extracted using the hot phenol method followed by DNase treatment. Quality of the RNA was determined using a bioanalyser at the genome analytics core facility of the Helmholtz Centre for Infection Research (HZI).

For metatranscriptomics sequencing, three biological replicates per condition were processed. Total RNA was subjected to ribosomal RNA depletion by Ribo-Zero Plus Microbiome rRNA Depletion kit (Illumina), and cDNA libraries were generated using Illumina Stranded Total RNA Prep (Illumina) following manufacturer instructions.

Generated libraries were sequenced at ~100–200 million reads sequencing depth, paired-end 2 × 150 bp. To obtain species-specific mapped reads, reads were aligned to the genomes of the specific members in community assemblies using the READemption (2.0.0) and the ‘–crossalign_cleaning’ command to remove reads mapped to multiple species^69^. For each species, differentially expressed genes between mGAM and mGAM+arabinan conditions were calculated using DESeq2 (ref. ^70^) which utilizes the Wald test to determine the *P* value and the Benjamini–Hochberg method to correct *P* values for multiple testing (*P*_adj_)^71^.

### Targeted metabolomics of communities

For metabolomics analysis, the isotope ratio approach via LC–MS/MS was used^71^. Briefly, communities were collected after 20 h of growth following passaging in mGAM or mGAM+arabinan. A biomass OD_600_ of 1 was collected, diluted in chilled PBS to OD_600_ of 0.5, vacuum filtered (0.45 μm; Merck Millipore, HVLP02500), and metabolites were extracted by transferring the filters into pre-chilled 40:40:20 (v/v/v) acetonitrile (Honeywell, 14261-1l)/methanol (VWR, 83638.320)/water at −20 °C for 30 min. Extracts were centrifuged (15 min, 16,000 × g, −9 °C), and supernatants were stored at −80 °C until LC–MS/MS analysis. Relative metabolite concentrations were calculated from the ^12^C/^13^C isotope signal ratio using internal standards (Supplementary Table 4), log_10_ transformed for statistical analyses and converted to *z*-scores for multivariate analysis. Metabolite and pathway enrichment analyses were performed in MetaboAnalyst 6.0 (https://www.metaboanalyst.ca) using KEGG IDs and the *Escherichia coli* K-12 MG1655 pathway database, with significance determined by multiple *t*-tests and false discovery rate correction.

### Metagenomics data analysis

#### *S. copri* and Enterobacteriaceae global distribution

To assess the global prevalence, richness and abundance of *S. copri* and Enterobacteriaceae in human populations, we analysed 870 publicly available shotgun metagenomes (Supplementary Table 6) from 9 countries representing diverse lifestyles. Using MetaPhlAn4, the vJan25_CHOCOPhlAnSGB_202503 marker genes database with default settings^72^, we profiled microbial species and estimated relative abundances, retaining only species with ≥0.0001 abundance to reduce low-abundance noise. All samples were selected from healthy adult males and females (16–65 years), defined as free from self-reported diseases or medical interventions, to represent general populations. Each metagenome was classified as originating from either an industrialized or non-industrialized population (Supplementary Table 6). Briefly, the term ‘non-industrialized’ describes a population that practices a traditional lifestyle, characterized by factors such as diet, hygiene and medical care, whereas ‘industrialized’ describes a population that is exposed to a process of incremental urbanization transitioning to a controlled food production chain, increased hygiene and accessibility to modern medicine. The concept of ‘industrialized’ and ‘non-industrialized’ was adopted here to demarcate populations by combining at least the majority of the factors discussed above, as described previously^73,74^.

#### Analysis of food-borne Enterobacteriaceae

To identify members of the Enterobacteriaceae family in food samples, we used samples from curatedFoodMetagenomicData (cFMD, v.1.3.0) (https://github.com/SegataLab/cFMD/releases/tag/v1.3.0)^35^. Briefly, cFMD is a metagenomic resource that integrates 3,310 food metagenomes from 52 countries, covering 15 food categories which can be further grouped into 118 types or 337 subtypes. Food categories were classified into plant- (fermented tubers and roots, fermented fruits and vegetables, fermented seeds, fermented legumes, fermented grains, fruits and vegetables) or animal-based (dairy, fish, meat, fermented meat). Samples not assigned to any specific food category were removed from the analysis (*n* = 8). In addition, samples assigned to the category ‘fermented fish’ were removed due to low sample size (*n* = 5). Finally, we removed 30 food metagenomes that contained spiked-in *Escherichia coli*. Taxonomic profiling and calculation of relative abundances were performed using MetaPhlAn4, the vJan25_CHOCOPhlAnSGB_202503 marker genes database. Only taxa belonging to Enterobacteriaceae were retained.

#### Evaluating enrichment of SGBs to food categories and lifestyle

To evaluate the enrichment of species-level genome bins (SGBs) assigned to Enterobacteriaceae in specific food categories as well as lifestyle (industrialized vs non-industrialized), Fisher’s exact test was performed. Joint enrichment in food categories and lifestyle was assessed by combining *P* values from both enrichment tests using Stouffer’s *Z*-score method. *P* values were adjusted for multiple hypothesis testing using the Benjamini–Hochberg procedure. An SGB was considered directionally concordant and jointly enriched if FDR-adjusted *q* < 0.01 and when the odds ratio was greater than 1 in the food-associated model and in the lifestyle model (with respect to non-industrialized).

#### Functional profiling of Enterobacteriaceae carbohydrate metabolism across lifestyle

To evaluate the functional profile of Enterobacteriaceae in industrialized and non-industrialized contexts, we used HUMAnN 3 (refs. ^75,76^) with a UniRef50 reference database (v.2019_06) to obtain gene family-level functional profiles. Per-sample gene family abundances were normalized to counts per million (CPM) using ‘humann_renorm_table’, and normalized tables were merged across samples using ‘humann_join_tables’. UniRef50 gene families were taxonomically stratified, and only those assigned to Enterobacteriaceae members were retained. A curated mapping of carbohydrate metabolic pathways to UniRef50 identifiers (Supplementary Table 7)^77^ was used to define pathway-level gene sets. For each sample and each taxon, pathway abundance was computed by summing the CPM-normalized abundances of all UniRef50 gene families assigned to that pathway and attributed to that taxon. We compared the relative pathway abundance within each taxon between industrialized and non-industrialized samples using Mann–Whitney *U*-test. Relative pathway abundance was defined as the pathway CPM divided by the total CPM-normalized gene family abundance attributed to that taxon in the same sample, considering only those samples in which the taxon is present. All *P* values were corrected post hoc using the Benjamini–Hochberg procedure.

### Statistics and analysis

Statistical analysis was performed using GraphPad Prism 10, R 4.5.0 (www.r-project.org) and Python 3.8. Details of statistical tests used, sample size indicated as ‘*n*’, and definition of means, error bars and significance are provided in figure legends and in methods details.

### Reporting summary

Further information on research design is available in the Nature Portfolio Reporting Summary linked to this article.

## Supplementary information

Supplementary Information Supplementary Results.

Reporting Summary

Peer Review File

Supplementary Tables 1–9 Supplementary Table 1 List of bacteria. Table 2a List of dietary food components used in this study. Table 2b, List of food components supplemented in the community. Table 2c List of complex dietary glycans used as sole carbon source for growth. Table 3a *Segatella copri* HDD04 gene expression at 20 h within 21-community assemblies (arabinan vs mGAM). Table 3b *Bacteroides thetaiotaomicron* gene expression at 20 h within 21-community assemblies (arabinan vs mGAM). Table 4 Targeted metabolite relative concentrations ^12^C/^13^C ratio. Table 5a *Escherichia coli* MR102 gene expression at 8 h within community assemblies. Table 5b *Segatella copri* HDD04 gene expression at 8 h within community assemblies. Table 5c *Bacteroides fragilis* gene expression at 8 h within community assemblies. Table 5d *Bacteroides thetaiotaomicron* gene expression at 8 h within community assemblies. Table 5e *Phocaeicola vulgatus* gene expression at 8 h within community assemblies. Table 6a Cohort of healthy adults with different lifestyles used in this study from published datasets. Table 6b Samples from cohort of healthy adults with different lifestyles used in this study from published datasets. Table 7 List of genes for simple carbohydrate utilization in Enterobacteriaceae (from ref. ^77^). Table 8 OD_max_ of each of the 21 community species in minimal media with each polysaccharide and in mGAM. Table 9 List of primers.

## Source data

Source Data Fig. 1 Statistical source data.

Source Data Fig. 2 Statistical source data.

Source Data Fig. 3 Statistical source data.

Source Data Fig. 4 Statistical source data.

Source Data Fig. 5 Statistical source data.

Source Data Extended Data Fig. 1 Statistical source data.

Source Data Extended Data Fig. 2 Statistical source data.

Source Data Extended Data Fig. 3 Statistical source data.

Source Data Extended Data Fig. 5 Statistical source data.

## Data Availability

The RNA sequencing and 16S rRNA amplicon sequencing datasets generated in this study have been deposited in the NCBI Sequence Read Archive (SRA) under BioProject accession PRJNA1283124. The strains used in this study are available from T.S. upon request, and publicly available strains can be obtained from the German Collection of Microorganisms and Cell Cultures (DSMZ). Source data are provided with this paper.
