# Supplementary material for: Synergy between Enterobacteriaceae and diet mediates competition between dominant Bacteroidales in the human gut
Source: Nat Microbiol. 2026 Oct 2;11(11):3074–89. doi: 10.1038/s41564-026-02500-6 (PMC13739738; doi:10.1038/s41564-026-02500-6)
Supplement: Supplementary file 1 [file 41564_2026_2500_MOESM1_ESM.pdf]

nature microbiology

Supplementary information

https://doi.org/10.1038/s41564-026-02500-6

Synergy between Enterobacteriaceae and diet mediates competition between dominant Bacteroidales in the human gut

In the format provided by the authors and unedited

Supplementary Information

Synergy between Enterobacteriaceae and diet mediates competition between dominant Bacteroidales in the human gut

Caroline Tawk, Youssef El Mouali, Kun D. Huang, Sebastiaan Valkiers, Vitor Heidrich, Achim Gronow, Lisa Osbelt, Johanna Rapp, Hannes Link, Nicola Segata, Athanasios Typas, Till Strowig

Metabolomics and metatranscriptomics of synthetic communities reveal cross-feeding between Bacteroidales and E. coli

To uncover potential nutrient exchange mechanisms, we investigated the contribution of the different families, Prevotellaceae (*S. copri*), Bacteroidaceae (six species), and Enterobacteriaceae (*E. coli*) to the metabolic and transcriptomic landscape of the communities. First, we performed targeted metabolomics on the different community assemblies with or without arabinan supplementation. We analyzed *S. copri* with the six Bacteroidaceae in the presence of *E. coli* ("with *S. copri*"), the six Bacteroidaceae in the presence of *E. coli* without *S. copri* ("without *S. copri*"), and *S. copri* with six Bacteroidaceae in the absence of *E. coli* ("without *E. coli*") (Fig. 3d). Consistent with our previous results, only in the presence of *E. coli*, the community switches from Bacteroidaceae-dominated to *S. copri*-dominated when supplemented with arabinan, whereas the absence of *E. coli* or *S. copri* leads to a Bacteroidaceae-dominated community irrespective of arabinan supplementation (Fig. 3d). Consequently, we observed the largest number of significantly changed metabolic pathways, based on changes in the relative concentrations of associated metabolites, when arabinan is supplemented in the community that includes both *S. copri* and *E. coli* ("with *S. copri*") (Extended Data Fig. 3c; Supplementary Table 4). Metabolic pathways involved in amino acid metabolism, vitamin biosynthesis, the TCA cycle, glycolysis/gluconeogenesis, and redox metabolism were enriched in the community "with *S. copri*", which suggests these metabolites are contributed by the expansion of *S. copri* (Extended Data Fig. 3c). Fewer metabolic pathways were enriched in the community "without *S. copri*" in the presence of arabinan. However, several pathways such as purine and pyrimidine metabolism, amino acid metabolism, and nicotinate/nicotinamide metabolism were consistently enriched in

communities containing *E. coli* ("with *S. copri*" and "without *S. copri*"), but not in communities lacking *E. coli* ("without *E. coli*"). This pattern suggests that the enrichment of these metabolic functions is likely driven by the presence of *E. coli* in the community (Extended Data Fig. 3c). The community "without *E. coli*" showed a minor metabolic response to arabinan, reflecting the minor changes in the Bacteroidaceae-dominated community (Fig. 3d; Extended Data Fig. 3c). These results indicate that the shift from Bacteroidaceae to a *Segatella*-dominated configuration, modulated by arabinan and the presence of *E. coli*, is the primary factor influencing the metabolic landscape.

Next, harnessing the advantage of defined communities, we performed metatranscriptomics and captured the gene expression of the individual members specifically. We analyzed the transcriptomic signatures of the individual bacterial families within these community assemblies to gain deeper insight into the interactions between the different species. The analysis of *S. copri* gene expression in the presence of arabinan in the community "with *S. copri*" at 8h showed an upregulation of various nutrient acquisition genes including PULs, transporters, glycosyl hydrolases, tryptophan biosynthesis, vitamin biosynthesis, and genes of unknown function, and a downregulation of genes in PUL13, various amylase genes, and DNA-binding proteins, which is consistent with the metabolomics results and the shift from starch to arabinan utilization (Extended Data Fig. 4a; Supplementary Table 5). Notably, genes in the *S. copri* arabinan PUL14 were significantly upregulated at 8h in the presence of arabinan (between 1.8-1.9 fold-change; padj$<$0.0001) (Supplementary Table 5). Further, we analyzed the gene expression of the three most abundant Bacteroidaceae species in the community assemblies: *B. thetaiotaomicron*, *B. fragilis*, and *P. vulgatus*. Overall, the gene expression changes between the different Bacteroidaceae in the presence of arabinan in the different community assemblies were similar at 8h (Extended Data Fig. 4b-d; Supplementary Table 5). All three Bacteroidaceae differentially regulated nutrient acquisition genes, including PULs, transporters, and glycosyl hydrolases, while *B. fragilis* additionally upregulated fimbrial genes (Extended Data Fig. 4b-d; Supplementary Table 5).

Cross-feeding and sugar-dependent signaling between S. copri and E. coli

We have conducted additional experiments to gain deeper mechanistic insight into the interaction between *E. coli* and *S. copri* within the community. Our data suggest that *E. coli*'s influence on community dynamics is unlikely to happen through the inhibition of Bacteroidaceae. In our co-culture transwell assays, no inhibition of Bacteroidaceae growth was observed under any condition

(Extended Data Fig. 7a). Both *S. copri* and the Bacteroidaceae benefit from *E. coli* in co-culture, with *S. copri* showing particularly enhanced growth in the presence of arabinan (Figure 3C and Extended Data Fig. 3a,b). Of note, when the Bacteroidaceae are co-cultured with both *E. coli* + *S. copri*, their growth is similar to that observed in the absence of *E. coli* (Extended Data Fig. 7a). This suggests that *E. coli* may provide a shared beneficial factor that *S. copri* and Bacteroidaceae compete for, with a preferred polysaccharide conferring a competitive advantage to *S. copri*.

To identify the molecules underlying these dynamics, we tested tryptophan since arabinan conditions could increase tryptophan levels by downregulating of tryptophanase (tnaA) in *E. coli*, according to our metatranscriptomics data. Additionally, metabolomics showed enrichment of the tryptophan metabolism pathway, including tryptophan, in arabinan in the presence of E. coli (Extended Data Fig. 3c; Supplementary Table 4). However, supplementation with tryptophan or indole (0.5 g/L) did not confer a competitive advantage to *S. copri*. Moreover, *E. coli* Nissle mutants lacking tnaA or the tryptophan biosynthesis gene trpE still promoted *S. copri* expansion similarly to wild type (Extended Data Fig. 7b,c), suggesting tryptophan metabolism is not the driving factor.

To explore mechanisms beyond tryptophan metabolism, we examined other pathways highlighted by our metatranscriptomics and metabolomics data and tested known *S. copri* auxotrophies and essential growth components. We generated additional *E. coli* Nissle mutants targeting cytochrome c biogenesis ATP-binding export protein ccmA, which was strongly upregulated in arabinan, and the serine acetyltransferase cysE, required for cysteine biosynthesis, also recently shown to be implicated in cross-feeding between *E. coli* and *Bifidobacteria* (https://doi.org/10.1101/2025.03.24.645027). However, both mutants still promoted *S. copri* expansion similarly to wild type (Extended Data Fig. 7b). Supplementation with cysteine, hemin, or hematin also had no effect (Extended Data Fig. 7c).

We next tested broader nutrient limitations by supplementing amino acids, purines/pyrimidines, trace elements, glutathione, or coenzyme A, but none conferred a competitive advantage to *S. copri*. Likewise, supplementation with succinate (pH 5.0) or lactate did not reproduce the *E. coli*-mediated benefit (Extended Data Fig. 7c).

To determine whether *E. coli* utilization of the polysaccharide-derived simple sugars is required to promote *S. copri* expansion, we generated a quadruple *E. coli* mutant (Δ*araBAD* Δ*rhaBAD* Δ*galTK* Δ*uxaCT*) unable to metabolize the arabinan-derived monosaccharides (arabinose, rhamnose, galactose, and galacturonic acid). Interestingly, the *E. coli* quadruple mutant still promoted *S. copri*

expansion in the presence of arabinan or the derived simple sugars, despite its inability to utilize these substrates (Figure 3h; Extended data Fig. 5b). This result is similarly observed with two additional fibers that promote *S. copri* expansion in an *E. coli*-dependent manner, arabinoxylan and polygalacturonic acid, along with their derived simple sugars and the corresponding *E. coli* mutants Δ*araBAD* Δ*xylAB* and Δ*uxaCT*, respectively (Extended data Fig. 7d). Importantly, growth in minimal M9 media confirmed that no other simple-sugar contaminants in the fibers supported appreciable growth of these strains (<30% compared to glucose; Extended data Fig. 7e). Together, these results indicate that the presence of simple sugars is sufficient to drive *S. copri* expansion within the community, independently of their metabolism, suggesting a signaling rather than nutritional interaction.

Dietary component interaction with S. copri is conserved among Enterobacteriaceae species

Although utilization of simple sugars is not required for *E. coli*-mediated promotion of *S. copri*, their presence nonetheless triggers an expression of the sugar-specific pathways in *E. coli* (Figure 3) and drives an *E. coli*-dependent expansion of *S. copri* within the community. We therefore reasoned that genes involved in simple-sugar transport, sensing, and utilization pathways would be important for this phenomenon. We performed an expanded metagenomic analysis to identify Enterobacteriaceae genes involved in the utilization, transport, and sensing of simple sugars derived from dietary polysaccharides, including those from the genomes of the strains tested experimentally in this study (Figure 4d). We found that these genes are broadly conserved across Enterobacteriaceae for most of the sugars examined (Extended Data Fig. 7f), suggesting that this sugar-dependent response is widely conserved and likely occurs across diverse Enterobacteriaceae in response to a broad range of fiber-derived sugars.

Identifying potential strain sharing between the human gut microbiome and foods

To assess potential sharing between the human gut microbiome and foods, we performed strain profiling of Enterobacteriaceae SGBs in food and stool samples from Industrialized and Non-Industrialized cohorts. We selected SGBs enriched in both environments and reconstructed strain-level phylogenies. Notably, closely related strains of *E. coli* and *Enterobacter hormaechei* were identified in both human and food samples, indicating the presence of highly similar Enterobacteriaceae across these samples (Extended Data Fig. 7h). We did not detect identical

strains shared between datasets, which is expected given their independent sampling across different times and locations; resolving direct sharing will require studies with matched human-food sampling. The directionality of transmission and persistence of these strains in the gut will require targeted design and longitudinal data.

Correlation of Enterobacteriaceae with Prevotellaceae in a Swedish cohort

Kovatcheva-Datchary et al. [78] reported a 3-day dietary intervention in which healthy Swedish participants consumed barley kernel-based bread (BKB), showing that higher *Prevotella*/*Bacteroides* ratios were associated with improved glucose metabolism. We extracted average relative abundances of *S. copri*, Bacteroidaceae, and Enterobacteriaceae in responders and non-responders. Consistent with the cohort's geographic context, Enterobacteriaceae richness was very low (mean = 1 species/group; maximum = 3), and *E. coli* was not detected in any group, likely reflecting limited sequencing depth rather than a true absence. As reported, responders had higher baseline *S. copri*, which increased after intervention, whereas non-responders showed higher baseline Bacteroidaceae with a modest post-intervention increase (Extended Data Fig. 7i). Enterobacteriaceae were higher at baseline in non-responders but decreased after intervention; conversely, responders exhibited a marked post-intervention increase in Enterobacteriaceae despite low baseline levels.

Although this dataset offers a valuable snapshot of the relationship between dietary fiber and *Prevotella*/*Bacteroides* dynamics, the low Enterobacteriaceae detection limits interpretation. Larger cohorts and deeper sequencing will be needed to resolve these interactions in future dietary interventions.
