# Supplementary material for: Synergy between Enterobacteriaceae and diet mediates competition between dominant Bacteroidales in the human gut
Source: Nat Microbiol. 2026 Oct 2;11(11):3074–89. doi: 10.1038/s41564-026-02500-6 (PMC13739738; doi:10.1038/s41564-026-02500-6)
Supplement: Supplementary file 3 [file 41564_2026_2500_MOESM3_ESM.pdf]

nature portfolio

Peer Review File

Synergy between Enterobacteriaceae and diet mediates competition between dominant Bacteroidales in the human gut

Corresponding Author: Professor Till Strowig

Version 0:

Reviewer comments:

Reviewer #1

(Remarks to the Author)
The manuscript by C. Tawk et al. addresses a highly compelling and important question regarding the dietary and ecological determinants of Prevotellaceae versus Bacteroidaceae dominance in the human gut microbiome. Using a synthetic microbial consortium model, the authors present two key findings: (i) Segatella copri markedly outcompetes several Bacteroides and Phoaceicola strains on certain carbohydrate substrates, particularly arabinan (as well as on some B vitamins), whereas the reverse is observed on other carbon sources; and (ii) this competitive dynamic is contingent on the presence of E. coli (or other Enterobacteriaceae members) within the consortium.
Despite the inherent limitations of in vitro co-culture systems, these findings are corroborated by large-scale metagenomic analyses of microbiomes from Westernized (Bacteroidaceae-dominated) and non-Westernized (Prevotellaceae-dominated) populations, as well as microbial metagenomic profiling of different food products from diverse geographic regions.
While the mechanistic basis of these observations remains only partially elucidated, the study is rigorous, impactful, and of broad relevance. I recommend publication following some revisions.

Line 117: "… vitamin C, which exhibited antimicrobial activity…". This is most likely an artifact caused by a substantial drop in pH. The Gifu Anaerobic Medium (GAM) is not strongly buffered, and according to the supplementary tables, vitamin C was added in a form of ascorbic acid rather than sodium ascorbate, which is much more pH-neutral. This may deserve a brief comment to overcome a potentially wrong (at least not sufficiently justified) interpretation (about a genuine antimicrobial activity of ascorbate).

Line 122-123. A strong impact of B-vitamins (B3, B5 and somewhat B6) on relative abundance of S. copri is mysterious and deserves at least some discussion. Indeed, all strains of S. copri are prototrophs for (can synthesize de novo) all B-vitamins except B7 (biotin) and B12 (cobalamin). Moreover, the detailed genomic reconstruction of B vitamin biosynthesis (eg see [PMID: 31275260]) clearly shows that S. copri does not have a machinery to salvage B3 (no pncAB genes, no niacin transporters), and does not have known B5 uptake transporters. Therefore, the effect of B-vitamins on S. copri fitness cannot be a direct one (which could be a subject of direct testing), but it is rather mediated by the community. (Notably neither B7 nor B12 have any growth-promotion effect on S. copri suggesting that they are present in sufficient quantities in the media). At the same time, while E.coli is also prototrophic for all B-vitamins (except B12), it can potentially benefit from the addition of B-vitamins as it has a metabolic machinery to salvage both B3 and B5 from the medium. While the presence of B3 and B5 did not affect the abundance of E. coli, it could potentially boost some of its (yet unknown) metabolic pathways that have a hypothesized stimulatory effect on S. copri.

Line 138-149. "… the capacity of the different species to utilize the glycans did not predict the direction of Prevotellaceae/Bacteroidaceae ratios within the community". This statement might be too extreme and worth softening by saying something like "…did not always predict the direction…". Indeed, it is supported by two examples of substrates utilizable by both Segatella and Bacteroidaceae strains. In both examples (pectic galactan and arabinan), their relative abundance would be driven by quantitative factors (efficiency of utilization, intrinsic growth rate), and it certainly cannot be predicted by a simple qualitative reasoning (even in the absence of community cross-talk). In that regard, it would be useful to consider an alternative case of a substrate(s), which is preferentially utilized by Bacteroidaceae strains and not Segatella. For example, pullulan (as far as could be deduced from the Extended Data Figure 1D) seems to fall in this category? Likewise, the statement: "These experiments demonstrate that assessing the utilization capacity of dietary components by individual commensal species in monoculture does not reliably predict the overall community dynamics. Other factors of bacterial cross-talk…" (Lines 144-146) does not seem fully justified (even if correct per se) in this context, because it is based on qualitative (growth/no growth in isolation) rather than quantitative (growth rates-based) reasoning.

Line 273. "… observed a downregulation of tryptophanase genes, including tnaA…". Why plural? There is only one tryptophanase in E.coli, and it is encoded by a single gene tnaA.

Line 278-280 (and beyond). While the cross-feeding of monosaccharides, products of extracellular hydrolysis of arabinan by

Bacteroidales, was clearly demonstrated and mechanistically rationalized as beneficial for E. coli, the mechanisms that "leads to the expansion of S. copri" remain elusive. It should be directly admitted, and it would be beneficial to propose and discuss some hypotheses (possibly in Discussion). Minimally, one can hypothesize that a significant metabolic shift observed in E. coli upon cross-feeding arabinan-originating monosaccharides leads to excretion of (yet unknown) metabolite(s) that selectively promotes Segatella (and/or suppress Bacteroides?). A role of this hypothetical metabolite would be rather in signaling than in nutritional cross-feeding. However, the identification of its nature would require dedicated studies (like metabolomic analysis of media (as opposed to whole-cells), fractionation/testing the growth-stimulatory effect of fractions, screening of E. coli KO library, etc). None of it is requested of authors except for some candid discussion. (On the other hand, what if tnaA downregulation is a hint, which, for example, would lead to increased levels of tryptophan, which may cross-feed to Segatella and impact its growth rate if endogenous tryptophan synthesis is not efficient enough?)

Line 420: "Our results show that similarly S. copri engages in mutualistic cross-feeding with E. coli" As already emphasized above, these data are not sufficient to claim "mutualistic cross-feeing". While the data suggest that S. copri likely provides monosaccharides that support E. coli growth (and Bacteroides apparently do the same), it is not shown which metabolites (if any) E. coli provides in return. Therefore, without additional evidence, the observed phenomena may be at best referred to as "possible (hypothesized) mutualistic cross-feeding".

Lines 593-602: section "Growth on sole carbon sources" lacks some essential details. For example, the composition of "minimal media " was not specified. Was it the same for all species? Unlikely so, unless it at least included a number of B vitamins, and it is essential to know which ones and in what quantities?

Line 322 and beyond: Throughout the metagenomic analysis, the authors discuss the prevalence of Enterobacteriaceae in fecal metagenomes but rarely address their relative abundance. In particular, it would be helpful to compare the Enterobacteriaceae/Prevotellaceae (E/P) ratio in fecal samples to that of the synthetic community. This ratio appears to be substantially lower in real-life fecal samples. Indeed, by our estimates the frequency of samples where the E/P ratio is above 0.1 is ~ 16% in AGP data set (430 samples) and 22% in Indian data set (11 samples), while in the overwhelming majority of samples this ratio is <0.01. This raises a key question: is the in vivo abundance of Enterobacteriaceae sufficient to elicit the same effects and allow S. copri to dominate as observed in the synthetic community, which contains a high level of E. coli (~20% even in mGAM alone, as shown in Fig. 1A, passage 3) as compared to a very low relative abundance of Enterobacteriaceae in AGP data set (median=0.00% and mean=0.42%) and even in Indian dataset (median=0.28% and mean=2.63%).

Line 324-325: "…positive correlation between the relative abundance of Prevotellaceae and the number of Enterobacteriaceae species". Likewise, while talking about correlations between Enterobacteriaceae and Prevotellaceae, the authors point to the "number of Enterobacteriaceae species" and not to their relative abundance. Indeed, by our estimates there is absolutely no meaningful correlation between these bacterial families at the level of relative abundances in none of the analyzed datasets (including India, China and Japan-Africa-Peru-USA international dataset). Such a disclaimer should be made to avoid misinterpretation. At the same time, the significance of the observed correlation at the level of Enterobacteriaceae richness (as opposed to the correlation on the abundance level) is unclear, and it should have been discussed.

Line 359 and beyond: "Discussion". It would be interesting to discuss the new findings in the context of succession of microbial colonizers in the infant gut in non-Westernized populations. Thus, according to some publications [PMID: 38503977; 38093016] Enterobacteriaceae are usually among the first colonizers that are succeeded by Bifidobacterium, and S. copri .

Figure 2E. It is unclear what is the meaning of "Competitive index" (Segatella/Bacteroidaceae) in "no Bacteroidaceae" and "no Segatella" experiments.

Legend to Figure 2. Line 478-479: "In A and D data is representative of n=2-7 independent experiments". What does it mean: that shown triplicates are all from one representative experiment? Or they represent 3 independent experiments (one from each)? (and how it can be for the cases where there were only 2 independent experiments?) Please clarify.

Legend to Figure 3E. Line 492-497. From this legend alone, it is unclear what type of experimental data is depicted - metabolomics or transcriptomics? On one hand, the sentence ""Hits" indicates the number of significantly enriched metabolites…" implies metabolomic measurements (because from changes in gene expression alone, one cannot say anything about the "enriched metabolites"). On the other hand, the sentence "…"Gene Ratio" denotes the proportion of significantly changed metabolites relative to the total metabolites in the pathway" points to likely transcriptomic measurements (otherwise, why Gene Ratio?). But if the latter was true (which is unlikely based on the text in Lines 226-247), the rest of the sentence talking about "changed metabolites" would be incorrect. Please specify the methodology (read-out) and refine the terminology (metabolites vs genes).

Extended Data Fig. 1D: A general concern is that the authors report only relative growth compared to mGAM, which provides less information and is only meaningful if all strains grew well in mGAM to begin with, which is impossible to tell unless raw OD_{600} values are provided (as a supplementary table).
More specifically, it is somewhat surprising that all members of the consortium (including E. coli) are capable to utilize (individually grow on) so many carbohydrate polymers, while not all of them possess the appropriate polymer utilization machinery. (It appears that for E. coli a relatively weak growth, ~30%, is shown even on arabinan?). Some comments are in order, for example, is it possible that their actual growth is due to the presence of short oligo- and monosaccharides in these polymer samples?
As just one example, the following growth results reported for B. adolescentis and B. longum subsp. Longum (assuming it is subsp. Longum and not Infantis or Suiss) raise questions:
- Both species grew on chitin and gellan. Gellan is a tetrasaccharide polymer composed of D-glucose, L-rhamnose, and D-glucuronic acid, none of which these species are known to metabolize in this polymeric form

- Similarly, the growth of B. longum subsp. longum on pachyman, nigeran, and 1,4-β-D-mannan is somewhat unexpected. Notably, there was no growth on gluco- and galactomannan, while there was growth on 1,4-β-D-mannan, which is another surprising observation.
- Conversely, it's unexpected that B. adolescentis failed to grow on pullulan, maltodextrin, amylopectin, or starch, as this strain encodes an extracellular amylopullulanase capable of degrading these substrates.

Altogether, such observations raise concerns about substrate purity (potential monosaccharide/oligosaccharide contamination) or possible technical artifacts. Authors may refer to recent publications reporting growth studies and genomics-based reconstruction of carbohydrate utilization that are particularly abundant for various Bifidobacterium species.

Extended Figure 2B and 2D. How can you rationalize these observations?
2B: out of 6 Bacteroidaceae strains only two show a robust individual growth on arabinan: B. theta and P. vulgatus.
2D: however, when grown together (and in the presence of S. copri), the only two with substantial relative abundance (on mGAM or arabinan) are: B. theta and B. fragilis

Reviewer #2

(Remarks to the Author)
Tawk et al. performed a screen to decipher dietary components that favor the growth of S. copri or Bacteroides species. Curiously, these do not predict whether Bacteroides dominates over Prevotella or vice versa. They delve into one case, metabolism of arabinan, determining that the predicted competitive edge is dependent on the presence of certain Enterobacteriaceae. Although the authors extend their initial observations for different E. coli strains, different glycans and different Segatella strains, the results are still light on mechanism. Additionally, the conclusions overall are rather limited in terms of understanding why microbiomes are dominated by these species.

Major comments:

The researchers jump to the conclusion in line 188 that cross-feeding is impacting the outcome– “direct arabinan utilization is necessary for S. copri expansion within the community, it is not sufficient; interactions with other community members are required for S. copri to dominate..”. And surely they find an example of cross-feeding, but it is not clear whether this is the cause for these abundances in complex and natural communities. The authors should demonstrate whether their findings hold up in a complex mixed community with and without E. coli.

The authors do not really address why the presence of E. coli which can metabolize byproducts of arabinan metabolism gives P. copri a boost. How does S. copri benefit from this whereas Bacteroides sp. do not. This seems like a central question that is not addressed in the manuscript. Does supplementation with the simple sugars (arabinose, galactose, rhamnose, galacturonic acid) change the outcomes of the competition experiments? The papers the authors cite as examples of cross-feeding in other species go deeper into the mechanism by which the mutualism comes about. They also demonstrate the phenomenon in mouse models.

Similarly, the authors do not demonstrate that the S. copri advantage is dependent on the arabinan byproduct utilization genes in E. coli, which could be tested by knocking out these genes in E. coli. Also, do all of the strains tested in Figure 3C have these genes? Have the authors tested to see whether cross-feeding is actually occurring in these samples by analyzing the fiber degradation and its byproduct usage?

The authors find contrary results across Segatella strains. It seems like they do not all obtain the same benefit from E. coli. Could the authors decipher any mechanistic insight from this result?

The manuscript concludes with a fairly meager metagenomic analysis of microbiomes. The authors did not examine the presence of the genes they deem important in these microbiomes, there is the issue of compositional effects discussed above, and the issue of Segatella strain-level variation in its response to E. coli.

Importantly, the authors find that, overall, the capacity to use different glycans did not predict the direction of Prevoteallaceae/Bacteroides ratios in the community (line 138). What is the main mechanism here? The authors do not consider other effects on composition—immune pressure or priority effects, for example. A mouse model would help assess what might be going on here as well (see Gellman et al. Cell Reports 2023). Without this, deeper metagenomic analysis, some idea of mechanism and demonstration that S. copri gets a boost with E. coli in a much more complex mock community, it’s hard to ascertain the applicability of their conclusions.

Analytical comments:

The correlation analysis of the abundances of organisms in 16S is not as simple as the competitive index the authors use. There are compositional effects that have been well documented in the literature and are customarily used in 16S rRNA analysis to study correlation. SPARCC is an old and commonly used method. Others include SPIEC-EASI and SECOM, for example. Additionally, the authors may have true abundance data they can use. This is important as just comparing percent abundances after some perturbation may lead to spurious results.

The food analysis may be a red herring. The authors imply that individuals obtain E. coli or other Enterobacteriaceae strains from the food they eat. These conclusions seem disjointed from the rest of the manuscript. Analysis of transmission requires strain-level resolution for conclusive evidence. It could very well be that the strains found in food products are not those that are found in the gut microbiome. Most data suggests that bacteria from food are incapable of colonizing or even surviving in the gut and therefore appear as transient members of the microbiome.

Minor comments:

The data in Figure 3C is presented nonintuitively. Why not show the growth curves? Given the variability, it is surprising this was only done in duplicate.

Arabinan is a common glycan found in many fruits and grains. How do the authors conceptualize these findings given that arabinan is commonly available globally.

Reviewer #3

(Remarks to the Author)
The manuscript by Tawk et al. describes a series of experiments that reveal ecological dynamics of gut microbes in response to different dietary substrates, particularly Segatella copri, E.coli, and arabinan. I commend the authors on designing and executing a robust set of experiments, and for a well-written and compelling presentation of their work. The findings advance understanding of the interplay between diet and microbiota and, as such, are valuable to the field and can inspire future avenues of research.

The following are specific comments that the authors should address:

1. I wonder if the title could be a bit more specific. The authors focus mainly on E. coli in the results, whereas Enterobacteriaceae as a family seems to only be presented in the last section on comparing Westernized vs Non-Westernized populations (which I also have a comment on, see #18). Also, instead of 'dominant anaerobes', perhaps specify the competition between Bacteroidaceae and Prevotellaceae?

2. Line 30: I suggest using the terms 'industrialized' and 'non-industrialized' vs. words like 'modern' and 'Westernized' throughout the abstract and main text. I would argue all populations currently on Earth are 'modern' (in the sense of time, not level of advancement). With industrialization becoming more and more widespread to countries beyond those in North America and Europe, 'Western' isn't an appropriate term anymore, either.

3. Line 60: if 'Westernized' is changed to 'industrialized', then 'industrialization' can be removed later on in the sentence.

4. Line 73: CAZymes does not need to be defined again (already defined in line 71).

5. Figure 1: suggest using a different colour for 'Synthetic', as the shade of green is a bit hard to distinguish from that of 'Bacterial'. Perhaps a light purple?

6. Lines 101-103: the vast majority of diet-derived vitamins are absorbed in the small intestine and would not be available to microbiota in the large intestine. I assume, here, you mean those vitamins that are synthesized by bacteria? If yes, I suggest making this clearer, as diet-derived vitamins likely do not significantly influence the microbiota. Also related to this, in Line 106, what exactly are you referring to as 'physiological ranges' of the vitamins? In the human gut?

7. Line 117: suggest listing some examples of what 'gelling compounds' you are referring to here.

8. Figure 1D: I was surprised that the authors do not mention E. coli in this paragraph when Figure 1D is referenced (starting at line 113), even though it is clear that E. coli increased in response to almost as many compounds as S. copri. What was the reasoning for not highlighting E. coli here? Also, it seems like the number of compounds that increased the abundance of E. coli does not match the number listed in Figure 1F. The magnitude seems to be quite high and similar to that of S. copri, and yet E. coli significantly increased less than a third of what S. copri did? All in all, I think it is worthwhile mentioning E. coli much earlier on in the manuscript, especially in reference to Figure 1D and Figure 2A/D. I believe the first mention of E. coli is not until line 202 and Figure 3A/B.

9. Line 137: is Figure 1F the correct figure to reference here? It's not clear to me how this bar graph shows that most glycans promote S. copri growth at the expense of Bacteroidaceae.

10. Figure 3: Overall, I commend the authors for conducting such systematic experiments, and for presenting the results in a very easy-to-understand manner! For Figure 3C, can the Calculated AUC legends be altered so that they are all the same range? This would allow for easier comparisons among the three heatmaps.

11. Extended Data Fig. 3: is there a need to list A/B/C when there's no specific mention of the different panels in the manuscript?

12. Lines 240-243: suggest revising the sentence (for clarity) that describes the pathways contributed by E. coli. It took me a few read-throughs to understand its meaning.

13. Lines 265-269: do the authors have data to support at what timepoint/hour these changes occurred? If so, it would be interesting to include (similar to how the authors noted that genes in PUL14 were upregulated at 8h, line 258).

14. Figure 4B: can the authors comment on Segatella brasiliensis HDD05 (clade D), which appears to be the only one that grows without E. coli? It would be worthwhile to comment on this in the manuscript, and briefly speculate why.

15. Did the authors test pathogenic strains of E. coli (e.g., E. coli O157:H7)? Most individuals in the nutrition field, and the general public for that matter, would probably associate E. coli with illness (without considering strain-specificity or context dependency), and might question if E. coli promoting S. copri through specific dietary substrates would be potentially harmful for the host. If this was not tested, I wonder if it should be. If it was tested but not included in the results, I would be interested to know why and what the potential implications are for the host.

16. Have the authors looked at data from PMID: 26552345, and whether responders who had enrichment in P. copri may have also harboured E. coli? This comparison might serve as further validation for the current manuscript's findings, in addition to the analysis of industrialized vs non-industrialized populations.

17. Line 324: please provide correlation coefficients and p values for significant correlation results.

18. Line 326 and Figures/Extended Data Figures 5: what is the justification for looking at Enterobacteriaceae and Prevotellaceae as families, and not E. coli and S. copri as species? I would include this justification in the text, or also present correlations between the species in the manuscript, if possible.

19. Lines 353-356: is this simply a reflection of fruits and vegetables being watered when grown, which may be the source of Enterobacteriaceae, and not because of the fibre content of these foods?

20. Discussion: I would like to see the authors describe some of the limitations of their work, and also list a few clear future directions/what experiments could be conducted in humans to validate the findings further. Can a similar approach be applied for other dietary fibres and keystone species or primary/secondary degraders? What do these results mean for host health? Are synbiotic applications a potential future direction to elicit more consistent responses to dietary fibres or probiotic interventions?

Decision Letter:

22nd August 2025

*Please ensure you delete the link to your author homepage in this e-mail if you wish to forward it to your co-authors.

Dear Till,

Thank you for your patience while your manuscript "Synergy between Enterobacteriaceae and dietary components shapes the competition between the dominant anaerobes of the human gut" was under peer-review at Nature Microbiology. It has now been seen by 3 referees, whose expertise and comments you will find at the end of this email. Although they find your work of some potential interest, they have raised a number of concerns that will need to be addressed before we can consider publication of the work in Nature Microbiology.

In particular, you will see that the referees have expressed concerns regarding exaggeration of claims, unclear mechanism, and some technical problems with the methodology used. Thus, we would require mechanistic insight following the guidance of R1 and R2 – assessing the mutants, looking at the strains with anomalous results - as well as using a more complex system like mice. We also suggest improving the metagenomic analysis as highlighted by R2. However, for us to consider a revised version of this manuscript, all the concerns, not just the suggested ones, will need to be addressed. We'd also require a point-by-point response to all the comments made by the referees.

Should further experimental data allow you to address these criticisms, we would be happy to look at a revised manuscript.

We are committed to providing a fair and constructive peer-review process. Please do not hesitate to contact us if there are specific requests from the reviewers that you believe are technically impossible or unlikely to yield a meaningful outcome.

We strongly support public availability of data. Please place the data used in your paper into a public data repository, if one exists, or alternatively, present the data as Source Data or Supplementary Information. If data can only be shared on request, please explain why in your Data Availability Statement, and also in the correspondence with your editor. For some data types, deposition in a public repository is mandatory - more information on our data deposition policies and available repositories can be found at https://www.nature.com/nature-research/editorial-policies/reporting-standards#availability-of-data.

Please include a data availability statement as a separate section after Methods but before references, under the heading "Data Availability". This section should inform readers about the availability of the data used to support the conclusions of your study. This information includes accession codes to public repositories (data banks for protein, DNA or RNA sequences, microarray, proteomics data etc…), references to source data published alongside the paper, unique identifiers such as URLs to data repository entries, or data set DOIs, and any other statement about data availability. At a minimum, you should include the following statement: "The data that support the findings of this study are available from the corresponding author upon request", mentioning any restrictions on availability. If DOIs are provided, we also strongly encourage including these in the Reference list (authors, title, publisher (repository name), identifier, year). For more guidance on how to write this section please see: http://www.nature.com/authors/policies/data/data-availability-statements-data-citations.pdf

If revising your manuscript:

* Include a "Response to referees" document detailing, point-by-point, how you addressed each referee comment. If no action

was taken to address a point, you must provide a compelling argument. This response will be sent back to the referees along with the revised manuscript.

* If you have not done so already we suggest that you begin to revise your manuscript so that it conforms to our Article format instructions at http://www.nature.com/nmicrobiol/info/final-submission. Refer also to any guidelines provided in this letter.

* Include a revised version of any required reporting checklist. It will be available to referees (and, potentially, statisticians) to aid in their evaluation if the manuscript goes back for peer review. A revised checklist is essential for re-review of the paper.

When submitting the revised version of your manuscript, please pay close attention to our href="https://www.nature.com/nature-portfolio/editorial-policies/image-integrity">Digital Image Integrity Guidelines. and to the following points below:

-- that unprocessed scans are clearly labelled and match the gels and western blots presented in figures.
-- that control panels for gels and western blots are appropriately described as loading on sample processing controls
-- all images in the paper are checked for duplication of panels and for splicing of gel lanes.

EXTENDED DATA FIGURES

When re-submitting your manuscript, please ensure that any supplementary figures and tables that are crucial to the manuscript's conclusions are converted into Extended Data figures and tables to increase visibility of these data. Extended Data figures and tables are online-only (present in the online PDF and full-text HTML versions of the paper), peer-reviewed display items that provide essential background to the article but are not included in the main article due to space constraints. A maximum of ten Extended Data display items (figures and tables) is permitted.

Finally, please ensure that you retain unprocessed data and metadata files after publication, ideally archiving data in perpetuity, as these may be requested during the peer review and production process or after publication if any issues arise.

Please use the link below to submit a revised paper:

Link Redacted

Note: This url links to your confidential homepage and associated information about manuscripts you may have submitted or be reviewing for us. If you wish to forward this e-mail to co-authors, please delete this link to your homepage first.

Nature Microbiology is committed to improving transparency in authorship. As part of our efforts in this direction, we are now requesting that all authors identified as 'corresponding author' on published papers create and link their Open Researcher and Contributor Identifier (ORCID) with their account on the Manuscript Tracking System (MTS), prior to acceptance. This applies to primary research papers only. ORCID helps the scientific community achieve unambiguous attribution of all scholarly contributions. You can create and link your ORCID from the home page of the MTS by clicking on 'Modify my Springer Nature account'. For more information please visit please visit www.springernature.com/orcid.

If you wish to submit a suitably revised manuscript we would hope to receive it within 6 months. If you cannot send it within this time, please let us know. We will be happy to consider your revision, even if a similar study has been accepted for publication at Nature Microbiology or published elsewhere (up to a maximum of 6 months).

All the best with the revision!

Best wishes,

****
Reviewer Expertise:

Referee #1: Metabolic interactions, Metagenomics
Referee #2: Microbial interactions, Segatella copri
Referee #3: Diet-microbiome interactions

Reviewer Comments:

Reviewer #1 (Remarks to the Author):

The manuscript by C. Tawk et al. addresses a highly compelling and important question regarding the dietary and ecological determinants of Prevotellaceae versus Bacteroidaceae dominance in the human gut microbiome. Using a synthetic microbial consortium model, the authors present two key findings: (i) Segatella copri markedly outcompetes several Bacteroides and Phoaceicola strains on certain carbohydrate substrates, particularly arabinan (as well as on some B vitamins), whereas the reverse is observed on other carbon sources; and (ii) this competitive dynamic is contingent on the presence of E. coli (or other Enterobacteriaceae members) within the consortium.

Despite the inherent limitations of in vitro co-culture systems, these findings are corroborated by large-scale metagenomic analyses of microbiomes from Westernized (Bacteroidaceae-dominated) and non-Westernized (Prevotellaceae-dominated) populations, as well as microbial metagenomic profiling of different food products from diverse geographic regions.
While the mechanistic basis of these observations remains only partially elucidated, the study is rigorous, impactful, and of broad relevance. I recommend publication following some revisions.

Line 117: "… vitamin C, which exhibited antimicrobial activity…". This is most likely an artifact caused by a substantial drop in pH. The Gifu Anaerobic Medium (GAM) is not strongly buffered, and according to the supplementary tables, vitamin C was added in a form of ascorbic acid rather than sodium ascorbate, which is much more pH-neutral. This may deserve a brief comment to overcome a potentially wrong (at least not sufficiently justified) interpretation (about a genuine antimicrobial activity of ascorbate).

Line 122-123. A strong impact of B-vitamins (B3, B5 and somewhat B6) on relative abundance of S. copri is mysterious and deserves at least some discussion. Indeed, all strains of S. copri are prototrophs for (can synthesize de novo) all B-vitamins except B7 (biotin) and B12 (cobalamin). Moreover, the detailed genomic reconstruction of B vitamin biosynthesis (eg see [PMID: 31275260]) clearly shows that S. copri does not have a machinery to salvage B3 (no pncAB genes, no niacin transporters), and does not have known B5 uptake transporters. Therefore, the effect of B-vitamins on S. copri fitness cannot be a direct one (which could be a subject of direct testing), but it is rather mediated by the community. (Notably neither B7 nor B12 have any growth-promotion effect on S. copri suggesting that they are present in sufficient quantities in the media). At the same time, while E.coli is also prototrophic for all B-vitamins (except B12), it can potentially benefit from the addition of B-vitamins as it has a metabolic machinery to salvage both B3 and B5 from the medium. While the presence of B3 and B5 did not affect the abundance of E. coli, it could potentially boost some of its (yet unknown) metabolic pathways that have a hypothesized stimulatory effect on S. copri.

Line 138-149. "… the capacity of the different species to utilize the glycans did not predict the direction of Prevotellaceae/Bacteroidaceae ratios within the community". This statement might be too extreme and worth softening by saying something like "…did not always predict the direction…". Indeed, it is supported by two examples of substrates utilizable by both Segatella and Bacteroidaceae strains. In both examples (pectic galactan and arabinan), their relative abundance would be driven by quantitative factors (efficiency of utilization, intrinsic growth rate), and it certainly cannot be predicted by a simple qualitative reasoning (even in the absence of community cross-talk). In that regard, it would be useful to consider an alternative case of a substrate(s), which is preferentially utilized by Bacteroidaceae strains and not Segatella. For example, pullulan (as far as could be deduced from the Extended Data Figure 1D) seems to fall in this category? Likewise, the statement: "These experiments demonstrate that assessing the utilization capacity of dietary components by individual commensal species in monoculture does not reliably predict the overall community dynamics. Other factors of bacterial cross-talk…" (Lines 144-146) does not seem fully justified (even if correct per se) in this context, because it is based on qualitative (growth/no growth in isolation) rather than quantitative (growth rates-based) reasoning.

Line 273. "… observed a downregulation of tryptophanase genes, including tnaA…". Why plural? There is only one tryptophanase in E.coli, and it is encoded by a single gene tnaA.

Line 278-280 (and beyond). While the cross-feeding of monosaccharides, products of extracellular hydrolysis of arabinan by Bacteroidales, was clearly demonstrated and mechanistically rationalized as beneficial for E. coli, the mechanisms that "leads to the expansion of S. copri" remain elusive. It should be directly admitted, and it would be beneficial to propose and discuss some hypotheses (possibly in Discussion). Minimally, one can hypothesize that a significant metabolic shift observed in E. coli upon cross-feeding arabinan-originating monosaccharides leads to excretion of (yet unknown) metabolite(s) that selectively promotes Segatella (and/or suppress Bacteroides?). A role of this hypothetical metabolite would be rather in signaling than in nutritional cross-feeding. However, the identification of its nature would require dedicated studies (like metabolomic analysis of media (as opposed to whole-cells), fractionation/testing the growth-stimulatory effect of fractions, screening of E. coli KO library, etc). None of it is requested of authors except for some candid discussion. (On the other hand, what if tnaA downregulation is a hint, which, for example, would lead to increased levels of tryptophan, which may cross-feed to Segatella and impact its growth rate if endogenous tryptophan synthesis is not efficient enough?)

Line 420: "Our results show that similarly S. copri engages in mutualistic cross-feeding with E. coli" As already emphasized above, these data are not sufficient to claim "mutualistic cross-feeing". While the data suggest that S. copri likely provides monosaccharides that support E. coli growth (and Bacteroides apparently do the same), it is not shown which metabolites (if any) E. coli provides in return. Therefore, without additional evidence, the observed phenomena may be at best referred to as "possible (hypothesized) mutualistic cross-feeding".

Lines 593-602: section "Growth on sole carbon sources" lacks some essential details. For example, the composition of "minimal media " was not specified. Was it the same for all species? Unlikely so, unless it at least included a number of B vitamins, and it is essential to know which ones and in what quantities?

Line 322 and beyond: Throughout the metagenomic analysis, the authors discuss the prevalence of Enterobacteriaceae in fecal metagenomes but rarely address their relative abundance. In particular, it would be helpful to compare the Enterobacteriaceae/Prevotellaceae (E/P) ratio in fecal samples to that of the synthetic community. This ratio appears to be substantially lower in real-life fecal samples. Indeed, by our estimates the frequency of samples where the E/P ratio is above 0.1 is ~ 16% in AGP data set (430 samples) and 22% in Indian data set (11 samples), while in the overwhelming majority of samples this ratio is <0.01. This raises a key question: is the in vivo abundance of Enterobacteriaceae sufficient to elicit the same effects and allow S. copri to dominate as observed in the synthetic community, which contains a high level of E. coli (~20% even in mGAM alone, as shown in Fig. 1A, passage 3) as compared to a very low relative abundance of Enterobacteriaceae in AGP data set (median=0.00% and mean=0.42%) and even in Indian dataset (median=0.28% and mean=2.63%).

Line 324-325: "…positive correlation between the relative abundance of Prevotellaceae and the number of Enterobacteriaceae species". Likewise, while talking about correlations between Enterobacteriaceae and Prevotellaceae, the authors point to the "number of Enterobacteriaceae species" and not to their relative abundance. Indeed, by our estimates there is absolutely no meaningful correlation between these bacterial families at the level of relative abundances in none of the analyzed datasets (including India, China and Japan-Africa-Peru-USA international dataset). Such a disclaimer should be made to avoid misinterpretation. At the same time, the significance of the observed correlation at the level of Enterobacteriaceae richness (as opposed to the correlation on the abundance level) is unclear, and it should have been discussed.

Line 359 and beyond: "Discussion". It would be interesting to discuss the new findings in the context of succession of microbial colonizers in the infant gut in non-Westernized populations. Thus, according to some publications [PMID: 38503977; 38093016] Enterobacteriaceae are usually among the first colonizers that are succeeded by Bifidobacterium, and S. copri .

Figure 2E. It is unclear what is the meaning of "Competitive index" (Segatella/Bacteroidaceae) in "no Bacteroidaceae" and "no Segatella" experiments.

Legend to Figure 2. Line 478-479: "In A and D data is representative of n=2-7 independent experiments". What does it mean: that shown triplicates are all from one representative experiment? Or they represent 3 independent experiments (one from each)? (and how it can be for the cases where there were only 2 independent experiments?) Please clarify.

Legend to Figure 3E. Line 492-497. From this legend alone, it is unclear what type of experimental data is depicted - metabolomics or transcriptomics? On one hand, the sentence ""Hits" indicates the number of significantly enriched metabolites…" implies metabolomic measurements (because from changes in gene expression alone, one cannot say anything about the "enriched metabolites"). On the other hand, the sentence "…"Gene Ratio" denotes the proportion of significantly changed metabolites relative to the total metabolites in the pathway" points to likely transcriptomic measurements (otherwise, why Gene Ratio?). But if the latter was true (which is unlikely based on the text in Lines 226-247), the rest of the sentence talking about "changed metabolites" would be incorrect. Please specify the methodology (read-out) and refine the terminology (metabolites vs genes).

Extended Data Fig. 1D: A general concern is that the authors report only relative growth compared to mGAM, which provides less information and is only meaningful if all strains grew well in mGAM to begin with, which is impossible to tell unless raw OD_{600} values are provided (as a supplementary table).
More specifically, it is somewhat surprising that all members of the consortium (including E. coli) are capable to utilize (individually grow on) so many carbohydrate polymers, while not all of them possess the appropriate polymer utilization machinery. (It appears that for E. coli a relatively weak growth, ~30%, is shown even on arabinan?). Some comments are in order, for example, is it possible that their actual growth is due to the presence of short oligo- and monosaccharides in these polymer samples?
As just one example, the following growth results reported for B. adolescentis and B. longum subsp. Longum (assuming it is subsp. Longum and not Infantis or Suiss) raise questions:
- Both species grew on chitin and gellan. Gellan is a tetrasaccharide polymer composed of D-glucose, L-rhamnose, and D-glucuronic acid, none of which these species are known to metabolize in this polymeric form
- Similarly, the growth of B. longum subsp. longum on pachyman, nigeran, and 1,4-β-D-mannan is somewhat unexpected. Notably, there was no growth on gluco- and galactomannan, while there was growth on 1,4-β-D-mannan, which is another surprising observation.
- Conversely, it's unexpected that B. adolescentis failed to grow on pullulan, maltodextrin, amylopectin, or starch, as this strain encodes an extracellular amylopullulanase capable of degrading these substrates.
Altogether, such observations raise concerns about substrate purity (potential monosaccharide/oligosaccharide contamination) or possible technical artifacts. Authors may refer to recent publications reporting growth studies and genomics-based reconstruction of carbohydrate utilization that are particularly abundant for various Bifidobacterium species.

Extended Figure 2B and 2D. How can you rationalize these observations?
2B: out of 6 Bacteroidaceae strains only two show a robust individual growth on arabinan: B. theta and P. vulgatus.
2D: however, when grown together (and in the presence of S. copri), the only two with substantial relative abundance (on mGAM or arabinan) are: B. theta and B. fragilis

Reviewer #2 (Remarks to the Author):

Tawk et al. performed a screen to decipher dietary components that favor the growth of S. copri or Bacteroides species. Curiously, these do not predict whether Bacteroides dominates over Prevotella or vice versa. They delve into one case, metabolism of arabinan, determining that the predicted competitive edge is dependent on the presence of certain Enterobacteriaceae. Although the authors extend their initial observations for different E. coli strains, different glycans and different Segatella strains, the results are still light on mechanism. Additionally, the conclusions overall are rather limited in terms of understanding why microbiomes are dominated by these species.

Major comments:

The researchers jump to the conclusion in line 188 that cross-feeding is impacting the outcome– "direct arabinan utilization is necessary for S. copri expansion within the community, it is not sufficient; interactions with other community members are required for S. copri to dominate..". And surely they find an example of cross-feeding, but it is not clear whether this is the cause for these abundances in complex and natural communities. The authors should demonstrate whether their findings hold up in a complex mixed community with and without E. coli.

The authors do not really address why the presence of E. coli which can metabolize byproducts of arabinan metabolism gives P. copri a boost. How does S. copri benefit from this whereas Bacteroides sp. do not. This seems like a central question that is not addressed in the manuscript. Does supplementation with the simple sugars (arabinose, galactose, rhamnose, galacturonic acid) change the outcomes of the competition experiments? The papers the authors cite as examples of cross-feeding in other species go deeper into the mechanism by which the mutualism comes about. They also demonstrate the phenomenon in mouse models.

Similarly, the authors do not demonstrate that the S. copri advantage is dependent on the arabinan byproduct utilization genes in E. coli, which could be tested by knocking out these genes in E. coli. Also, do all of the strains tested in Figure 3C have these genes? Have the authors tested to see whether cross-feeding is actually occurring in these samples by analyzing the fiber degradation and its byproduct usage?

The authors find contrary results across Segatella strains. It seems like they do not all obtain the same benefit from E. coli. Could the authors decipher any mechanistic insight from this result?

The manuscript concludes with a fairly meager metagenomic analysis of microbiomes. The authors did not examine the presence of the genes they deem important in these microbiomes, there is the issue of compositional effects discussed above, and the issue of Segatella strain-level variation in its response to E. coli.

Importantly, the authors find that, overall, the capacity to use different glycans did not predict the direction of Prevoteallaceae/Bacteroides ratios in the community (line 138). What is the main mechanism here? The authors do not consider other effects on composition—immune pressure or priority effects, for example. A mouse model would help assess what might be going on here as well (see Gellman et al. Cell Reports 2023). Without this, deeper metagenomic analysis, some idea of mechanism and demonstration that S. copri gets a boost with E. coli in a much more complex mock community, it's hard to ascertain the applicability of their conclusions.

Analytical comments:

The correlation analysis of the abundances of organisms in 16S is not as simple as the competitive index the authors use. There are compositional effects that have been well documented in the literature and are customarily used in 16S rRNA analysis to study correlation. SPARCC is an old and commonly used method. Others include SPIEC-EASI and SECOM, for example. Additionally, the authors may have true abundance data they can use. This is important as just comparing percent abundances after some perturbation may lead to spurious results.

The food analysis may be a red herring. The authors imply that individuals obtain E. coli or other Enterobacteriaceae strains from the food they eat. These conclusions seem disjointed from the rest of the manuscript. Analysis of transmission requires strain-level resolution for conclusive evidence. It could very well be that the strains found in food products are not those that are found in the gut microbiome. Most data suggests that bacteria from food are incapable of colonizing or even surviving in the gut and therefore appear as transient members of the microbiome.

Minor comments:

The data in Figure 3C is presented nonintuitively. Why not show the growth curves? Given the variability, it is surprising this was only done in duplicate.

Arabinan is a common glycan found in many fruits and grains. How do the authors conceptualize these findings given that arabinan is commonly available globally.

Reviewer #3 (Remarks to the Author):

The manuscript by Tawk et al. describes a series of experiments that reveal ecological dynamics of gut microbes in response to different dietary substrates, particularly Segatella copri, E.coli, and arabinan. I commend the authors on designing and executing a robust set of experiments, and for a well-written and compelling presentation of their work. The findings advance understanding of the interplay between diet and microbiota and, as such, are valuable to the field and can inspire future avenues of research.

The following are specific comments that the authors should address:

1. I wonder if the title could be a bit more specific. The authors focus mainly on E. coli in the results, whereas Enterobacteriaceae as a family seems to only be presented in the last section on comparing Westernized vs Non-Westernized populations (which I also have a comment on, see #18). Also, instead of 'dominant anaerobes', perhaps specify the competition between Bacteroidaceae and Prevotellaceae?

2. Line 30: I suggest using the terms 'industrialized' and 'non-industrialized' vs. words like 'modern' and 'Westernized' throughout the abstract and main text. I would argue all populations currently on Earth are 'modern' (in the sense of time, not level of advancement). With industrialization becoming more and more widespread to countries beyond those in North America and Europe, 'Western' isn't an appropriate term anymore, either.

3. Line 60: if ‘Westernized’ is changed to ‘industrialized’, then ‘industrialization’ can be removed later on in the sentence.

4. Line 73: CAZymes does not need to be defined again (already defined in line 71).

5. Figure 1: suggest using a different colour for 'Synthetic', as the shade of green is a bit hard to distinguish from that of 'Bacterial'. Perhaps a light purple?

6. Lines 101-103: the vast majority of diet-derived vitamins are absorbed in the small intestine and would not be available to microbiota in the large intestine. I assume, here, you mean those vitamins that are synthesized by bacteria? If yes, I suggest making this clearer, as diet-derived vitamins likely do not significantly influence the microbiota. Also related to this, in Line 106, what exactly are you referring to as ‘physiological ranges’ of the vitamins? In the human gut?

7. Line 117: suggest listing some examples of what ‘gelling compounds’ you are referring to here.

8. Figure 1D: I was surprised that the authors do not mention E. coli in this paragraph when Figure 1D is referenced (starting at line 113), even though it is clear that E. coli increased in response to almost as many compounds as S. copri. What was the reasoning for not highlighting E. coli here? Also, it seems like the number of compounds that increased the abundance of E. coli does not match the number listed in Figure 1F. The magnitude seems to be quite high and similar to that of S. copri, and yet E. coli significantly increased less than a third of what S. copri did? All in all, I think it is worthwhile mentioning E. coli much earlier on in the manuscript, especially in reference to Figure 1D and Figure 2A/D. I believe the first mention of E. coli is not until line 202 and Figure 3A/B.

9. Line 137: is Figure 1F the correct figure to reference here? It’s not clear to me how this bar graph shows that most glycans promote S. copri growth at the expense of Bacteroidaceae.

10. Figure 3: Overall, I commend the authors for conducting such systematic experiments, and for presenting the results in a very easy-to-understand manner! For Figure 3C, can the Calculated AUC legends be altered so that they are all the same range? This would allow for easier comparisons among the three heatmaps.

11. Extended Data Fig. 3: is there a need to list A/B/C when there’s no specific mention of the different panels in the manuscript?

12. Lines 240-243: suggest revising the sentence (for clarity) that describes the pathways contributed by E. coli. It took me a few read-throughs to understand its meaning.

13. Lines 265-269: do the authors have data to support at what timepoint/hour these changes occurred? If so, it would be interesting to include (similar to how the authors noted that genes in PUL14 were upregulated at 8h, line 258).

14. Figure 4B: can the authors comment on Segatella brasiliensis HDD05 (clade D), which appears to be the only one that grows without E. coli? It would be worthwhile to comment on this in the manuscript, and briefly speculate why.

15. Did the authors test pathogenic strains of E. coli (e.g., E. coli O157:H7)? Most individuals in the nutrition field, and the general public for that matter, would probably associate E. coli with illness (without considering strain-specificity or context dependency), and might question if E. coli promoting S. copri through specific dietary substrates would be potentially harmful for the host. If this was not tested, I wonder if it should be. If it was tested but not included in the results, I would be interested to know why and what the potential implications are for the host.

16. Have the authors looked at data from PMID: 26552345, and whether responders who had enrichment in P. copri may have also harboured E. coli? This comparison might serve as further validation for the current manuscript’s findings, in addition to the analysis of industrialized vs non-industrialized populations.

17. Line 324: please provide correlation coefficients and p values for significant correlation results.

18. Line 326 and Figures/Extended Data Figures 5: what is the justification for looking at Enterobacteriaceae and Prevotellaceae as families, and not E. coli and S. copri as species? I would include this justification in the text, or also present correlations between the species in the manuscript, if possible.

19. Lines 353-356: is this simply a reflection of fruits and vegetables being watered when grown, which may be the source of Enterobacteriaceae, and not because of the fibre content of these foods?

20. Discussion: I would like to see the authors describe some of the limitations of their work, and also list a few clear future directions/what experiments could be conducted in humans to validate the findings further. Can a similar approach be applied for other dietary fibres and keystone species or primary/secondary degraders? What do these results mean for host health? Are synbiotic applications a potential future direction to elicit more consistent responses to dietary fibres or probiotic interventions?

****

Version 1:

Reviewer comments:

Reviewer #1

(Remarks to the Author)
Authors did a remarkable job on responding to critical comments and questions of the original review including multiple additional experiments that helped to clarify most important issues. All responses are thorough and detailed, and the edits/additions to the MS are adequate and helpful. As a result tha MS was significantly improved and is recommended for publication in its current form. Adequate statistical tests were applied and duly reflected in the illustrations.

Reviewer #2

(Remarks to the Author)
I thank the authors for putting in a significant amount of additional experiments, as requested. Unfortunately, several of these key experiments now raise new questions rather than support their original claims. They show that Segatella expansion occurs with arabinose and without E. coli. Their mouse experiments Extended Data Fig. 5 were not done without Nissle. Importantly, their knockout of the arabinose utilization genes also does not support the cross-feeding hypothesis. The authors appropriately changed their conclusions to “sugar-dependent signaling”, but it’s not clear what this mechanism is or its relevance. I do not suggest any additional experiments since that seems beyond the scope of this manuscript.

Reviewer #3

(Remarks to the Author)
I thank the authors for their thorough responses to my previous comments, and for conducting follow-up experiments and revising the manuscript accordingly. I have no further comments or suggested edits on the revised version.

Decision Letter:

11th May 2026

Dear Till,

Thank you for your patience while your manuscript "Synergy between Enterobacteriaceae and dietary components shapes the competition between the dominant Bacteroidales of the human gut" was under peer review at Nature Microbiology. It has now been seen by our referees, whose expertise and comments you will find at the end of this email. In the light of their advice, we have decided that we cannot offer to publish your manuscript in Nature Microbiology.

From the reports, you will see that while they find your work of some potential interest, there are some outstanding concerns regarding the mechanism underlying the crossfeeding and the animal experiments which are crucial for the central claims of the manuscript. Unfortunately, this criticism is sufficiently important as to preclude publication of your work in Nature Microbiology.

I have discussed your manuscript and the reviewers’ comments with my colleague Javier Martinez Vesga at Nature Communications. He will be happy to evaluate a response that addresses the outstanding concerns from Reviewer #2 and may not require another round of review.

Should you wish to have your paper considered at Nature Communications, please use the transfer link in the footnote once the revisions are completed. Please provide a detailed point-by-point response file to all the reviewer´s comments. In addition, Nature Communications asks that you incorporate any Figure/data given in your response file that support the findings/conclusions into the Main manuscript or Supplementary files, and that you cited these items accordingly in the manuscript text. These changes should be described in your rebuttal (e.g. Figure R1 corresponds to X figure in the main article or supplement). Please also provide a Cover Letter summarizing the changes.

Important, before transferring your manuscript: Nature Communications requests that all omics raw data are deposited into a permanent data repository (e.g. SRA, ENA, NCBI, Metabolights, etc.) and that the accession codes are provided in a Data Availability Statement in the main article file.

If there is anything you would like to discuss before transferring, please don’t hesitate to contact Javier by e-mail (javier.martinez-vesga@nature.com).

To transfer your manuscript please use our manuscript transfer portal. You will not have to re-supply manuscript metadata and files, unless you wish to make modifications. For more information, please see our manuscript transfer FAQ page.

I am sorry that we cannot be more positive on this occasion, but hope that you find the referees' comments helpful when preparing your paper for resubmission elsewhere.

Yours sincerely,

****

Reviewers Comments:

Reviewer #1 (Remarks to the Author):

Authors did a remarkable job on responding to critical comments and questions of the original review including multiple additional experiments that helped to clarify most important issues. All responses are thorough and detailed, and the edits/additions to the MS are adequate and helpful. As a result tha MS was significantly improved and is recommended for publication in its current form. Adequate statistical tests were applied and duly reflected in the illustrations.

Reviewer #2 (Remarks to the Author):

I thank the authors for putting in a significant amount of additional experiments, as requested. Unfortunately, several of these key experiments now raise new questions rather than support their original claims. They show that Segatella expansion occurs with arabinose and without E. coli. Their mouse experiments Extended Data Fig. 5 were not done without Nissle. Importantly, their knockout of the arabinose utilization genes also does not support the cross-feeding hypothesis. The authors appropriately changed their conclusions to "sugar-dependent signaling", but it's not clear what this mechanism is or its relevance. I do not suggest any additional experiments since that seems beyond the scope of this manuscript.

Reviewer #3 (Remarks to the Author):

I thank the authors for their thorough responses to my previous comments, and for conducting follow-up experiments and revising the manuscript accordingly. I have no further comments or suggested edits on the revised version.

****

Version 2:

Decision Letter:

4th June 2026

Dear Till,

Thank you for your letter asking us to reconsider our decision on your Article entitled "Synergy between Enterobacteriaceae and dietary components shapes the competition between the dominant Bacteroidales of the human gut". After careful consideration and a long discussion with the team, we have decided that we will consider a revised version of your manuscript.

Along with your revised manuscript, you should also submit a separate point-by-point response to all of the concerns raised by the referees, in each case describing what changes have been made to the manuscript or, alternatively, if no action has been taken, providing a compelling argument for why that is the case. If we feel that a substantial attempt has been made to address the referees' comments, this response will be sent back to the referees - along with the revised manuscript - so that they can judge whether their concerns have been addressed satisfactorily or otherwise.

Please use the following link to submit your revised manuscript:

Link Redacted

We hope to receive your revised paper within four weeks. If you cannot send it within this time, please let us know so that we can close your file. In this event, we will still be happy to reconsider your paper at a later date so long as nothing similar has been accepted for publication at Nature Microbiology or published elsewhere in the meantime. Should you miss the four-week deadline and your paper is eventually published, the received date will be that of the revised, not the original, version.

I would appreciate it if you could tell me if you think you will be able to submit a revised manuscript, and also the likely timescale.

I look forward to hearing from you soon.

Best wishes,

Version 3:

Reviewer comments:

Reviewer #2

(Remarks to the Author)
Pardon my mistake about the synthetic community experiment. I jumped to the conclusion that those experiments had been done in mice given the implications of the work on colonization determinants, however colonization of mice with Segatella can be tricky.

The authors have addressed other comments.

Decision Letter:

Our ref: NMICROBIOL-25072578C

9th July 2026

Dear Till,

Thank you for your patience while your manuscript "Synergy between Enterobacteriaceae and dietary components shapes the competition between the dominant Bacteroidales of the human gut" was under peer review at Nature Microbiology. It has now been seen by our referees, and in the light of their advice I am delighted to say that we can in principle offer to publish it, pending minor revisions to satisfy the referees' final requests and to comply with our editorial and formatting guidelines.

If the current version of your manuscript is in a PDF format, please email us a copy of the file in an editable format (Microsoft Word or LaTex)-- we can not proceed with PDFs at this stage.

We are now performing detailed checks on your paper and will send you a checklist detailing our editorial and formatting requirements in a couple of weeks. Please do not upload the final materials and make any revisions until you receive this additional information from us.

Thank you again for your interest in Nature Microbiology Please do not hesitate to contact me if you have any questions.

Sincerely,

Reviewer #2 (Remarks to the Author):

Pardon my mistake about the synthetic community experiment. I jumped to the conclusion that those experiments had been done in mice given the implications of the work on colonization determinants, however colonization of mice with Segatella can be tricky.

The authors have addressed other comments.

****

INTERNAL USE ONLY AIP modules required: ["Life Science"]

Version 4:

Decision Letter:

27th August 2026

Dear Till,

I am pleased to accept your Article "Synergy between Enterobacteriaceae and diet mediates competition between dominant Bacteroidales in the human gut" for publication in Nature Microbiology. Thank you for choosing Nature Microbiology for your work and many congratulations.

I am very happy this paper conquered all the ups and downs through its journey, that I saw from a poster at CSHL meeting in 2024 and through a post-revision appeal. Finally, it has come to a successful conclusion. I am glad to have been a part of it as it was also editorially challenging which is great for my own training.

Over the next few weeks, your paper will be copyedited to ensure that it conforms to Nature Microbiology style. We look particularly carefully at the titles of all papers to ensure that they are relatively brief and understandable.

Once your paper is typeset, you will receive an email with a link to choose the appropriate publishing options for your paper and our Author Services team will be in touch regarding any additional information that may be required. Once your paper has been scheduled for online publication, the Nature press office will be in touch to confirm the details.

You may wish to make your media relations office aware of your accepted publication, in case they consider it appropriate to organize some internal or external publicity. Once your paper has been scheduled you will receive an email confirming the publication details. This is normally 3-4 working days in advance of publication. If you need additional notice of the date and time of publication, please let the production team know when you receive the proof of your article to ensure there is sufficient time to coordinate. Further information on our embargo policies can be found here:
https://www.nature.com/authors/policies/embargo.html

After the grant of rights is completed, you will receive a link to your electronic proof via email with a request to make any corrections within 48 hours. If, when you receive your proof, you cannot meet this deadline, please inform us at rjsproduction@springernature.com immediately. You will not receive your proofs until the publishing agreement has been received through our system

Due to the importance of these deadlines, we ask you please us know now whether you will be difficult to contact over the next month. If this is the case, we ask you provide us with the contact information (email, phone and fax) of someone who will be able to check the proofs on your behalf, and who will be available to address any last-minute problems.

Acceptance of your manuscript is conditional on all authors' agreement with our publication policies (see https://www.nature.com/nmicrobiol/editorial-policies). In particular your manuscript must not be published elsewhere.

Authors may need to take specific actions to achieve compliance with funder and institutional open access mandates. If your research is supported by a funder that requires immediate open access (e.g. according to Plan S principles or the NIH public access policy) then you should select the gold OA route, and we will direct you to the compliant route where possible. Because authors warrant under our subscription licensing terms that they haven't committed to licensing any version of their article under a licence inconsistent with the terms of our agreement – including the applicable embargo period – publication under the subscription model isn't suitable for authors whose funders require no embargo.

If you have any questions about our publishing options, costs, Open Access requirements, or our legal forms, please contact ASJournals@springernature.com

An online order form for reprints of your paper is available at https://www.nature.com/reprints/author-reprints.html. All co-authors, authors' institutions and authors' funding agencies can order reprints using the form appropriate to their geographical region.

We welcome the submission of potential cover material (including a short caption of around 40 words) related to your manuscript; suggestions should be sent to Nature Microbiology as electronic files (the image should be 300 dpi at 210 x 297 mm in either TIFF or JPEG format). Please note that such pictures should be selected more for their aesthetic appeal than for their scientific content, and that colour images work better than black and white or grayscale images. Please do not try to design a cover with the Nature Microbiology logo etc., and please do not submit composites of images related to your work. I am sure you will understand that we cannot make any promise as to whether any of your suggestions might be selected for the cover of the journal.

You can now use a single sign-on for all your accounts, view the status of all your manuscript submissions and reviews, access usage statistics for your published articles and download a record of your refereeing activity for the Nature journals.

To assist our authors in disseminating their research to the broader community, our SharedIt initiative provides you with a unique shareable link that will allow anyone (with or without a subscription) to read the published article. Recipients of the link with a subscription will also be able to download and print the PDF.

As soon as your article is published, you will receive an automated email with your shareable link.

Once again, congratulations and thank you for choosing Nature Microbiology for your work. I will look forward to more such cool stories from your group!

With kind regards,

P.S. Click on the following link if you would like to recommend Nature Microbiology to your librarian
http://www.nature.com/subscriptions/recommend.html#forms

** Visit the Springer Nature Editorial and Publishing website at www.springernature.com/editorial-and-publishing-jobs for more information about our career opportunities. If you have any questions please click here.**

Open Access This Peer Review File is licensed under a Creative Commons Attribution 4.0 International License, which permits use, sharing, adaptation, distribution and reproduction in any medium or format, as long as you give appropriate credit to the original author(s) and the source, provide a link to the Creative Commons license, and indicate if changes were made.
In cases where reviewers are anonymous, credit should be given to 'Anonymous Referee' and the source.
The images or other third party material in this Peer Review File are included in the article's Creative Commons license, unless indicated otherwise in a credit line to the material. If material is not included in the article's Creative Commons license and your intended use is not permitted by statutory regulation or exceeds the permitted use, you will need to obtain permission directly from the copyright holder.
To view a copy of this license, visit https://creativecommons.org/licenses/by/4.0/

Reviewer Comments:

We are grateful to all three reviewers for their insightful and expert evaluation of our work. Their constructive and detailed comments have been very helpful in substantially strengthening the manuscript. Specifically, we performed additional experiments in response to further elucidate the mechanism of *S. copri*-*E. coli* interaction and expanded the metagenomic analysis. This included generating and testing *E. coli* mutants, examining additional conditions, and assessing the fiber-dependent *S. copri*-*E. coli* interaction in complex microbiota models using stabilized fecal communities from newly recruited human donors, metagenomically selected donors from an existing cohort, and mouse cecal communities naturally devoid of Enterobacteriaceae. In summary, we believe these additions strengthened the manuscript and addressed the reviewers' concerns.

Reviewer #1 (Remarks to the Author):

The manuscript by C. Tawk et al. addresses a highly compelling and important question regarding the dietary and ecological determinants of Prevotellaceae versus Bacteroidaceae dominance in the human gut microbiome. Using a synthetic microbial consortium model, the authors present two key findings: (i) Segatella copri markedly outcompetes several Bacteroides and Phoaceicola strains on certain carbohydrate substrates, particularly arabinan (as well as on some B vitamins), whereas the reverse is observed on other carbon sources; and (ii) this competitive dynamic is contingent on the presence of E. coli (or other Enterobacteriaceae members) within the consortium.
Despite the inherent limitations of in vitro co-culture systems, these findings are corroborated by large-scale metagenomic analyses of microbiomes from Westernized (Bacteroidaceae-dominated) and non-Westernized (Prevotellaceae-dominated) populations, as well as microbial metagenomic profiling of different food products from diverse geographic regions.
While the mechanistic basis of these observations remains only partially elucidated, the study is rigorous, impactful, and of broad relevance. I recommend publication following some revisions.

1. Line 117: "… vitamin C, which exhibited antimicrobial activity…". This is most likely an artifact caused by a substantial drop in pH. The Gifu Anaerobic Medium (GAM) is not strongly buffered, and according to the supplementary tables, vitamin C was added in a form of ascorbic acid rather than sodium ascorbate, which is much more pH-neutral. This may deserve a brief comment to overcome a potentially wrong (at least not sufficiently justified) interpretation (about a genuine antimicrobial activity of ascorbate).

Following the reviewer's recommendation, we tested sodium ascorbate alongside ascorbic acid, measured the corresponding pH of the supplemented mGAM media, and performed community assembly and stabilization under those conditions. Indeed, the medium supplemented with ascorbic acid was at pH 4.5 at the start and pH 4 after culturing the community for 24 h. The media supplemented with sodium ascorbate was at pH 7.2 at the start and pH 6 after cultivation. As noted by the reviewer, sodium ascorbate did not inhibit community growth and showed a particular enrichment for *Mediterraneibacter gnavus* (former *Ruminococcus gnavus*) (Figure R1, Figure 1D). Whereas ascorbic acid inhibited community growth due to the acidic pH, with only residual growth of *E. coli* observed. We thank the reviewer for this correction and we have updated Figure 1D and the text accordingly to include the new results with sodium ascorbate.

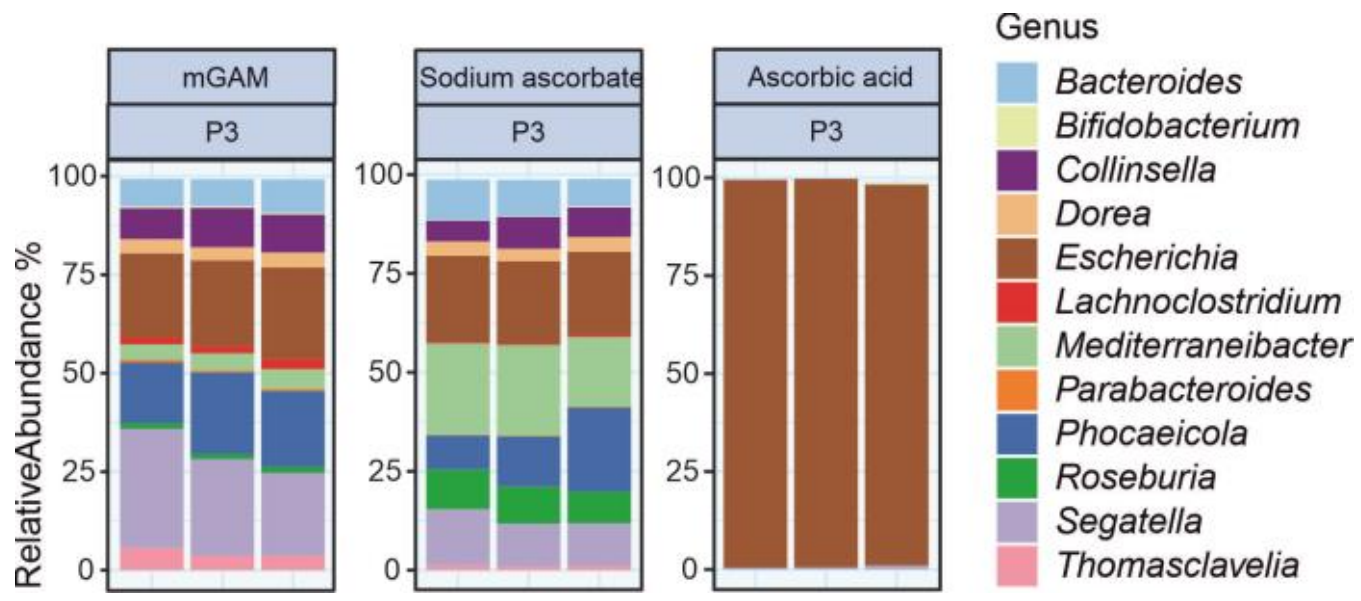

Figure R1. Effect of supplementing sodium ascorbate (vitamin C) on synthetic community assembly. Taxonomic bar plots showing the relative abundance of the top 12 community genera at passage 3 in mGAM, mGAM supplemented with sodium ascorbate or ascorbic acid (10 mg/ml). Data is from 16S rRNA amplicon sequencing with three replicates shown.

2. Line 122-123. A strong impact of B-vitamins (B3, B5 and somewhat B6) on relative abundance of S. copri is mysterious and deserves at least some discussion. Indeed, all strains of S. copri are prototrophs for (can synthesize de novo) all B-vitamins except B7 (biotin) and B12 (cobalamin). Moreover, the detailed genomic reconstruction of B vitamin biosynthesis (eg see [PMID: 31275260]) clearly shows that S. copri does not have a machinery to salvage B3 (no pncAB genes, no niacin transporters), and does not have known B5 uptake transporters. Therefore, the effect of B-vitamins on S. copri fitness cannot be a direct one (which could be a subject of direct testing), but it is rather mediated by the community. (Notably neither B7 nor B12 have any growth-promotion effect on S. copri suggesting that they are present in sufficient quantities in the media). At the same time, while E.coli is also prototrophic for all B-vitamins (except B12), it can potentially benefit from the addition of B-vitamins as it has a metabolic machinery to salvage both B3 and B5 from the medium. While the presence of B3 and B5 did not affect the abundance of E. coli, it could potentially boost some of its (yet unknown) metabolic pathways that have a hypothesized stimulatory effect on S. copri.

As suggested by Reviewer 1, we further investigated the influence of vitamins on the abundance of *S. copri* within the commensal community. We tested whether the B vitamins (B3, B5, and B6) that increase *S. copri* abundance can promote *S. copri* directly when competing with the Bacteroidaceae, or require the presence of *E. coli*. We also tested vitamins B7 and B12 for which *S. copri* is predicted to be an auxotroph. None of these B vitamins provided a competitive benefit to *S. copri* over Bacteroidaceae in the absence of other community members in mGAM, or in the presence of arabinan, suggesting that those vitamins do not confer a direct benefit to *S. copri* (Figure 4B, Figure R2). Strikingly, as predicted by Reviewer 1, vitamin B3 and vitamin B5, were able to promote *S. copri* in an *E. coli*-dependent manner (Figure 4B, Figure R2). This suggests that *E. coli* and *S. copri* could exchange B3- and B5-dependent molecules, leading to *S. copri* expansion. Of note, β-nicotinamide adenine dinucleotide (NAD^+), a direct derivative of vitamin B3, did not promote *S. copri* under any of the tested conditions (Figure R2). Of note, we found in our metatranscriptomics data that *S. copri* significantly upregulates a predicted nicotinamide riboside transporter PnuC (P4D_00002 Log2fold-change=4.54; padj=5.39E-15) along with an upstream outer membrane protein with homology to cobalamin receptors (P4D_00003 Log2Fold-change=4.73; padj=4.03E-38) at 20h in presence of arabinan in the full community (Supplementary Table 3), as well as in the competition with Bacteroidaceae in presence of *E. coli* only. These results suggest that the signaling between *S. copri* and *E. coli* may occur through B vitamins, particularly B3 and B5, in addition to carbohydrates. We added this result on the cross-feeding of B vitamins between *S. copri* and *E. coli* to the revised manuscript (Figure 4).

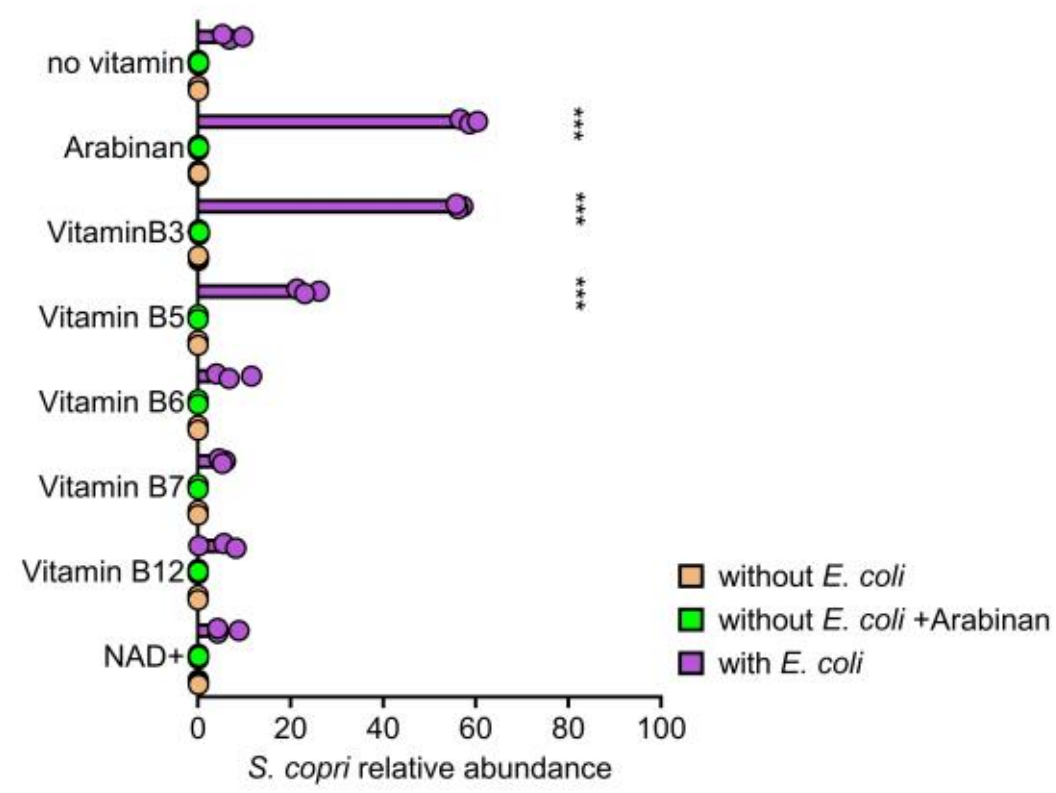

Figure R2. Effect of vitamin supplementation on *S. copri* and Bacteroidaceae competition. Competition between *S. copri* and six Bacteroidaceae in the presence or absence of *E. coli* as in Figure 3A. Vitamins were supplemented in mGAM or mGAM+Arabinan at the concentrations used in Figure 1 (Supplementary Table 2). The relative abundance of *S. copri* in the communities is shown (n=3).

3. Line 138-149. "… the capacity of the different species to utilize the glycans did not predict the direction of Prevotellaceae/Bacteroidaceae ratios within the community". This statement might be too extreme and worth softening by saying something like "…did not always predict the direction…". Indeed, it is supported by two examples of substrates utilizable by both Segatella and Bacteroidaceae strains. In both examples (pectic galactan and arabinan), their relative abundance would be driven by quantitative factors (efficiency of utilization, intrinsic growth rate), and it certainly cannot be predicted by a simple qualitative reasoning (even in the absence of community cross-talk). In that regard, it would be useful to consider an alternative case of a substrate(s), which is preferentially utilized by Bacteroidaceae strains and not Segatella. For example, pullulan (as far as could be deduced from the Extended Data Figure 1D) seems to fall in this category? Likewise, the statement: "These experiments demonstrate that assessing the utilization capacity of dietary components by individual commensal species in monoculture does not reliably predict the overall community dynamics. Other factors of bacterial cross-talk…" (Lines 144-146) does not seem fully justified (even if correct per se) in this context, because it is based on qualitative (growth/no growth in isolation) rather than quantitative (growth rates-based) reasoning.

Following the reviewers' suggestions, we adapted the text. Moreover, we calculated the corresponding growth rates for the examples used using a linear regression at the exponential growth phase for each of the Bacteroidaceae and *S. copri* (R^2>0.98) and calculated the corresponding slope ($\mu = \frac{\Delta \ln(OD600)}{\Delta t}$), to strengthen the argument in the case of these specific examples. We did not include the pullulan example because supplementation with pullulan results in a Bacteroidaceae-dominated community, consistent with the expectation that Bacteroidaceae species can grow on pullulan (except for *B. caccae*) but not *S. copri*. In contrast, we have not found any polysaccharide that can be used exclusively by *S. copri* and by none of the Bacteroidaceae species (>25% of growth). We have revised the text accordingly:

(line 100) "Additionally, the capacity of the different species to utilize the glycans did not always predict the direction of Prevotellaceae/Bacteroidaceae ratios within the community. For instance, pectic galactan is utilized by *B. caccae*, *B. thetaiotaomicron*, *B. ovatus*, *B. uniformis*, *P. vulgatus*, and *S. copri* with calculated growth rates 0.62 h^{-1}, 0.5 h^{-1}, 0.5 h^{-1}, 0.39 h^{-1}, 0.18 h^{-1}, and 0.13 h^{-1}, from highest to lowest, respectively. When pectic galactan is supplemented to the community, it leads to an increase in *S. copri* within the

community and not the Bacteroidaceae species (Fig. 1d, Extended Data Fig. 1d). Further, *B. thetaiotaomicron*, *P. vulgatus,* and *S. copri* all utilize arabinan (at descending growth rates 0.5 h^{-1}, 0.42 h^{-1}, 0.12 h^{-1}), when added, it leads to an expansion of *S. copri* and a depletion of the Bacteroidaceae members (Fig. 1d, Extended Data Fig. 1d). These experiments demonstrate that assessing the utilization capacity of dietary components by individual commensal species in monoculture does not in most cases reliably predict the overall community dynamics. Other factors of bacterial cross-talk, in addition to the utilization capacity of the complex carbohydrate, likely drive community structure and composition."

4. Line 273. "… observed a downregulation of tryptophanase genes, including tnaA…". Why plural? There is only one tryptophanase in E.coli, and it is encoded by a single gene tnaA.

Thank you for the correction. The following correction was made in the text (line 242) "genes in the tryptophanase (tna) operon in *E. coli* were downregulated: tryptophanase leader peptide *tnaC*, low-affinity tryptophan permease *tnaB*, and tryptophanase *tnaA*."

5. Line 278-280 (and beyond). While the cross-feeding of monosaccharides, products of extracellular hydrolysis of arabinan by Bacteroidales, was clearly demonstrated and mechanistically rationalized as beneficial for E. coli, the mechanisms that "leads to the expansion of S. copri" remain elusive. It should be directly admitted, and it would be beneficial to propose and discuss some hypotheses (possibly in Discussion). Minimally, one can hypothesize that a significant metabolic shift observed in E. coli upon cross-feeding arabinan-originating monosaccharides leads to excretion of (yet unknown) metabolite(s) that selectively promotes Segatella (and/or suppress Bacteroides?). A role of this hypothetical metabolite would be rather in signaling than in nutritional cross-feeding. However, the identification of its nature would require dedicated studies (like metabolomic analysis of media (as opposed to whole-cells), fractionation/testing the growth-stimulatory effect of fractions, screening of E. coli KO library, etc). None of it is requested of authors except for some candid discussion. (On the other hand, what if tnaA downregulation is a hint, which, for example, would lead to increased levels of tryptophan, which may cross-feed to Segatella and impact its growth rate if endogenous tryptophan synthesis is not efficient enough?)

We have conducted additional experiments to gain deeper mechanistic insight into the interaction between *E. coli* and *S. copri* within the community. Our data suggest that *E. coli's* influence on community dynamics is unlikely to happen through the inhibition of Bacteroidaceae. In our co-culture transwell assays, no inhibition of Bacteroidaceae growth was observed under any condition (Figure R3A). Both *S. copri* and the Bacteroidaceae benefit from *E. coli* in co-culture, with *S. copri* showing particularly enhanced growth in the presence of arabinan (Figure 3C and Extended Figure 3A-B). Of note, when the Bacteroidaceae are co-cultured with both *E. coli*+*S. copri*, their growth is similar to that observed in the absence of *E. coli* (Figure R3A). This suggests that *E. coli* may provide a shared beneficial factor that *S. copri* and Bacteroidaceae compete for, with a preferred polysaccharide conferring a competitive advantage to *S. copri*.

To identify the molecules underlying these dynamics, we tested tryptophan, as suggested by the reviewer, since arabinan conditions could increase tryptophan levels by downregulating of tryptophanase (*tnaA*) in *E. coli,* according to our metatranscriptomics data. Additionally, metabolomics showed enrichment of the tryptophan metabolism pathway, including tryptophan, in arabinan in the presence of *E. coli* (Extended Figure 3C; Supplementary Table 4). However, supplementation with tryptophan or indole (0.5 g/L) did not confer a competitive advantage to *S. copri*. Moreover, *E. coli* Nissle mutants lacking *tnaA* or the tryptophan biosynthesis gene *trpE* still promoted *S. copri* expansion similarly to wild type (Figure R3B), suggesting tryptophan metabolism is not the driving factor.

To explore mechanisms beyond tryptophan metabolism, we examined other pathways highlighted by our metatranscriptomics and metabolomics data and tested known *S. copri* auxotrophies and essential

growth components. We generated additional *E. coli* Nissle mutants targeting cytochrome c biogenesis ATP-binding export protein *ccmA*, which was strongly upregulated in arabinan, and the serine acetyltransferase *cysE*, required for cysteine biosynthesis, also recently shown to be implicated in cross-feeding between *E. coli* and *Bifidobacteria* (https://doi.org/10.1101/2025.03.24.645027). However, both mutants still promoted *S. copri* expansion similarly to wild type (Figure R3B). Supplementation with cysteine, hemin, or hematin also had no effect (Figure R3C).

We next tested broader nutrient limitations by supplementing amino acids, purines/pyrimidines, trace elements, glutathione, or coenzyme A, but none conferred a competitive advantage to *S. copri*. Likewise, supplementation with succinate (pH 5.0) or lactate did not reproduce the *E. coli*-mediated benefit (Figure R3C).

Overall, across 14 supplementation conditions and four *E. coli* mutants, we could not identify the specific factor(s) responsible, suggesting that pinpointing the mechanism would require more extensive genetic and metabolomic screening beyond the scope of this study, as the reviewer predicted. Nonetheless, as described above, in addition to carbohydrates, vitamin B3 and B5 emerged as potential contributors to the *E. coli*-driven expansion of *S. copri*, suggesting a possible exchange of vitamin-related molecules between these species.

As recommended by the reviewer, we added a section in the text discussing the potential mechanisms involved and explicitly state that at this point the molecular mechanism by which *E. coli* boosts *S. copri* remains elusive. Please see response 6 below for the justification of this statement.

(line 450) "We show that the presence of simple sugars released from polysaccharide degradation, independent of their utilization, is sufficient to drive an *E. coli*-dependent expansion of *S. copri*, indicating a signaling rather than nutritional interaction. This signaling appears to be triggered by diverse nutrients, including vitamins (B3 and B5) and polysaccharide-derived sugars; however, the underlying signaling cascade in *E. coli* that benefits *S. copri* will require further detailed studies."

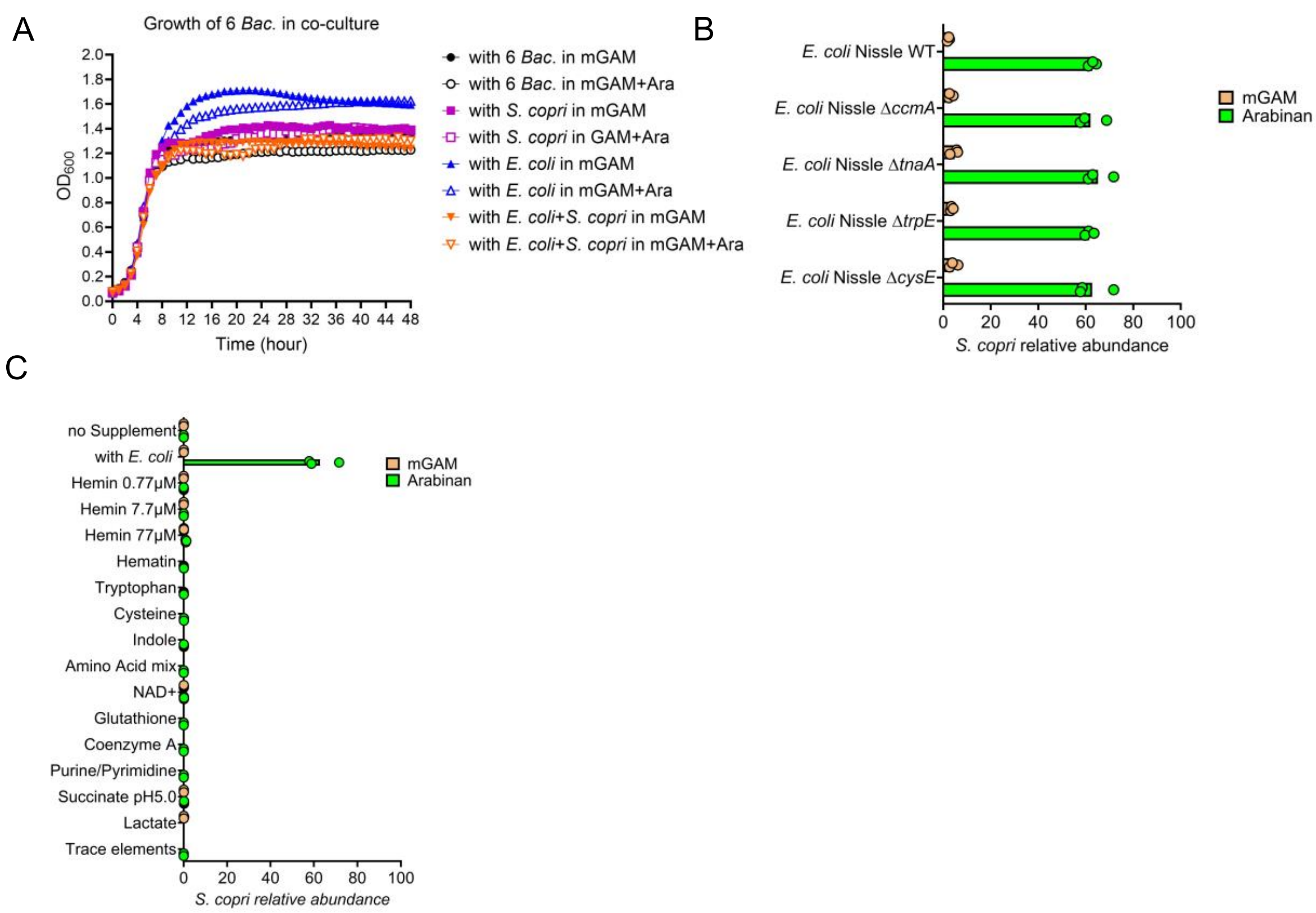

Figure R3. A. Growth curves of the six Bacteroidaceae species grown in co-culture with: six Bacteroidaceae, with *S. copri*, with *E. coli*, and with *E. coli*+*S. copri* in the presence or absence of arabinan. B. Competitions between *S. copri* and six Bacteroidaceae in the presence of different *E. coli* strains. The relative abundance of *S. copri* in the communities is shown (n=3). C. Supplementation of different components into the competition between *S. copri* and six Bacteroidaceae. The relative abundance of *S. copri* in the communities is shown (n=3).

6. Line 420: "Our results show that similarly S. copri engages in mutualistic cross-feeding with E. coli" As already emphasized above, these data are not sufficient to claim "mutualistic cross-feeing". While the data suggest that S. copri likely provides monosaccharides that support E. coli growth (and Bacteroides apparently do the same), it is not shown which metabolites (if any) E. coli provides in return. Therefore, without additional evidence, the observed phenomena may be at best referred to as "possible (hypothesized) mutualistic cross-feeding".

We have changed this statement as shown above and updated it with the additional experimental results we obtained (see response 3 to Reviewer 2). We tested whether *E. coli* utilization of monosaccharides released by *S. copri* during fiber degradation is required for the beneficial effect on *S. copri*. We generated three *E. coli* Nissle mutants deficient in metabolizing monosaccharides derived specifically from arabinan, arabinoxylan, and polygalacturonic acid (Figure 3H; Figure R4). Interestingly, these mutants promoted *S. copri* similarly to wild type in a polysaccharide-dependent manner, indicating that specific sugar utilization is not essential for the promotion of *S. copri* expansion. Furthermore, supplementation with simple sugars directly led to the expansion of *S. copri* in an *E. coli*-dependent manner (Figure 3G), suggesting that sugars act as potential signals that trigger *E. coli's* beneficial effect on *S. copri*.

7. Lines 593-602: section "Growth on sole carbon sources" lacks some essential details. For example, the composition of "minimal media " was not specified. Was it the same for all species? Unlikely so, unless it at least included a number of B vitamins, and it is essential to know which ones and in what quantities?

The minimal media (or defined media) used in this study is based on the published *Bacteroides* Minimal Media (PMID: 18996345) and has previously been shown to support *S. copri* growth by providing all specific essential nutrients (PMID: 34676563). This minimal media is optimized for the Bacteroidales, which are the main focus of this study; all 21 species grew significantly when supplied with their preferred carbon source, suggesting that this media contains all the essential nutrients required for their growth (see OD_{max} in Supplementary Table 8). The full medium composition is now detailed in the Methods section.

8. Line 322 and beyond: Throughout the metagenomic analysis, the authors discuss the prevalence of Enterobacteriaceae in fecal metagenomes but rarely address their relative abundance. In particular, it would be helpful to compare the Enterobacteriaceae/Prevotellaceae (E/P) ratio in fecal samples to that of the synthetic community. This ratio appears to be substantially lower in real-life fecal samples. Indeed, by our estimates the frequency of samples where the E/P ratio is above 0.1 is ~ 16% in AGP data set (430 samples) and 22% in Indian data set (11 samples), while in the overwhelming majority of samples this ratio is <0.01. This raises a key question: is the in vivo abundance of Enterobacteriaceae sufficient to elicit the same effects and allow S. copri to dominate as observed in the synthetic community, which contains a high level of E. coli (~20% even in mGAM alone, as shown in Fig. 1A, passage 3) as compared to a very low relative abundance of Enterobacteriaceae in AGP data set (median=0.00% and mean=0.42%) and even in Indian dataset (median=0.28% and mean=2.63%).

We agree with the reviewer that the relatively high abundance of *E. coli* in our synthetic communities and *in vitro* cultures represents a general limitation of such models. Indeed, the *E. coli*-*S. copri* ratio in the

synthetic community is significantly higher than in fecal samples (mean=0.35±SD 0.017 in arabinan representing the *Segatella*-rich microbiomes) compared to mean=0.105 in Industrialized and mean=0.1 in Non-industrialized fecal samples (Figure 6B-C). In response to Reviewer 2, we also demonstrate the arabinan- and *E. coli*-dependent expansion of *S. copri* in stabilized human and murine fecal and cecal communities (see amended Figure 5 and Reviewer 2 comment 1). Importantly, the Enterobacteriaceae-*S. copri* ratio is significantly lower in the stabilized human fecal communities (mean=0.21±SD 0.12; median=0.19 in the cohort, Figure 5A, and mean=0.21±SD 0.19; median=0.16 in the cohort, Extended Data Figure 5D). Finally, this ratio in the communities from SPF mouse cecum was in the range of the ratios observed in native human fecal microbiomes, where the *E. coli*- and arabinan-dependent expansion of *S. copri* is observed (Figure 5B; mean=0.0016±SD 0.0005 and median=0.0014). Thus, although Enterobacteriaceae occupy a large niche *in vitro*, conditions that more closely approximate fecal E/P ratios still reproduce the *S. copri* expansion phenotype, suggesting that these interactions are robust and likely relevant in the gut despite differences in relative abundance.

9. Line 324-325: "…positive correlation between the relative abundance of Prevotellaceae and the number of Enterobacteriaceae species". Likewise, while talking about correlations between Enterobacteriaceae and Prevotellaceae, the authors point to the "number of Enterobacteriaceae species" and not to their relative abundance. Indeed, by our estimates there is absolutely no meaningful correlation between these bacterial families at the level of relative abundances in none of the analyzed datasets (including India, China and Japan-Africa-Peru-USA international dataset). Such a disclaimer should be made to avoid misinterpretation. At the same time, the significance of the observed correlation at the level of Enterobacteriaceae richness (as opposed to the correlation on the abundance level) is unclear, and it should have been discussed.

Enterobacteriaceae typically occur at low and highly variable relative abundances in human fecal communities, whereas *Segatella copri* is generally much more abundant. This imbalance limits our ability to detect robust correlations between their relative abundances. Moreover, our data do not support a simple linear relationship between Enterobacteriaceae abundance and the capacity to promote *S. copri* within the community, as no consistent *S. copri* abundance range is observed across varying Enterobacteriaceae levels (See response above). We therefore propose that, despite their low abundance, Enterobacteriaceae may exert a marked influence on the abundance of *S. copri* within the community.

Notably, we observe a positive correlation between Enterobacteriaceae species richness and *S. copri* relative abundance in Non-industrialized cohorts (Spearman R=0.18, p=0.00045; Extended Figure 6A), suggesting that the diversity of Enterobacteriaceae within the microbiome may facilitate *S. copri* establishment, potentially through interactions with diverse dietary components. To address this point, we added the following text to the Discussion.

(line 456) "Therefore, the diversity of Enterobacteriaceae species within the microbiome may play an important role in determining *S. copri* establishment and could contribute to creating a broader supportive niche in response to diverse dietary components. Indeed, our human stool metagenomics analysis shows that Enterobacteriaceae prevalence, diversity, and abundance in non-industrialized microbiomes are significantly higher than in industrialized microbiomes. Although we did not observe a correlation between the relative abundance of Enterobacteriaceae and *S. copri*, consistent with previous studies, we did find a significant correlation between the relative abundance of *S. copri* and the number of Enterobacteriaceae species specifically in non-industrialized microbiomes."

10. Line 359 and beyond: "Discussion". It would be interesting to discuss the new findings in the context of succession of microbial colonizers in the infant gut in non-Westernized populations. Thus, according to

some publications [PMID: 38503977; 38093016] Enterobacteriaceae are usually among the first colonizers that are succeeded by Bifidobacterium, and S. copri .

The reviewer's point is indeed of interest. Hence, we explored this question by analyzing a cohort of Industrialized and Non-industrialized infants and children (Extended Figure 6B). In this cohort, Enterobacteriaceae were slightly more abundant as early colonizers in Industrialized children compared with Non-industrialized infants. However, at 24-36 months of age, Enterobacteriaceae became more abundant in Non-industrialized children than in Industrialized children. The presence of Enterobacteriaceae, largely represented by *E. coli*, as early colonizers in children under one year of age appears to be cohort-dependent (PMID: 39809768) and may not be directly linked to the initial establishment of *Segatella* in the gut. It is possible that interactions between Enterobacteriaceae and *S. copri* become more relevant after weaning, when preferred dietary fibers are introduced, as suggested by the data in Extended Figure 6B. Nevertheless, interactions between Enterobacteriaceae and *S. copri* in the developing gut require further investigation to better understand these dynamics during colonization succession in the early gut and post-weaning. To address this point, we added the following text in line 359: "This suggests that as *S. copri* becomes dominant, a prominent niche for Enterobacteriaceae also tends to form. The interaction between these species may become more relevant as dietary fibers preferred by *S. copri* become available."

11. Figure 2E. It is unclear what is the meaning of "Competitive index" (Segatella/Bacteroidaceae) in "no Bacteroidaceae" and "no Segatella" experiments.

The competitive index was calculated as the log ratio of the relative abundance of *S. copri* to the six Bacteroidaceae species to reflect the direction of *S. copri* advantage within the community, consistent with the approach used in Figure 1D. To account for zero values arising from the absence of taxa, zeros were replaced with a small pseudocount (10^{-6}) prior to calculation, according to the following equation:

$$Competitive\ index = Log\ \frac{(S. copri\ relative\ abundance + 0.000001)}{(6\ Bacteroidaceae\ relative\ abundance + 0.000001)}$$

To address potential compositional bias in this analysis (response 7 Reviewer 2), we performed absolute quantification using quantitative PCR with species-specific primers to measure the absolute abundances of these taxa in the communities. Using these measurements, we validated the competitive index between *S. copri* and the Bacteroidaceae across the different communities shown in Figure 2 by applying absolute quantification (Extended Figure 2E). A similarly small pseudocount was applied to the qPCR data to account for cases in which specific taxa were absent. We have added additional details describing these calculations in the Methods section.

12. Legend to Figure 2. Line 478-479: "In A and D data is representative of n=2-7 independent experiments". What does it mean: that shown triplicates are all from one representative experiment? Or they represent 3 independent experiments (one from each)? (and how it can be for the cases where there were only 2 independent experiments?) Please clarify.

We simplified the legend to avoid confusion. We present one representative experiment with three replicates in Figure 2, panels A and D.

13. Legend to Figure 3E. Line 492-497. From this legend alone, it is unclear what type of experimental data is depicted - metabolomics or transcriptomics? On one hand, the sentence ""Hits" indicates the number of significantly enriched metabolites…" implies metabolomic measurements (because from changes in gene expression alone, one cannot say anything about the "enriched metabolites"). On the other hand, the sentence "…"Gene Ratio" denotes the proportion of significantly changed metabolites relative to the total metabolites in the pathway" points to likely transcriptomic measurements (otherwise, why Gene Ratio?).

But if the latter was true (which is unlikely based on the text in Lines 226-247), the rest of the sentence talking about "changed metabolites" would be incorrect. Please specify the methodology (read-out) and refine the terminology (metabolites vs genes).

We agree with the reviewer that terminology used to describe the metabolomics data lacked some clarity. We now refer to metabolites and metabolic pathways clearly in Extended Data Figure 3C.

14. Extended Data Fig. 1D: A general concern is that the authors report only relative growth compared to mGAM, which provides less information and is only meaningful if all strains grew well in mGAM to begin with, which is impossible to tell unless raw OD_{600} values are provided (as a supplementary table). More specifically, it is somewhat surprising that all members of the consortium (including E. coli) are capable to utilize (individually grow on) so many carbohydrate polymers, while not all of them possess the appropriate polymer utilization machinery. (It appears that for E. coli a relatively weak growth, ~30%, is shown even on arabinan?). Some comments are in order, for example, is it possible that their actual growth is due to the presence of short oligo- and monosaccharides in these polymer samples?

We expressed growth as a fraction of growth in mGAM to normalize for the substantial variability in absolute growth yields across species and carbon sources and to enable clearer comparison within a single figure. We acknowledge that mGAM does not represent the optimal growth condition for all species, as evidenced by instances where growth in minimal media exceeds 100% in the presence of preferred polysaccharides. To provide additional context, we report the OD_{max} values for each species grown in mGAM and in each condition in Supplementary Table 8.

Some commercially available polysaccharides are supplied without strict purity guarantees and may contain contaminating oligo- and monosaccharides, which has been a general limitation in the field, as the study of specific polysaccharide utilization in detail sometimes requires customized and laborious extractions and purifications (PMID: 18996345). Consistent with this, we occasionally observe low levels of residual, non-specific growth on certain polysaccharides. However, extensive non-specific growth is generally not observed, and *E. coli* growth serves as a useful indicator of simple sugar contamination (Figure R4B; response 3 to Reviewer 2). *E. coli* displays minor growth in minimal M9 media with arabinan (OD_{max} 0.26), arabinoxylan (OD_{max} 0.18), and polygalacturonic acid (OD_{max} 0.22) compared to OD_{max} 0.9 with glucose or arabinose (Figure R4B). This shows that there is some residual growth due to background simple sugar contamination, but it is minimal compared to the specific growth observed with a preferred carbon source (<30% relative to glucose).

To account for background growth, we averaged the extent of residual growth across species and conditions and defined >25% growth relative to mGAM as a threshold for specific utilization of a polysaccharide. A few substrates such as gellan, pachyman, β-D-glucan, nigeran, HM pectin, kefir gum, and inulin (which shows growth patterns similar to fructooligosaccharides), exhibited robust growth across most species, suggesting higher levels of contaminating simple sugars. Overall, for most polysaccharides, contaminating sugars are unlikely to be a major confounding factor, as they are rapidly depleted and do not support sustained or robust growth.

This screening approach may have certain limitations, including the use of a single medium for all species and reliance on commercially sourced polysaccharides with variable purity. Consequently, certain species-polysaccharide interactions may require additional validation. Nevertheless, for the purposes of this study, the screen provides a useful overview of substrate utilization capacities across community members and highlights cases in which factors beyond substrate availability likely influence community composition, such as the Bacteroidaceae example described below and the specific *S. copri*-*E. coli* interaction characterized here. We added a disclaimer in the Methods to clarify this technical limitation.

As just one example, the following growth results reported for B. adolescentis and B. longum subsp. Longum (assuming it is subsp. Longum and not Infantis or Suiss) raise questions:

- Both species grew on chitin and gellan. Gellan is a tetrasaccharide polymer composed of D-glucose, L-rhamnose, and D-glucuronic acid, none of which these species are known to metabolize in this polymeric form

- Similarly, the growth of B. longum subsp. longum on pachyman, nigeran, and 1,4-β-D-mannan is somewhat unexpected. Notably, there was no growth on gluco- and galactomannan, while there was growth on 1,4-β-D-mannan, which is another surprising observation.

Indeed, as discussed above, these substrates likely contain substantial levels of contaminating simple sugars that can support the growth of multiple species indiscriminately.

- Conversely, it's unexpected that B. adolescentis failed to grow on pullulan, maltodextrin, amylopectin, or starch, as this strain encodes an extracellular amylopullulanase capable of degrading these substrates. Altogether, such observations raise concerns about substrate purity (potential monosaccharide/oligosaccharide contamination) or possible technical artifacts. Authors may refer to recent publications reporting growth studies and genomics-based reconstruction of carbohydrate utilization that are particularly abundant for various Bifidobacterium species.

Bifidobacteria are generally regarded as efficient utilizers of resistant starches and starch granules, and certain *Bifidobacterium adolescentis* strains have been shown to grow on pullulan, maltodextrin, amylopectin, and starch, such as *B. adolescentis* 22L (PMID: 25063659). However, the capacity to utilize these substrates appears to be highly strain-specific, and some *B. adolescentis* strains have been reported to show little or no growth on pullulan, amylopectin, or starch (PMID: 38294252; PMID: 16885278). To our knowledge, there are no published reports specifically characterizing polysaccharide utilization by *B. adolescentis* DSM 20083, the strain used in our study.

Using our minimal medium, we observed robust growth of both *Bifidobacteria* species when preferred substrates were supplied (e.g., *B. adolescentis* DSM20083, OD_{max} 0.9, and *B. longum subs. longum* DSM20219, OD_{max} 0.5 on galactooligosaccharides), indicating that the medium supports their growth. Therefore, in cases where no growth was detected, we infer that the corresponding substrate is not utilized by that strain under the tested conditions.

15. Extended Figure 2B and 2D. How can you rationalize these observations?
2B: out of 6 Bacteroidaceae strains only two show a robust individual growth on arabinan: B. theta and P. vulgatus.
2D: however, when grown together (and in the presence of S. copri), the only two with substantial relative abundance (on mGAM or arabinan) are: B. theta and B. fragilis

This observation is not entirely unexpected, as factors beyond direct competition for the same substrate likely influence community composition among Bacteroidaceae species. Although *B. thetaiotaomicron* and *P. vulgatus* are capable of utilizing arabinan, they are not the dominant members of the Bacteroidaceae niche under arabinan conditions. One possible explanation is that *B. fragilis* encodes a type VI secretion system (T6SS), which can antagonize competing Bacteroidaceae species. In this context, *B. fragilis* may inhibit *P. vulgatus*, but not *B. thetaiotaomicron* as reported previously (PMID: 30825301), allowing it to outcompete *P. vulgatus* even without directly metabolizing arabinan. These findings suggest that interbacterial antagonism, in addition to substrate utilization capacity, may play an important role in shaping niche dominance.

Reviewer #2 (Remarks to the Author):

Tawk et al. performed a screen to decipher dietary components that favor the growth of S. copri or Bacteroides species. Curiously, these do not predict whether Bacteroides dominates over Prevotella or vice versa. They delve into one case, metabolism of arabinan, determining that the predicted competitive edge is dependent on the presence of certain Enterobacteriaceae. Although the authors extend their initial observations for different E. coli strains, different glycans and different Segatella strains, the results are still light on mechanism. Additionally, the conclusions overall are rather limited in terms of understanding why microbiomes are dominated by these species.

Major comments:

1. The researchers jump to the conclusion in line 188 that cross-feeding is impacting the outcome– "direct arabinan utilization is necessary for S. copri expansion within the community, it is not sufficient; interactions with other community members are required for S. copri to dominate..". And surely they find an example of cross-feeding, but it is not clear whether this is the cause for these abundances in complex and natural communities. The authors should demonstrate whether their findings hold up in a complex mixed community with and without E. coli.

Our defined synthetic community provides a controlled and reproducible model to address the variability and complexity of the gut microbiome, enabling systematic investigation of specific microbe-microbe and diet-microbe interactions and their effects on community-level function. Synthetic communities are widely used models that have helped elucidate important mechanisms of microbiome-mediated drug metabolism and colonization resistance in previous studies (PMID: 39321801; PMID: 40670795). Nonetheless, as a reduced system, we agree that they may not capture the full ecological complexity of a native gut microbiota. To address this limitation, and in response to reviewers' suggestions, we also recapitulated our findings in stabilized fecal microbiota communities derived from human donors, which have been shown to be a robust model for microbiota research (PMID: 35051349; PMID: 34646011). First, to validate our findings in more complex communities, we recruited eight healthy donors and reconstituted and stabilized fecal microbiota communities from fresh stool samples (Figure 5A). Stabilized communities were then challenged with *S. copri* alone or in combination with *E. coli* following an established community coalescence approach (PMID: 40063808), with or without arabinan supplementation. Arabinan supplementation consistently promoted a marked expansion of *S. copri* in stabilized human fecal communities, recapitulating dynamics observed in synthetic communities. However, the overall levels of Enterobacteriaceae in these communities were not significantly changed by the addition of *E. coli* (see amended Figure 5), thereby confounding the assessment of *E. coli*-specific effects.

Enterobacteriaceae are common in the human gut microbiome, usually at low relative abundance, and communities without detectable Enterobacteriaceae are rare (PMID: 39794474). To directly investigate whether *S. copri* expansion depends on *E. coli* within complex native communities, we leveraged a large in-house metagenomic cohort to find healthy donors with undetectable Enterobacteriaceae (<0.0001% relative abundance; Extended Data Figure 5D). We identified eight such donors, and their fecal communities were reconstituted and stabilized from frozen stool samples. Consistent with the first cohort (Figure 5A), arabinan increased the relative abundance of *S. copri* within these fecal communities. However, Enterobacteriaceae in these communities expanded during *in vitro* stabilization to levels similar to those in the first cohort. This convergence reflects a general limitation of synthetic and *in vitro* community models, which often show somewhat higher Enterobacteriaceae abundance (see response 8 to Reviewer 1).

To overcome this limitation and further evaluate the specific *E. coli*-*S. copri* interaction in a background devoid of Enterobacteriaceae, we used cecal communities from in-house bred specific pathogen-free (SPF) mice, which in this case naturally lack Enterobacteriaceae (Figure 5B; PMID:

32208465). Notably, in these communities, we observed a specific *E. coli*- and arabinan-dependent expansion of *S. copri* (Figure 5B). Of note, Alpha-diversity analysis of the stabilized fecal and cecal communities revealed significantly greater community complexity compared with the synthetic communities (average observed richness 38-110; average Shannon index 1.5-3.0; Extended Data Fig. 5E-G). These experiments demonstrate that *S. copri* expansion is reproducible across complex human fecal and mouse gut communities and depends on both arabinan and *E. coli*.

2. The authors do not really address why the presence of E. coli which can metabolize byproducts of arabinan metabolism gives P. copri a boost. How does S. copri benefit from this whereas Bacteroides sp. do not. This seems like a central question that is not addressed in the manuscript. Does supplementation with the simple sugars (arabinose, galactose, rhamnose, galacturonic acid) change the outcomes of the competition experiments? The papers the authors cite as examples of cross-feeding in other species go deeper into the mechanism by which the mutualism comes about. They also demonstrate the phenomenon in mouse models.

To gain deeper insight into the nutrient exchange and signaling between *S. copri* and *E. coli*, we conducted additional experiments. As suggested by the reviewer, we tested the direct effects of the simple sugars released during arabinan degradation. Notably, addition of individual sugars or their mixture at concentrations equivalent to the arabinan polymer (arabinose, rhamnose, galactose, and galacturonic acid) promoted *S. copri* expansion in the presence, but not the absence, of *E. coli* (Figure 3G; Extended Data Figure 5A). These results suggest that simple sugars drive *S. copri* expansion via *E. coli*-mediated mechanisms.

To identify the molecule(s) responsible for boosting *S. copri* by *E. coli*, we leveraged our metatranscriptomics and metabolomics data to test several hypotheses regarding the factor(s) that *E. coli* might be providing (see response 5 to Reviewer 1). We first tested tryptophan because arabinan conditions could increase tryptophan levels by downregulating tryptophanase (*tnaA*) in *E. coli,* according to our metatranscriptomics data. However, supplementation with tryptophan or indole (0.5 g/L) did not confer a competitive advantage to *S. copri*. Moreover, *E. coli* Nissle mutants lacking *tnaA* or the tryptophan biosynthesis gene *trpE* still promoted *S. copri* expansion similarly to wild type (Figure R3B), suggesting tryptophan is not the driving factor.

We then examined other pathways highlighted by our metatranscriptomics and metabolomics data and tested known *S. copri* auxotrophies and essential growth components. We generated additional *E. coli* Nissle mutants targeting cytochrome c biogenesis ATP-binding export protein *ccmA*, which was strongly upregulated in arabinan, and the serine acetyltransferase *cysE*, required for cysteine biosynthesis, also recently shown to be implicated in cross-feeding between *E. coli* and *Bifidobacteria* (https://doi.org/10.1101/2025.03.24.645027). However, both mutants still promoted *S. copri* expansion similarly to wild type (Figure R3B). Supplementation with cysteine, hemin, or hematin also had no effect (Figure R3C).

We next tested broader nutrient limitations by supplementing amino acids, purines/pyrimidines, trace elements, glutathione, NAD+, or coenzyme A, but none conferred a competitive advantage to *S. copri*. Likewise, supplementation with succinate (pH 5.0) or lactate did not reproduce the *E. coli*-mediated benefit (Figure R3C).

Overall, across 14 supplementation conditions and four *E. coli* mutants, we could not identify the specific factor(s) responsible, suggesting that pinpointing the mechanism would require more extensive genetic and metabolomic screening beyond the scope of this study as predicted by Reviewer 1. Nonetheless, in addition to carbohydrates, we identified vitamin B3 and B5 as potential contributors to the *E. coli*-driven expansion of *S. copri*, suggesting a possible exchange of vitamin-related molecules between these species (Figure 4B; see Reviewer 1 comment 2; Figure R2).

Although prior studies have revealed cross-talk between *Bacteroides* species in mouse models (PMID: 27111508; PMID: 20603004), these investigations are largely limited to tri-culture experiments of closely related Bacteroidaceae. Our study extends these findings by uncovering inter-phyla interactions within complex microbial communities. By using these communities as an alternative to mouse models, we provide a robust and relevant platform to dissect how commensal interactions shape microbiota composition in response to dietary components. Importantly, we validate these results in both complex human fecal and mouse gut microbiota communities (see response 1), demonstrating the broader relevance of our findings. Finally, it should be noted that research on *Bacteroides* benefits from well-established models and a substantial body of prior knowledge, whereas *S. copri* remains challenging to culture, genetically manipulate, and establish in experimental systems. While we do not yet provide a full mechanistic account of the benefit conferred by *E. coli* to *S. copri*, this study establishes a *S. copri*-specific experimental model and demonstrates that *S. copri* competes with Bacteroidaceae for overlapping nutritional niches, preferentially assimilates a broad range of complex dietary components, which act synergistically with Enterobacteriaceae to promote *S. copri* within gut microbial communities.

3. Similarly, the authors do not demonstrate that the S. copri advantage is dependent on the arabinan byproduct utilization genes in E. coli, which could be tested by knocking out these genes in E. coli. Also, do all of the strains tested in Figure 3C have these genes? Have the authors tested to see whether cross-feeding is actually occurring in these samples by analyzing the fiber degradation and its byproduct usage?

To determine whether *E. coli* utilization of the polysaccharide-derived simple sugars is required to promote *S. copri* expansion, we generated a quadruple *E. coli* mutant (Δ*araBAD* Δ*rhaBAD* Δ*galTK* Δ*uxaCT*) unable to metabolize the arabinan-derived monosaccharides (arabinose, rhamnose, galactose, and galacturonic acid). Interestingly, the *E. coli* quadruple mutant still promoted *S. copri* expansion in the presence of arabinan or the derived simple sugars, despite its inability to utilize these substrates (Figure 3H; Extended Data Figure 5B). This result is similarly observed with two additional fibers that promote *S. copri* expansion in an *E. coli*-dependent manner, arabinoxylan and polygalacturonic acid, along with their derived simple sugars and the corresponding *E. coli* mutants Δ*araBAD* Δ*xylAB* and Δ*uxaCT*, respectively (Figure R4A, see below). Importantly, growth in minimal M9 media confirmed that no other simple-sugar contaminants in the fibers supported appreciable growth of these strains (<30% compared to glucose; Figure R4B). Together, these results indicate that the presence of simple sugars is sufficient to drive *S. copri* expansion within the community, independently of their metabolism, suggesting a signaling rather than nutritional interaction.

Although utilization of simple sugars is not required for *E. coli*-mediated promotion of *S. copri*, their presence nonetheless triggers an expression of the sugar-specific pathways in *E. coli* (Figure 3) and drives an *E. coli*-dependent expansion of *S. copri* within the community. We therefore reasoned that genes involved in simple-sugar transport, sensing, and utilization pathways would be important for this phenomenon. As recommended by the reviewer, we performed an expanded metagenomic analysis to identify Enterobacteriaceae genes involved in the utilization, transport, and sensing of simple sugars derived from dietary polysaccharides (see response 5), including those from the genomes of the strains tested experimentally in this study (Figure 4D). We found that these genes are broadly conserved across Enterobacteriaceae for most of the sugars examined (Figure R4C), suggesting that this sugar-dependent response is widely conserved and likely occurs across diverse Enterobacteriaceae in response to a broad range of fiber-derived sugars.

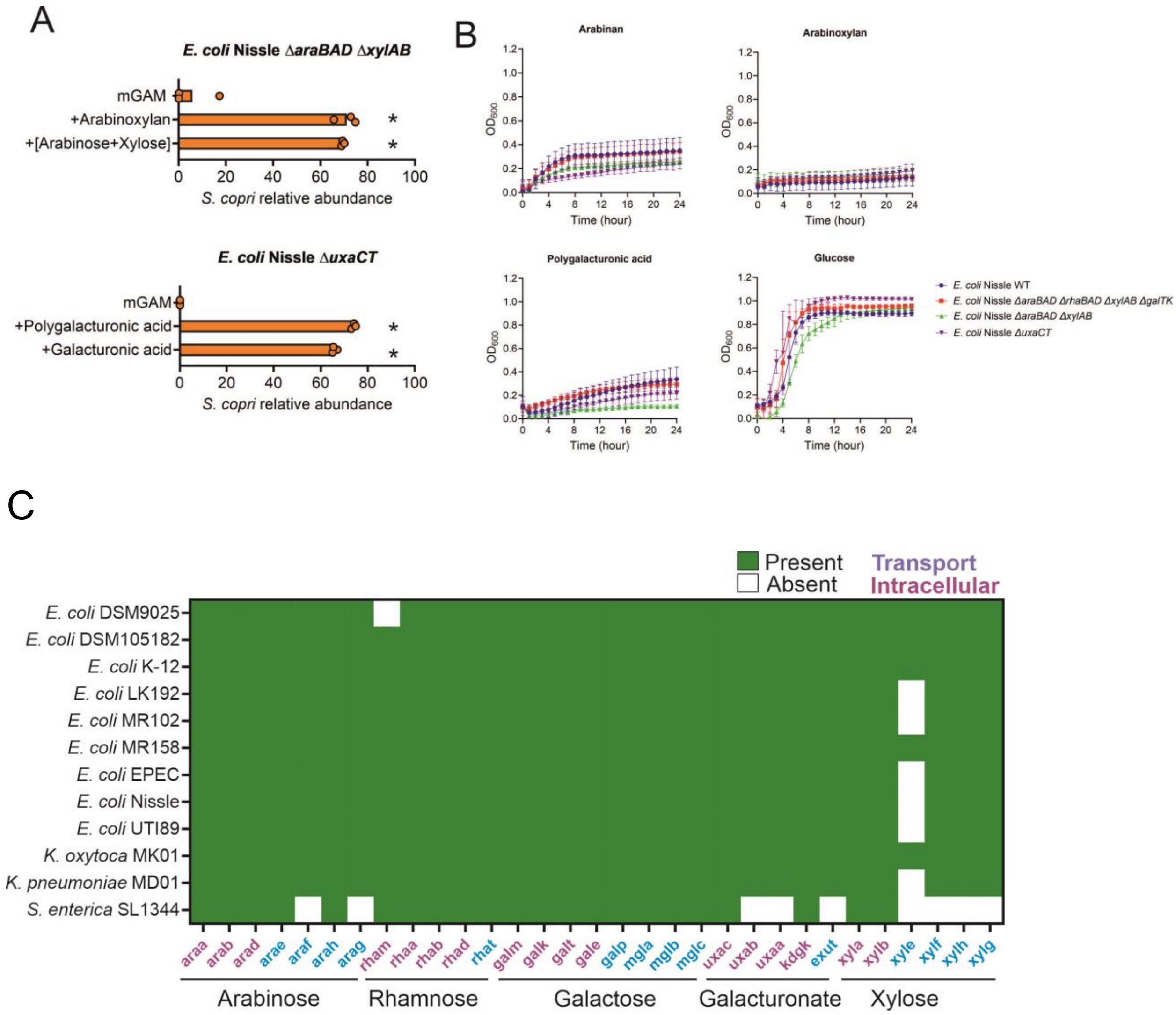

Figure R4.

A. Competitions between *S. copri* and six Bacteroidaceae in the presence of different *E. coli* Nissle strains, fibers and derived simple sugars. The relative abundance of *S. copri* in the communities is shown (n=3). B. Growth curves of the *E. coli* Nissle strains in minimal M9 media with the fibers arabinan, arabinoxylan, polygalacturonic acid, or glucose as control. C. Heatmap showing conservation (≥50% protein identity) of genes involved in simple sugar transport, sensing, and metabolism across the 12 Enterobacteriaceae species tested in this study.

4. The authors find contrary results across Segatella strains. It seems like they do not all obtain the same benefit from E. coli. Could the authors decipher any mechanistic insight from this result?

It is important to note that there are no contrary results across strains from the same species but rather from different *Segatella* species. There is indeed variation in *E. coli*- and arabinan-dependent interactions among *Segatella* species. Notably, this mechanism is conserved in different *S. copri* strains (three strains from distinct donors and countries) as well as in other species, *S. sinica* and *S. sinensis*, but is not uniformly present across the genus, consistent with the phylogenetic divergence within the *Segatella* species complex (PMID: 37883976). Conservation of this mechanism is somewhat associated with phylogenetic proximity: the *S. copri* closely related species, *S. brasiliensis*, *S. sinensis*, and *S. sinica* retain the *E. coli*-dependent

competitive advantage; however, *S. brasiliensis* also exhibits enhanced competitiveness over Bacteroidaceae in the absence of *E. coli* or arabinan, likely reflecting superior resource acquisition relative to other *Segatella* species (Figure 4C and Figure R5). Indeed, *S. brasiliensis* fully outcompetes *P. vulgatus* in mGAM and with arabinan and exhibits partial competition with *B. thetaiotaomicron* (Figure R5). In contrast, more distantly related species (*S. brunsvicensis*, *S. hominis*, *S. sanihominis*) lack *E. coli*-dependent interactions and fail to outcompete Bacteroidaceae under all tested conditions. For *S. brunsvicensis*, this reflects an inability to metabolize arabinan (Extended Data Figure 5C), paralleling the *S. copri* Δ*susC1* phenotype. In *S. hominis* and *S. sanihominis*, which can utilize arabinan, the *E. coli*-dependent mechanism appears to be absent. Notably, these species are less frequently detected in industrialized populations compared to *S. copri* (PMID: 37883976). Collectively, these findings indicate that the fiber synergy with *E. coli* is conserved among *S. copri* strains and present in other closely related *Segatella* species, while absent in more distantly related members, reflecting the extensive phylogenetic diversity within this genus. The high genomic divergence between *S. copri* and *Segatella* species that fail to expand in an arabinan and *E. coli*-dependent manner (e.g., ANI of 86% between *S. copri* HDD04 *and S. hominis* HDD12) reflects substantial evolutionary separation within the broader *S. copri* complex (PMID: 37883976). Comparative genomic analyses of this complex show extensive species-level diversity in gene content, including key functional loci, indicating that these related lineages can differ markedly in ecological behavior despite shared ancestry. Such deep divergence makes it challenging to pinpoint the genetic factors underlying the observed phenotypic differences between *S. copri* and other *Segatella* species.

We have incorporated the following text to further expand the discussion of these findings.

(line 276) "We next investigated whether this fiber-dependent interaction with *E. coli* is conserved beyond the *S. copri* HDD04 strain. Additional *S. copri* strains DSM18205^T and HDC01 also gained an *E. coli*-dependent advantage in the presence of arabinan (Fig. 4c). We then extended our analysis to other *Segatella* species. We observe that conservation of this mechanism is somewhat associated with phylogenetic proximity [26]: the *S. copri* closely related species, *S. brasiliensis*, *S. sinensis*, and *S. sinica* retain the *E. coli*-dependent competitive advantage in the presence of arabinan, with *S. brasiliensis* also outcompetings Bacteroidaceae in the absence of *E. coli* or arabinan, likely reflecting superior resource acquisition relative to other *Segatella* species (Fig. 4c). In contrast, more distantly related species (*S. brunsvicensis*, *S. hominis*, *S. sanihominis*) neither exhibit *E. coli*-dependent interactions nor outcompete Bacteroidaceae under any tested condition: *S. brunsvicensis* cannot metabolize arabinan, whereas *S. hominis* and *S. sanihominis* can but lack the *E. coli*-dependent mechanism (Fig. 4c; Extended Data Fig. 5c). Collectively, these findings indicate that the fiber synergy with *E. coli* is conserved among *S. copri* strains and is present in other closely related *Segatella* species while absent in far related members, reflecting the extensive phylogenetic and ecological diversity within this genus."

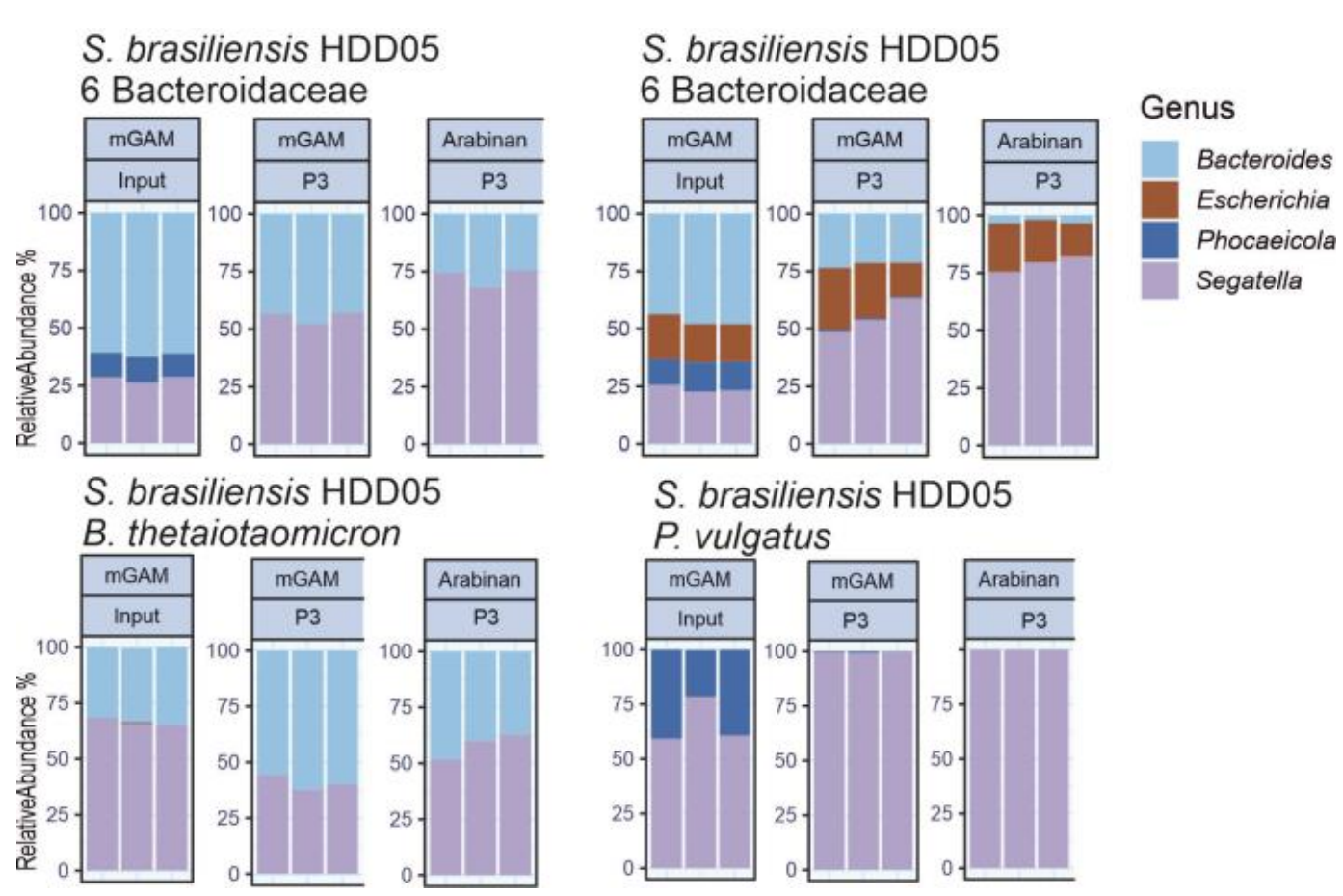

Figure R5.
S. brasiliensis was competed either against the six Bacteroidaceae species in the presence or absence of *E. coli* and with or without arabinan, or in direct 1:1 competition with either *B. thetaiotaomicron* or *P. vulgatus*. Data is from 16S rRNA amplicon sequencing with three replicates shown.

5. The manuscript concludes with a fairly meager metagenomic analysis of microbiomes. The authors did not examine the presence of the genes they deem important in these microbiomes, there is the issue of compositional effects discussed above, and the issue of Segatella strain-level variation in its response to E. coli.
As noted, we did not observe strain-level variation among *Segatella* in response to *E. coli*; the observed differences are at the species level. We focused on *S. copri*, the most abundant and prevalent *Segatella* species (PMID: 37883976), and the tested strains consistently showed *E. coli*-dependent expansion (Fig. 4C). In contrast, more distantly related *Segatella* species (*S. brunsvicensis*, *S. hominis*, *S. sanihominis*) behaved differently. This is expected, as species-level differences within a genus are common and well-documented, including in *Bacteroides*. As the reviewer suggested, we expanded our metagenomic analysis to examine Enterobacteriaceae functions related to transport, sensing, and metabolism of simple sugar (see response 3), as well as the distribution of specifically *S. copri* in the metagenomes. To account for species-level variability within *Segatella*, we focused specifically on *S. copri* genomes and assessed their co-occurrence with Enterobacteriaceae in Industrialized and Non-industrialized gut microbiomes. Consistent with the previous analysis, *S. copri* exhibited significantly higher prevalence and abundance in Non-industrialized populations (Figure 6B). Moreover, in Non-industrialized microbiomes, *S. copri* abundance positively correlated with the number of Enterobacteriaceae (Spearman R=0.18, p=0.00045; Extended Data Figure 6A; see response 9 to Reviewer 1). Although these results are consistent with our observations for overall Prevotellaceae, we chose to exclude the broader Prevotellaceae analysis in accordance with the reviewer's suggestion regarding potential confounding effects of species-level variation within *Segatella*.

Furthermore, we observed that simple sugars derived from fibers induce *E. coli*-dependent *S. copri* expansion within the community, implicating sugar pathways in this effect. To determine whether these functions are enriched in Non-industrialized versus Industrialized microbiomes, we compiled a comprehensive list of 179 Enterobacteriaceae genes involved in 53 different simple sugar transport and metabolism (Supplementary Table 7; based on PMID: 40826220) and used their UniRef50 IDs to assess their distribution across gut metagenomes from Industrialized and Non-industrialized cohorts (n = 870; Fig. 6A). We found that genes associated with transport and intracellular metabolism of diverse simple sugars are significantly enriched in Non-industrialized metagenomes compared with Industrialized ones (Extended Data Fig. 6C). These results indicate that functions potentially involved in mediating the Enterobacteriaceae-*S. copri* interaction in synthetic communities are broadly distributed in Non-industrialized microbiomes, where *S. copri* prevalence is high.

6. Importantly, the authors find that, overall, the capacity to use different glycans did not predict the direction of Prevoteallaceae/Bacteroides ratios in the community (line 138). What is the main mechanism here? The authors do not consider other effects on composition—immune pressure or priority effects, for example. A mouse model would help assess what might be going on here as well (see Gellman et al. Cell Reports 2023). Without this, deeper metagenomic analysis, some idea of mechanism and demonstration that S. copri gets a boost with E. coli in a much more complex mock community, it's hard to ascertain the applicability of their conclusions.

As detailed in the examples following line 138 (now updated to include growth rate measurements), Bacteroidaceae exhibit higher utilization capacity and faster growth on several polysaccharides (e.g., pectic

galactan and arabinan). Nevertheless, *S. copri* outcompeted the Bacteroidaceae within these communities under these same conditions (see response 3 to Reviewer 1). This discrepancy initially prompted us to investigate additional mechanisms and led to the finding that, in the presence of *E. coli*, the competitive advantage shifts toward *S. copri*. In contrast, in the absence of *E. coli*, Bacteroidaceae outcompete *S. copri*, consistent with their superior polysaccharide utilization. Together, these results indicate that direct resource competition alone does not determine *S. copri*/Bacteroidaceae ratios; instead, interactions with other species, particularly *E. coli*, provide an additional competitive advantage that promotes *S. copri* expansion. We have clarified this point in the text by tempering the statement and by including growth rate analyses as follows.

(line 100) "Additionally, the capacity of the different species to utilize the glycans did not always predict the direction of Prevotellaceae/Bacteroidaceae ratios within the community. For instance, pectic galactan is utilized by *B. caccae*, *B. thetaiotaomicron*, *B. ovatus*, *B. uniformis*, *P. vulgatus*, and *S. copri* with calculated growth rates 0.62 h-1, 0.5 h-1, 0.5 h-1, 0.39 102 h-1, 0.18 h-1, and 0.13 h-1, from highest to lowest, respectively. When pectic galactan is supplemented to the community, it leads to an increase in *S. copri* within the community and not the Bacteroidaceae species (Fig. 1d, Extended Data Fig. 1d). Further, *B. thetaiotaomicron*, *P. vulgatus*, and *S. copri* all utilize arabinan (at descending growth rates 0.5 h-1, 0.42 h-1, 0.12 h-1), when added, it leads to an expansion of *S. copri* and a depletion of the Bacteroidaceae members (Fig. 1d, Extended Data Fig. 1d). These experiments demonstrate that assessing the utilization capacity of dietary components by individual commensal species in monoculture does not in most cases reliably predict the overall community dynamics. Other factors of bacterial cross-talk, in addition to the utilization of complex carbohydrates, likely drive community structure and composition."

Additionally, we addressed the points raised by the reviewer and aimed at gaining further mechanistic insights into these dynamics. We further investigated the mechanism of *S. copri*-*E. coli* cross-talk by generating three additional *E. coli* Nissle mutants and confirmed the *S. copri*-*E. coli* fiber-dependent interactions in complex communities, including fecal microbiota from two independent human cohorts and SPF mouse cecal communities (Figure 5). We also expanded our metagenomic analysis by performing detailed functional profiling of ~1,000 metagenomics samples. Previous studies have shown that *Segatella*/*Bacteroides* ratios within the gut microbiota are primarily associated with diet and lifestyle rather than host genetics or immune factors (PMID: 21885731; PMID: 30799264). Accordingly, we focused on dietary inputs and microbial ecology to identify factors that shift the Prevotellaceae/Bacteroidaceae balance. Using controlled community models, we experimentally demonstrate that these taxa compete for overlapping nutrient niches, display distinct polysaccharide preferences, and depend on interactions with additional community members, notably Enterobacteriaceae, to gain a competitive advantage. Mechanistically, we show that *E. coli*-mediated promotion of *S. copri* does not require the utilization of simple sugars but is triggered by the presence of excess sugars. Using extensive metabolomic and metatranscriptomic profiling, we tested 14 supplementation conditions and four additional *E. coli* Nissle mutants, and were unable to identify a single causal factor, suggesting that resolving this mechanism will require broader genetic and systems-level approaches beyond the scope of this study (As pointed out by Reviewer 1; see response 2 to Reviewer 1). Notably, our results show that vitamin B3 and B5 promote *S. copri* expansion in an *E. coli*-dependent manner, indicating that *S. copri*-*E. coli* interactions extend beyond carbohydrates but also include vitamins.

Analytical comments:

7. The correlation analysis of the abundances of organisms in 16S is not as simple as the competitive index the authors use. There are compositional effects that have been well documented in the literature and are customarily used in 16S rRNA analysis to study correlation. SPARCC is an old and commonly used method.

Others include SPIEC-EASI and SECOM, for example. Additionally, the authors may have true abundance data they can use. This is important as just comparing percent abundances after some perturbation may lead to spurious results.

We agree with the reviewer that analysis of relative abundances using 16S rRNA amplicon sequencing is subject to compositional data bias. To address this concern and as suggested by the reviewer, we analyzed the correlation between *S. copri* and Bacteroidaceae using SparCC, an established method designed to mitigate compositional bias in microbial community analyses (PMID: 23028285). Applying SparCC to the communities shown in Figure 2 revealed that *S. copri* anti-correlates with all Bacteroidaceae species except *B. caccae*, which remains at low relative abundance across all conditions, consistent with our observations. Significant anti-correlations were observed with *P. vulgatus*, *B. uniformis*, and *B. ovatus* (Figure R6).

Because the statistical power of SparCC may be limited in the relatively lower complexity of the synthetic communities compared with native microbiomes, we additionally quantified absolute DNA abundance using species-specific qPCR and standard curves for each strain. Absolute quantification of each strain, followed by calculation of the log ratio between *S. copri* and the six Bacteroidaceae species similarly reflected the competitive advantage of *S. copri* in *S. copri*-promoting communities, consistent with the patterns observed in the 16S rRNA amplicon sequencing data (Extended Data Figure 2E).

Finally, considering the comparable total cell counts measured in mGAM and arabinan conditions and the similar final OD600 values across conditions (Extended Data Figure 1), we conclude that the relative abundance estimates derived from the 16S data are reliable for these comparisons.

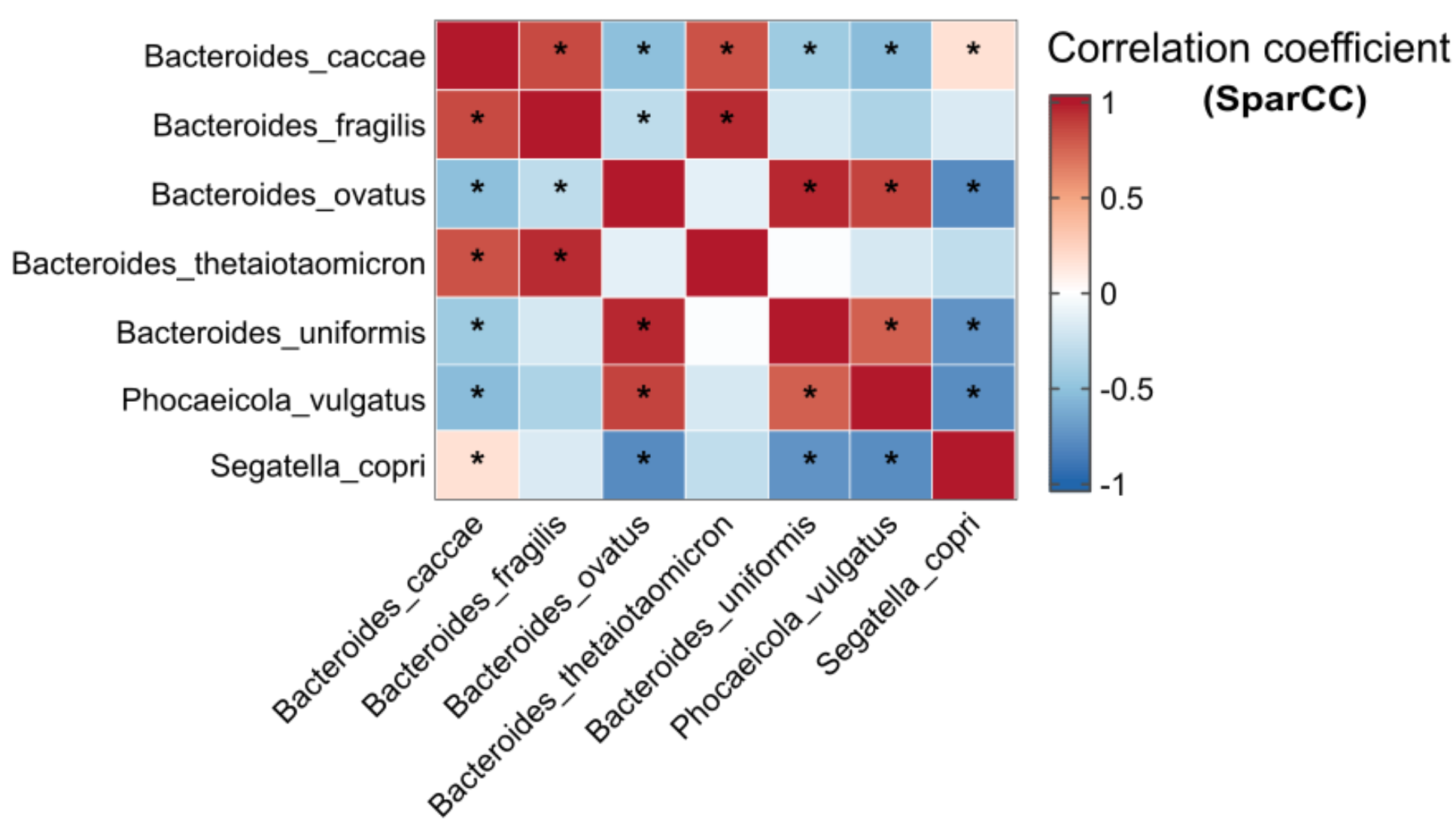

Figure R6. Correlations between community members across community settings were estimated using SparCC (PMID: 23028285) with 20 inference iterations. P-values were assessed using the SparCC utilities MakeBootstraps.py and PseudoPvals.py with 20 simulations (https://github.com/dlegor/SparCC).

8. The food analysis may be a red herring. The authors imply that individuals obtain E. coli or other Enterobacteriaceae strains from the food they eat. These conclusions seem disjointed from the rest of the manuscript. Analysis of transmission requires strain-level resolution for conclusive evidence. It could very

well be that the strains found in food products are not those that are found in the gut microbiome. Most data suggests that bacteria from food are incapable of colonizing or even surviving in the gut and therefore appear as transient members of the microbiome.

Given the strong reported association of *Segatella* prevalence with Non-industrialized diets, our finding that Enterobacteriaceae synergize with dietary components to promote *S. copri*, and the high transmissibility of Enterobacteriaceae, particularly through the environment (PMID: 36653448; PMID: 28603278), we hypothesized a link between increased Enterobacteriaceae carriage in the Non-industrialized populations and their presence in the dietary environment. In line with the reviewer's suggestion, we reanalyzed an expanded food metagenome database (cFMD, comprising 3,310 metagenomes from 52 countries and 15 food categories; Figure 6; see Methods). Results mirrored our initial analysis: Enterobacteriaceae prevalence and relative abundance were highest in plant-derived foods (fermented and raw), and foods with greater Enterobacteriaceae prevalence and abundance predominantly originated from Non-industrialized countries (Figure 6F-G). Fisher's exact test confirmed enrichment of Enterobacteriaceae SGBs in Non-industrialized and plant-based foods, consistent with our initial observations (Figure 6F-G).

To further assess potential sharing between the human gut microbiome and foods, we performed strain profiling of Enterobacteriaceae SGBs in food and stool samples from Industrialized and Non-Industrialized cohorts. We selected SGBs enriched in both environments and reconstructed strain-level phylogenies. Notably, closely related strains of *E. coli* and *Enterobacter hormaechei* were identified in both human and food samples, indicating the presence of highly similar Enterobacteriaceae across these samples (Figure R7). We did not detect identical strains shared between datasets, which is expected given their independent sampling across different times and locations; resolving direct sharing will require studies with matched human-food sampling. Although food-associated probiotic strains are often considered transient in the gut, prior studies have reported strain sharing between humans and food or water (PMID: 39214080; PMID: 37116481; PMID: 40312516). The directionality of transmission and persistence of these strains in the gut will require targeted design and longitudinal data beyond the scope of this study.

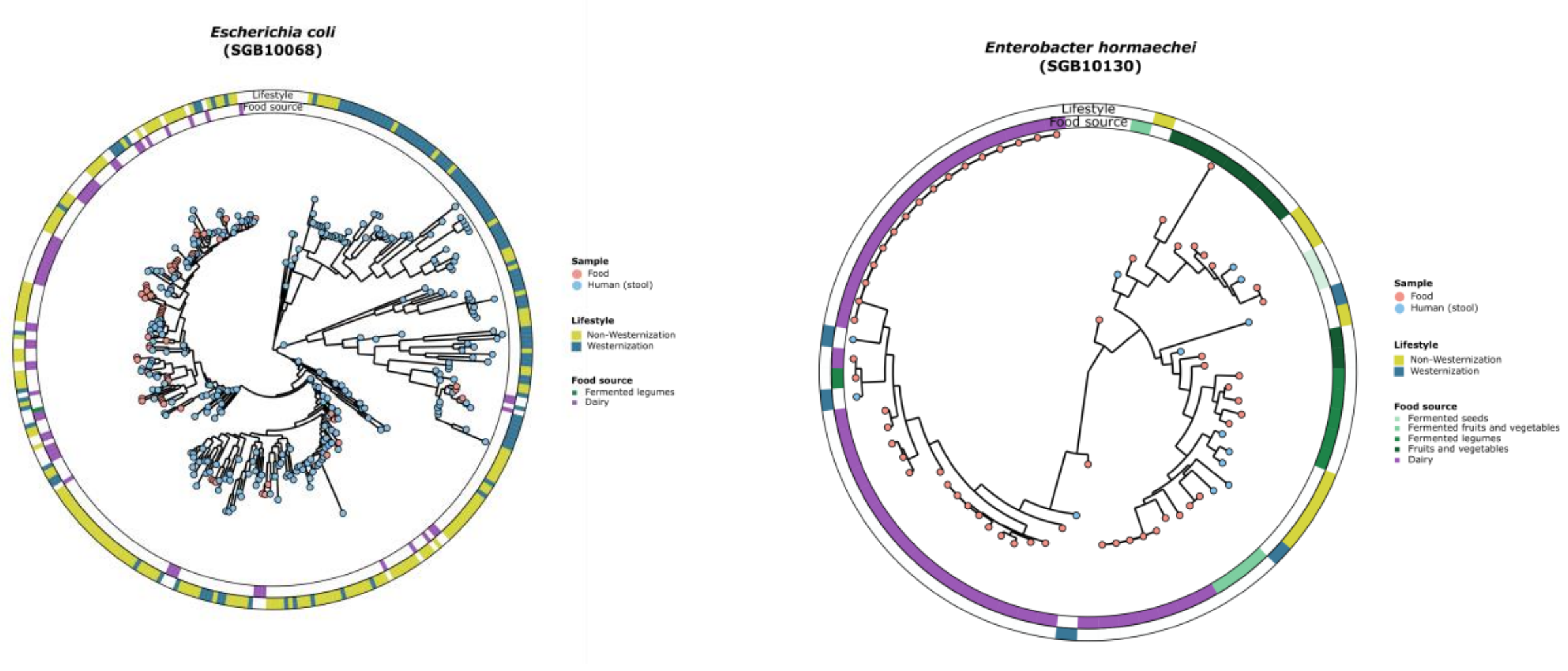

Figure R7. Phylogenetic trees representing reconstructed strain-level phylogenies for *E. coli* (SGB10068) and *Enterobacter hormaechei* (SGB10130), which showed significant joint enrichment to a food category and stool samples of Non-industrialized lifestyle.

Minor comments:

9. The data in Figure 3C is presented nonintuitively. Why not show the growth curves? Given the variability, it is surprising this was only done in duplicate.

Due to space limitations in the figures and the complexity of the growth curves, we chose to keep the AUC representation in the main figure as a representative of three experiments. However, we performed an additional replicate, and the corresponding growth curves, OD_{max} and calculated AUCs for all three replicates are reported in Extended Data Figure 3.

10. Arabinan is a common glycan found in many fruits and grains. How do the authors conceptualize these findings given that arabinan is commonly available globally.

Although arabinan and other plant-derived polysaccharides examined here are globally ubiquitous, their consumption is substantially reduced in industrialized diets, where raw fruits, legumes, and whole grains are often replaced by processed foods enriched in modified starches and fats, and relatively deficient in dietary fiber (PMID: 31089293). Moreover, both our findings and previous studies indicate that diet alone does not fully explain the distribution of *Bacteroides*- versus *Prevotella*-dominated microbiota profiles. Our results identify an additional ecological factor: Enterobacteriaceae promote the dominance of *S. copri*. Thus, the presence, transmission, and acquisition of these taxa may significantly influence community composition. Given that Enterobacteriaceae are also commonly associated with raw foods, their introduction through dietary exposure may further contribute to the establishment of *Segatella*-rich microbiomes, beyond the effects of fiber intake alone.

Reviewer #3 (Remarks to the Author):

The manuscript by Tawk et al. describes a series of experiments that reveal ecological dynamics of gut microbes in response to different dietary substrates, particularly Segatella copri, E.coli, and arabinan. I commend the authors on designing and executing a robust set of experiments, and for a well-written and compelling presentation of their work. The findings advance understanding of the interplay between diet and microbiota and, as such, are valuable to the field and can inspire future avenues of research.

The following are specific comments that the authors should address:

1. I wonder if the title could be a bit more specific. The authors focus mainly on E. coli in the results, whereas Enterobacteriaceae as a family seems to only be presented in the last section on comparing Westernized vs Non-Westernized populations (which I also have a comment on, see #18). Also, instead of 'dominant anaerobes', perhaps specify the competition between Bacteroidaceae and Prevotellaceae?

We modified the title as suggested by the reviewer to make it more specific. However, because we demonstrate that the interaction with *S. copri* is widely conserved across diverse Enterobacteriaceae species (Figure 4D, see comment 15), we chose to retain a general reference to Enterobacteriaceae in the title.

"Synergy between Enterobacteriaceae and dietary components shapes the competition between the dominant Bacteroidales of the human gut."

2. Line 30: I suggest using the terms ‘industrialized’ and ‘non-industrialized’ vs. words like ‘modern’ and ‘Westernized’ throughout the abstract and main text. I would argue all populations currently on Earth are ‘modern’ (in the sense of time, not level of advancement). With industrialization becoming more and more widespread to countries beyond those in North America and Europe, ‘Western’ isn’t an appropriate term anymore, either.

We agree that selecting terminology to distinguish human populations by geography and lifestyle is challenging. We appreciate the reviewer’s suggestion and agree that *industrialized* and *non-industrialized* are more appropriate terms. We have updated the terminology throughout the text and figures accordingly.

3. Line 60: if ‘Westernized’ is changed to ‘industrialized’, then ‘industrialization’ can be removed later on in the sentence.

This change has been introduced in the text.

4. Line 73: CAZymes does not need to be defined again (already defined in line 71).

We thank the reviewer for spotting this redundancy. We corrected it.

5. Figure 1: suggest using a different colour for 'Synthetic', as the shade of green is a bit hard to distinguish from that of 'Bacterial'. Perhaps a light purple?

Following the reviewer’s recommendation, we changed the color code for “Synthetic” polysaccharides to teal to distinguish them from other colors used in the panel.

6. Lines 101-103: the vast majority of diet-derived vitamins are absorbed in the small intestine and would not be available to microbiota in the large intestine. I assume, here, you mean those vitamins that are synthesized by bacteria? If yes, I suggest making this clearer, as diet-derived vitamins likely do not significantly influence the microbiota. Also related to this, in Line 106, what exactly are you referring to as ‘physiological ranges’ of the vitamins? In the human gut?

We agree with the reviewer that the extent to which dietary vitamins reach the large intestine remains uncertain. Nevertheless, multiple studies, clinical trials, and gnotobiotic mouse experiments demonstrate that exogenous vitamin supplementation significantly alters the composition of the gut microbiota (PMID: 33615992; PMID: 37119822; PMID: 40645132; PMID: 28111202). Whether these effects are direct or mediated through host physiology remains to be determined. Importantly, the role of vitamins and competition for them among gut commensals, whether synthesized by members of the microbiota or supplied exogenously, in shaping community dynamics is well established (PMID: 37054671) and was a focus of our study.

We further examined vitamin effects and found that vitamin B3 and B5 promote *S. copri* within the synthetic community in an *E. coli*-dependent manner, similar to polysaccharides (Figure 4B, Figure R2; see response 2 to Reviewer 1). This supports the conclusion that the *S. copri*-*E. coli* interaction is mediated by diverse compounds, including dietary polysaccharides and exogenous vitamins. To approximate physiological vitamin supplementation conditions, we used vitamin concentrations comparable to those employed in human clinical and mouse studies (PMID: 33615992; PMID: 37119822; PMID: 28111202). Although the presence of these concentrations in the distal gut remains debated, extensive microbial competition and the strong biological effects observed suggest that even low vitamin levels could meaningfully influence microbe-microbe interactions and community structure in the gut (PMID: 37054671), including the interactions demonstrated here. Further studies will be required to elucidate the

specific mechanisms underlying vitamin-mediated interactions of *S. copri* and *E. coli*, particularly those involving B vitamins.

We added a statement addressing this point in both the results and discussion sections.

(line 65) "…and the vitamins were added at their specific physiological supplementation ranges in dietary interventions …"

(line 444) " However, the mechanisms underlying vitamin-mediated interactions and the concentrations available in the distal gut, particularly from dietary intake, have yet to be fully understood."

7. Line 117: suggest listing some examples of what 'gelling compounds' you are referring to here.

We added examples of gelling compounds to the text, and a note in Supplementary Table 2 listing all gelling compounds.
(line 76) "…some gelling compounds (e.g. agarose, carrageenan, curdlan) affecting the final OD600 absorption…"

8. Figure 1D: I was surprised that the authors do not mention E. coli in this paragraph when Figure 1D is referenced (starting at line 113), even though it is clear that E. coli increased in response to almost as many compounds as S. copri. What was the reasoning for not highlighting E. coli here? Also, it seems like the number of compounds that increased the abundance of E. coli does not match the number listed in Figure 1F. The magnitude seems to be quite high and similar to that of S. copri, and yet E. coli significantly increased less than a third of what S. copri did? All in all, I think it is worthwhile mentioning E. coli much earlier on in the manuscript, especially in reference to Figure 1D and Figure 2A/D. I believe the first mention of E. coli is not until line 202 and Figure 3A/B.

The results shown in Figure 1F include only statistically significant changes (FDR < 0.05; Kruskal-Wallis with false discovery rate correction). Although Figure 1D shows appreciable fold-changes in *E. coli* relative abundance within the community, these changes are less statistically robust than those observed for *S. copri* and *P. vulgatus*, for example. We provide the corresponding FDR values in the Source Data Table for Figure 1. Consistent with the reviewer's observation, *E. coli* relative abundance tends to fluctuate substantially in response to multiple compounds in the community; accordingly, we have added an earlier reference to *E. coli* in the text.

(line 84) "*E. coli* relative abundance exhibited considerable variability in response to multiple compounds within the community, although only a minority of these changes reached statistical significance (n=17; 38% of the compounds; Figure 1d, 1f)."

9. Line 137: is Figure 1F the correct figure to reference here? It's not clear to me how this bar graph shows that most glycans promote S. copri growth at the expense of Bacteroidaceae.

We corrected the figure citation for this result in the text as follows:

(line 78) "Interestingly, despite *B. ovatus* growing on the largest number of compounds, and other Bacteroidaceae utilizing a similar number of compounds to *S. copri* (>25 % growth as a fraction of growth in mGAM), most glycans promoted *S. copri* growth in the community at the expense of the Bacteroidaceae (Ratio *S. copri*/6Bac.) Fig. 1d; Extended Data Fig. 1c)."

10. Figure 3: Overall, I commend the authors for conducting such systematic experiments, and for presenting the results in a very easy-to-understand manner! For Figure 3C, can the Calculated AUC legends be altered

so that they are all the same range? This would allow for easier comparisons among the three heatmaps.

We thank the reviewer for the positive feedback.

The three species, particularly *E. coli*, exhibit markedly different anaerobic growth dynamics in mGAM (with distinct lag and exponential phases and OD_{max}). Consequently, AUC ranges differ substantially between Bacteroidales and *E. coli*, and plotting them on the same scale reduces clarity. Because our analysis focuses on within-species comparisons (e.g., *S. copri* alone vs. with *E. coli*), AUC remains an appropriate representation of growth. We have now included all three replicates with their full growth curves and corresponding AUC values (Extended Data Figure 3A). Additionally, we added OD_{max} data with statistical analysis across replicates (Extended Data Figure 3B). All metrics support the same conclusion: *E. coli* growth is enhanced by *S. copri* and the six Bacteroidaceae (6Bac.), independent of medium; Bacteroidaceae growth is enhanced by *E. coli* (and modestly by *S. copri*); and *S. copri* growth is enhanced by *E. coli,* particularly in the presence of arabinan. Due to space constraints, we kept the main Figure 3 showing AUC data from one representative experiment, while all replicate data are now provided in Extended Figure 3.

11. Extended Data Fig. 3: is there a need to list A/B/C when there's no specific mention of the different panels in the manuscript?

We updated Extended Figure 3 to include all three co-culture replicates with growth curves and corresponding AUC calculations (Extended Fig. 3A) and added a panel showing ODmax values derived from these curves (Extended Fig. 3B).

12. Lines 240-243: suggest revising the sentence (for clarity) that describes the pathways contributed by E. coli. It took me a few read-throughs to understand its meaning.

We have rephrased this part accordingly as suggested by the reviewer.

(line 207) "However, several pathways such as purine and pyrimidine metabolism, amino acid metabolism, and nicotinate/nicotinamide metabolism were consistently enriched in communities containing *E. coli* ("with *S. copri*" and "without *S. copri*"), but not in communities lacking *E. coli* ("without *E. coli*"). This pattern suggests that the enrichment of these metabolic functions is likely driven by the presence of *E. coli* in the community (Extended Data Fig. 3c)."

13. Lines 265-269: do the authors have data to support at what timepoint/hour these changes occurred? If so, it would be interesting to include (similar to how the authors noted that genes in PUL14 were upregulated at 8h, line 258).

All community metatranscriptomic data shown in Figure 3 were analyzed at the 8 h time point; this has now been specified in both the main text and the figure legend.

14. Figure 4B: can the authors comment on Segatella brasiliensis HDD05 (clade D), which appears to be the only one that grows without E. coli? It would be worthwhile to comment on this in the manuscript, and briefly speculate why.

We have provided some additional data and an expanded discussion on the variation in *E. coli*- and arabinan-dependent competitive interactions among *Segatella* species (Figure R5; see response 4 to Reviewer 2). Interestingly, while *S. brasiliensis* retains the *E. coli*-dependent competitive advantage, it also shows

enhanced competitiveness over Bacteroidaceae in the absence of *E. coli* or arabinan, likely reflecting superior resource acquisition compared to other *Segatella* species (Figure R5 and Figure 4C). Indeed, *S. brasiliensis* fully outcompetes *P. vulgatus* in mGAM and with arabinan and exhibits partial competition with *B. thetaiotaomicron* (Figure R5). This example of *S. brasiliensis* suggests that additional mechanisms, such as direct competition, interactions with other members of the gut microbiota, or the utilization of alternative dietary fibers, as reported for *S. brunsvicensis*, may operate within the broader *Segatella* species complex to maintain the prevalence and abundance of the Prevotellaceae-associated niche characteristic of non-industrialized microbiomes. Collectively, these observations highlight the considerable phylogenetic and functional diversity within the *Segatella* genus (PMID: 37883976).

We have incorporated the following text to further expand the discussion of these findings.

(line 276) "We next investigated whether this fiber-dependent interaction with *E. coli* is conserved beyond the *S. copri* HDD04 strain. Additional *S. copri* strains DSM18205^{T} and HDC01 also gained an *E. coli*-dependent advantage in the presence of arabinan (Fig. 4c). We then extended our analysis to other *Segatella* species. We observe that conservation of this mechanism is somewhat associated with phylogenetic proximity [26]: the *S. copri* closely related species, *S. brasiliensis*, *S. sinensis*, and *S. sinica* retain the *E. coli*-dependent competitive advantage in the presence of arabinan, with *S. brasiliensis* also outcompetings Bacteroidaceae in the absence of *E. coli* or arabinan, likely reflecting superior resource acquisition relative to other *Segatella* species (Fig. 4c). In contrast, more distantly related species (*S. brunsvicensis*, *S. hominis*, *S. sanihominis*) neither exhibit *E. coli*-dependent interactions nor outcompete Bacteroidaceae under any tested condition: *S. brunsvicensis* cannot metabolize arabinan, whereas *S. hominis* and *S. sanihominis* can but lack the *E. coli*-dependent mechanism (Fig. 4c; Extended Data Fig. 5c). Collectively, these findings indicate that the fiber synergy with *E. coli* is conserved among *S. copri* strains and is present in other closely related *Segatella* species while absent in far related members, reflecting the extensive phylogenetic and ecological diversity within this genus."

15. Did the authors test pathogenic strains of E. coli (e.g., E. coli O157:H7)? Most individuals in the nutrition field, and the general public for that matter, would probably associate E. coli with illness (without considering strain-specificity or context dependency), and might question if E. coli promoting S. copri through specific dietary substrates would be potentially harmful for the host. If this was not tested, I wonder if it should be. If it was tested but not included in the results, I would be interested to know why and what the potential implications are for the host.

We tested additional pathogenic Enterobacteriaceae strains, including enteropathogenic *E. coli* (EPEC) and clinical *E. coli* isolates from diarrhea and bacteremia, as well as *Salmonella enterica* serovar Typhimurium. We have now expanded the analysis to 12 Enterobacteriaceae strains spanning three genera (*Escherichia*, *Salmonella*, and *Klebsiella*), including both commensal and pathogenic representatives (Figure 4D). These experiments show that the *S. copri* interaction mechanism is broadly conserved across diverse Enterobacteriaceae. This cross-talk likely evolved between *S. copri* and commensal Enterobacteriaceae in the gut, with pathogenic strains subsequently exploiting the same interaction. A similar phenomenon has been described for *Bacteroides*-enteric pathogen interactions, in which *Bacteroides* promotes pathogen infection by increasing mucosal carbohydrate availability for the pathogen (PMID: 26303108; PMID: 23995682; PMID: 37054671). Our findings suggest that *E. coli* can benefit similarly from both *S. copri* and Bacteroidaceae, whereas the benefit provided by the *E. coli*-fiber synergy appears to be more specific to *S. copri*. Based on this observation, we speculate that *S. copri* is unlikely to confer a selective advantage to pathogenic Enterobacteriaceae species. However, Further studies will be required to investigate the specific

S. copri-enteric pathogen interactions during infection and their role in pathogen establishment. We incorporated this new data into the manuscript and included a statement in the discussion.

(line 447) "Studies have shown that *Bacteroides sp.* engage in mutualistic cross-feeding with pathogenic and commensal Enterobacteriaceae species [25,54-56]. Our results show that similarly *S. copri* engages in a nutrient cross-talk with pathogenic and commensal Enterobacteriaceae species."

16. Have the authors looked at data from PMID: 26552345, and whether responders who had enrichment in P. copri may have also harboured E. coli? This comparison might serve as further validation for the current manuscript's findings, in addition to the analysis of industrialized vs non-industrialized populations.

Kovatcheva-Datchary et al. (PMID: 26552345) reported a 3-day dietary intervention in which healthy Swedish participants consumed barley kernel-based bread (BKB), showing that higher *Prevotella/Bacteroides* ratios were associated with improved glucose metabolism. We extracted average relative abundances of *S. copri*, Bacteroidaceae, and Enterobacteriaceae in responders and non-responders. Consistent with the cohort's geographic context, Enterobacteriaceae richness was very low (mean = 1 species/group; maximum = 3), and *E. coli* was not detected in any group, likely reflecting limited sequencing depth rather than a true absence. As reported, responders had higher baseline *S. copri*, which increased after intervention, whereas non-responders showed higher baseline Bacteroidaceae with a modest post-intervention increase (Figure R8). Enterobacteriaceae were higher at baseline in non-responders but decreased after intervention; conversely, responders exhibited a marked post-intervention increase in Enterobacteriaceae despite low baseline levels.

Although this dataset offers a valuable snapshot of the relationship between dietary fiber and *Prevotella/Bacteroides* dynamics, the low Enterobacteriaceae detection limits interpretation. Larger cohorts and deeper sequencing will be needed to resolve these interactions in future dietary interventions. Given these limitations, we did not include this analysis in the manuscript but incorporated it into the discussion of study limitations and future directions, as requested by the reviewer.

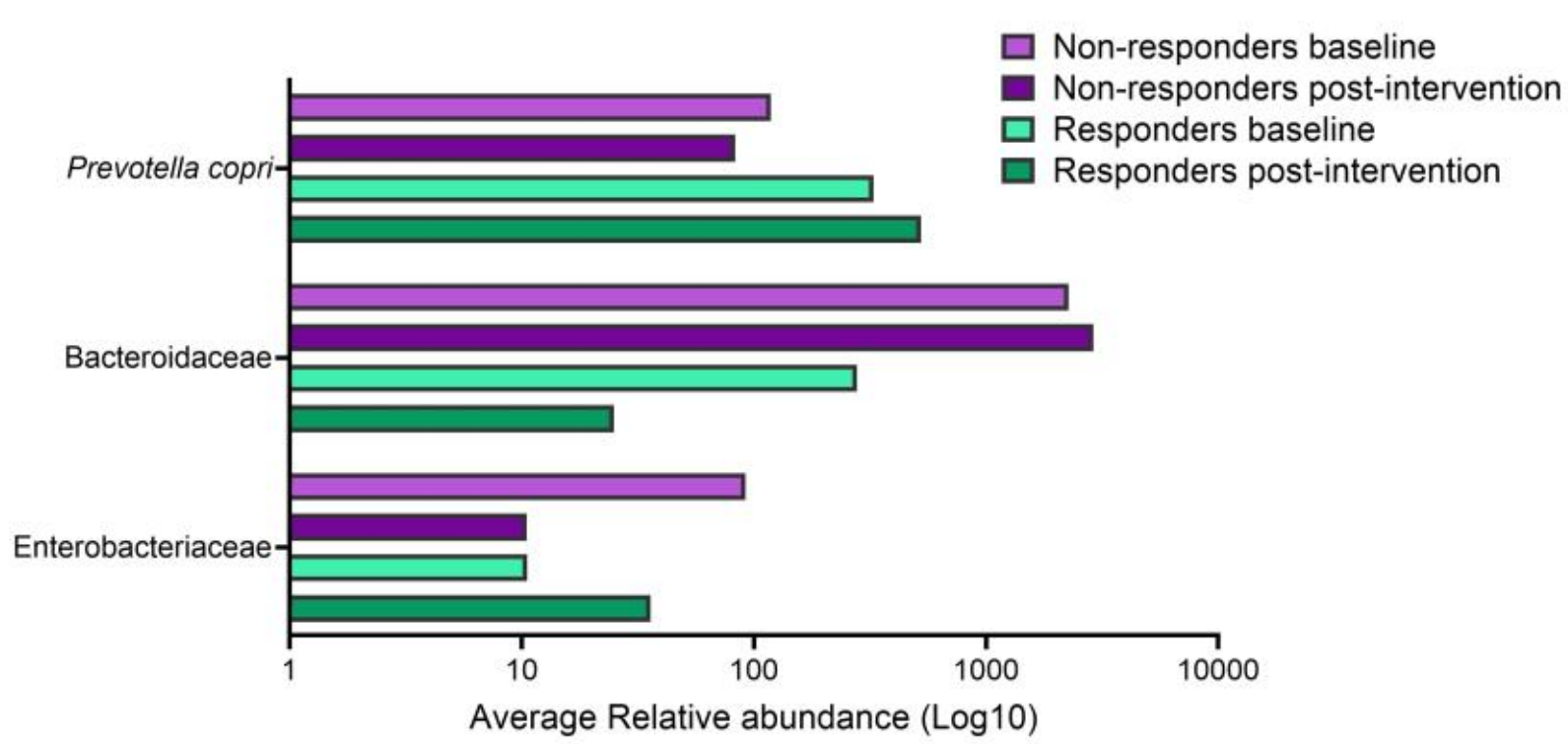

Figure R8. Average relative abundances of *Prevotella copri*, Bacteroidaceae, and Enterobacteriaceae are shown from the dietary intervention study in Kovatcheva-Datchary et al. (PMID: 26552345).

17. Line 324: please provide correlation coefficients and p values for significant correlation results.

We provided correlation coefficients and p-values in the text as requested by the reviewer in line 350.

18. Line 326 and Figures/Extended Data Figures 5: what is the justification for looking at Enterobacteriaceae

and Prevotellaceae as families, and not E. coli and S. copri as species? I would include this justification in the text, or also present correlations between the species in the manuscript, if possible.

As requested by the reviewer and because our mechanistic analyses focus on beneficial interactions with *S. copri* specifically (which we find are conserved across some gut *Segatella* species), we provide a species-level analysis of *S. copri* (Figure 6B and Extended Data Figure 6A). Consistent with the previous analysis, *S. copri* exhibited significantly higher prevalence and abundance in Non-industrialized populations and positively correlated with the number of Enterobacteriaceae (Spearman R=0.18, p=0.00045; Extended Data Figure 6A). Although these results are consistent with our observations for overall Prevotellaceae, we chose to exclude the broader Prevotellaceae analysis in accordance with the reviewer's suggestion. However, based on our additional experiments with diverse Enterobacteriaceae strains and expanded metagenomic analyses (see response 5 to Reviewer 2), we find that the beneficial interaction with *S. copri* is conserved across the Enterobacteriaceae and not restricted to *E. coli*, which we use as a representative model. We therefore include analyses at the Enterobacteriaceae family level in both the human and food datasets, where these interactions are likely to extend beyond *E. coli*.

19. Lines 353-356: is this simply a reflection of fruits and vegetables being watered when grown, which may be the source of Enterobacteriaceae, and not because of the fibre content of these foods?

Based on the literature and our results, we observe a distinct requirement for fiber-rich foods to enhance *S. copri* abundance in gut communities, which can synergize with Enterobacteriaceae. However, the presence of Enterobacteriaceae on food items is unlikely to be driven by fiber content, as *E. coli* cannot directly utilize these fibers. Interestingly, our extended analysis of the food microbiome (Figure 6D-G) shows enrichment of Enterobacteriaceae species in plant-based compared to animal-based foods, likely reflecting exposure to fecal material via contaminated water or soil, as noted by the reviewer. Supporting this, previous studies have demonstrated the direct transmission of Enterobacteriaceae strains to humans and among humans via fecal contamination of water (PMID: 40312516). Thus, we hypothesize that a higher burden of fecal contamination may increase human acquisition of these strains, potentially influencing overall gut microbiota dynamics.

20. Discussion: I would like to see the authors describe some of the limitations of their work, and also list a few clear future directions/what experiments could be conducted in humans to validate the findings further. Can a similar approach be applied for other dietary fibres and keystone species or primary/secondary degraders? What do these results mean for host health? Are synbiotic applications a potential future direction to elicit more consistent responses to dietary fibres or probiotic interventions?

We have added a section on study limitations and future directions to the discussion, as requested by the reviewer.

(line 479) "Synthetic and human-derived fecal communities are valuable models for studying microbial networks, though they are limited by reduced complexity and may not fully recapitulate *in vivo* conditions. However, experimental designs like ours can reveal interactions between xenobiotics, keystone species, and primary or secondary degraders, providing mechanistic insights into microbiome assembly and responses to perturbations. Additionally, our findings can guide dietary interventions by considering both supplemented components and baseline microbiome composition, supporting personalized and combinatorial pre- and probiotic strategies."

Point-by-point response:

Reviewer #1 (Remarks to the Author):

Authors did a remarkable job on responding to critical comments and questions of the original review including multiple additional experiments that helped to clarify most important issues. All responses are thorough and detailed, and the edits/additions to the MS are adequate and helpful. As a result tha MS was significantly improved and is recommended for publication in its current form. Adequate statistical tests were applied and duly reflected in the illustrations.

Authors' Response: We are grateful to the reviewer for their suggestions, which helped improve the manuscript throughout the review process. We especially appreciate the reviewer's positive feedback on the revised version and for supporting the publication of our study.

Reviewer #2 (Remarks to the Author):

I thank the authors for putting in a significant amount of additional experiments, as requested. Unfortunately, several of these key experiments now raise new questions rather than support their original claims. They show that Segatella expansion occurs with arabinose and without E. coli. Their mouse experiments Extended Data Fig. 5 were not done without Nissle. Importantly, their knockout of the arabinose utilization genes also does not support the cross-feeding hypothesis. The authors appropriately changed their conclusions to "sugar-dependent signaling", but it's not clear what this mechanism is or its relevance. I do not suggest any additional experiments since that seems beyond the scope of this manuscript.

Authors' Response: We appreciate the reviewer's suggestions that improved the manuscript during the review process. We are grateful to the reviewer for acknowledging the substantial additional experimental work performed during the revision and for recognizing that further experiments are beyond the scope of the current manuscript.
Before a more detailed response to the interpretation and relevance of our findings, we would like to clarify several aspects of the reviewer's assessment that may have contributed to the impression that the new experiments "raise new questions rather than support the original claims." We believe this impression may stem from some misinterpretations of the experiments. We apologize for any confusion caused by the original figure labeling. In response, we have revised the figure labels and improved the overall clarity of the figures to minimize the possibility of misinterpretation. We thank the reviewer for bringing this issue to our attention, as these changes have strengthened the presentation of our results. We hope that, upon re-review, the reviewer agrees that the additional experiments introduced during the revision, while refining our mechanistic model, remain fully consistent with our original claims and further strengthen the study's conclusions.

1. *"They show that Segatella expansion occurs with arabinose and without E. coli."*
   This interpretation is not supported by our data and is contrary to the manuscript's central conclusion. Throughout the study, we consistently demonstrate that *E. coli*, and more broadly members of the Enterobacteriaceae, are indispensable for *Segatella* expansion within microbial communities (e.g., Figures 3A, 3B, and 4D). Even in experiments where arabinose was added, *E. coli* was required (Figure 3G). To address this misinterpretation and improve clarity, we split the original panel (see Figure R1 below) into two graphs showing the results without and with *E. coli*, respectively (see revised Figure 3G).

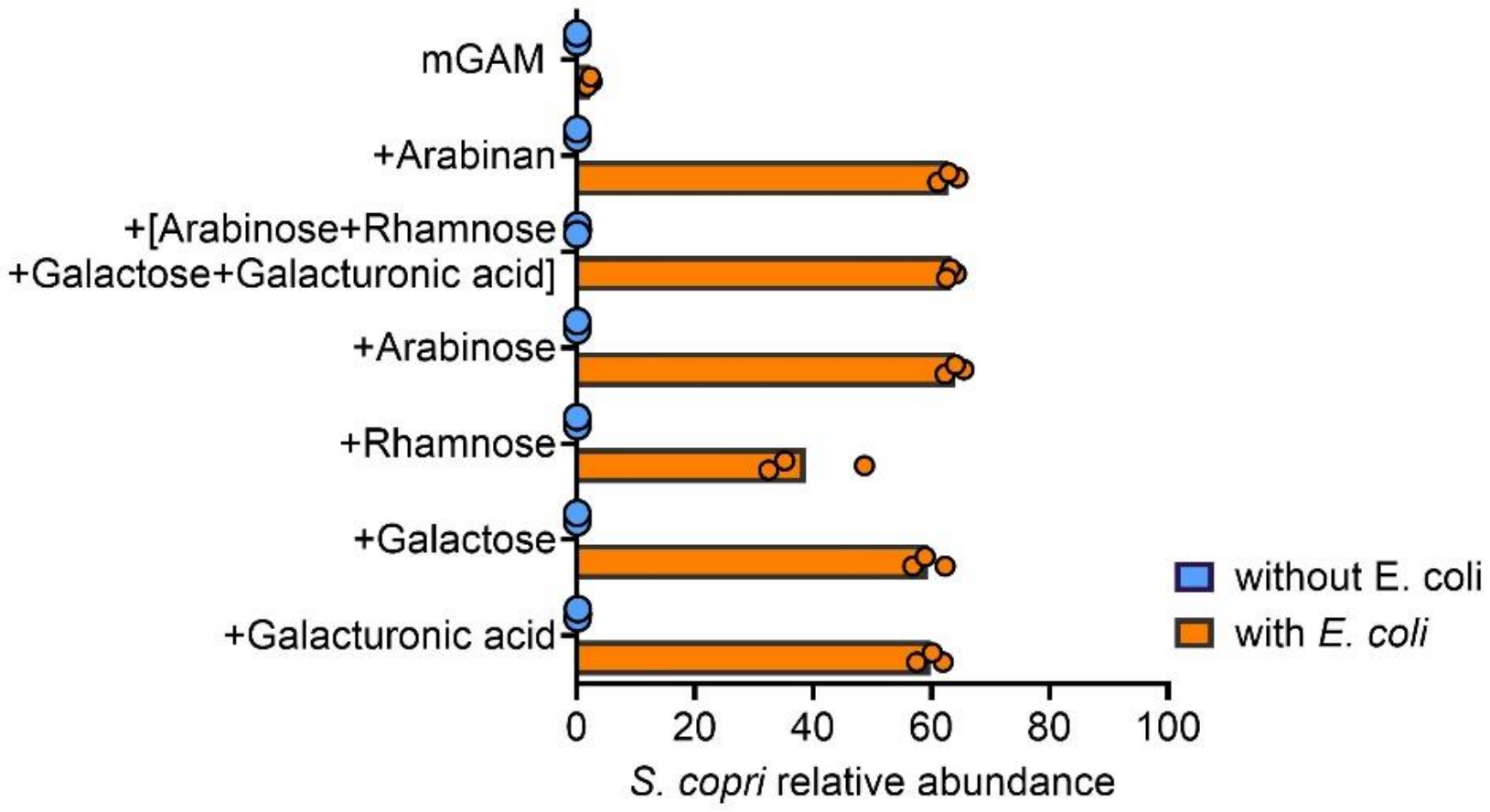

Figure R1. Competitions between *S. copri* and six Bacteroidaceae in the presence or absence of *E. coli* Nissle in mGAM supplemented with arabinan or the different arabinan-derived sugars. The relative abundance of *S. copri* in the communities is shown (n=3).

2. "*Their mouse experiments, Extended Data Fig. 5, were not done without Nissle.*"
No mouse experiments were performed in this study. Experiments that include *E. coli* Nissle are performed in synthetic communities. As shown above, Figure 3G includes the experiments presenting *S. copri* expansion in the presence or absence of *E. coli* Nissle (Figure R1 and Figure 3G). We used stabilized mouse-derived cecal communities ex vivo to show that *S. copri* requires *E. coli* for expansion within highly complex communities (as shown in Figure 5B, with the corresponding alpha diversity measures in Extended Data Fig. 5G). We have now revised these figures and added labels to clearly distinguish the different models used in each experiment (See amended Figure 3, Figure 5, and Extended data Figure 5).

3. "*Importantly, their knockout of the arabinose utilization genes also does not support the cross-feeding hypothesis. The authors appropriately changed their conclusions to "sugar-dependent signaling", but it's not clear what this mechanism is or its relevance.*
We propose that cross-feeding from *S. copri* to *E. coli* occurs, as our metatranscriptomic analysis shows that *E. coli* upregulates pathways involved in the utilization of simple sugars released by arabinan degradation (Figure 3E). We further demonstrate that *E. coli*-dependent *Segatella* expansion requires *S. copri* polysaccharide degradation by testing an *S. copri* knockout deficient in arabinan degradation (Figure 2C). Importantly, as noted, the knockout experiments in *E. coli* Nissle provided evidence that the release of sugars from polysaccharides, rather than their direct utilization, serves as a signal for *E. coli* to promote *S. copri* expansion (Figure 3H). As suggested by Reviewer 2, we revised the wording in the manuscript to "sugar-dependent signaling" to more precisely and appropriately reflect these findings. Several studies suggest that polysaccharide-derived sugars play roles beyond their nutritional value in the gut microbiota, acting as interspecies signals that modulate gene regulation and bacterial colonization potential (PMID: 30559205; PMID: 16735464; PMID: 39368472). Notably, while the exact molecular basis of this sugar-dependent signaling remains to be defined, identifying and experimentally narrowing the mechanism represents meaningful progress within the scope of the current manuscript. Moreover, we conclusively ruled out the possibility that classical cross-feeding, whereby one species provides nutrients that support the growth of another, is sufficient to explain the observed phenotype. This finding is particularly significant, as many current models of microbial interactions primarily rely on nutrient-based frameworks (PMID: 37054671). To better highlight the implications of our results, we have added a statement to the Discussion and included the relevant citations.

Line 452 "Polysaccharide-derived sugars have been shown to function beyond their nutritional role in gut bacteria, acting as interbacterial signaling molecules that directly influence gene expression and colonization capacity [46,57,58]."

Importantly, our work provides the first mechanistic framework explaining the microbial ecology and nutritional interactions that support *Segatella copri* colonization and expansion within gut microbial communities. Central to this advance was the development of robust experimental models that enable stable *S. copri* colonization. Using these systems, we provide the first mechanistic evidence of direct competition between Bacteroidaceae and *Segatella* and experimentally dissect the ecological interactions that promote *S. copri* expansion within gut microbial communities (Figure R2; Extended Data Figure 6D).

We acknowledge that our model presents certain limitations, such as reduced complexity, and may not fully recapitulate *in vivo* conditions. However, as requested by the reviewer, we validated our findings in highly complex human and mouse gut-derived communities and expanded our metagenomic analysis to include large human gut and food metagenomic datasets. Consequently, our study provides the first experimental evidence that refines existing hypotheses regarding the ecological and dietary factors influencing major gut microbiome signatures characterized by the predominance of Prevotellaceae or Bacteroidaceae.

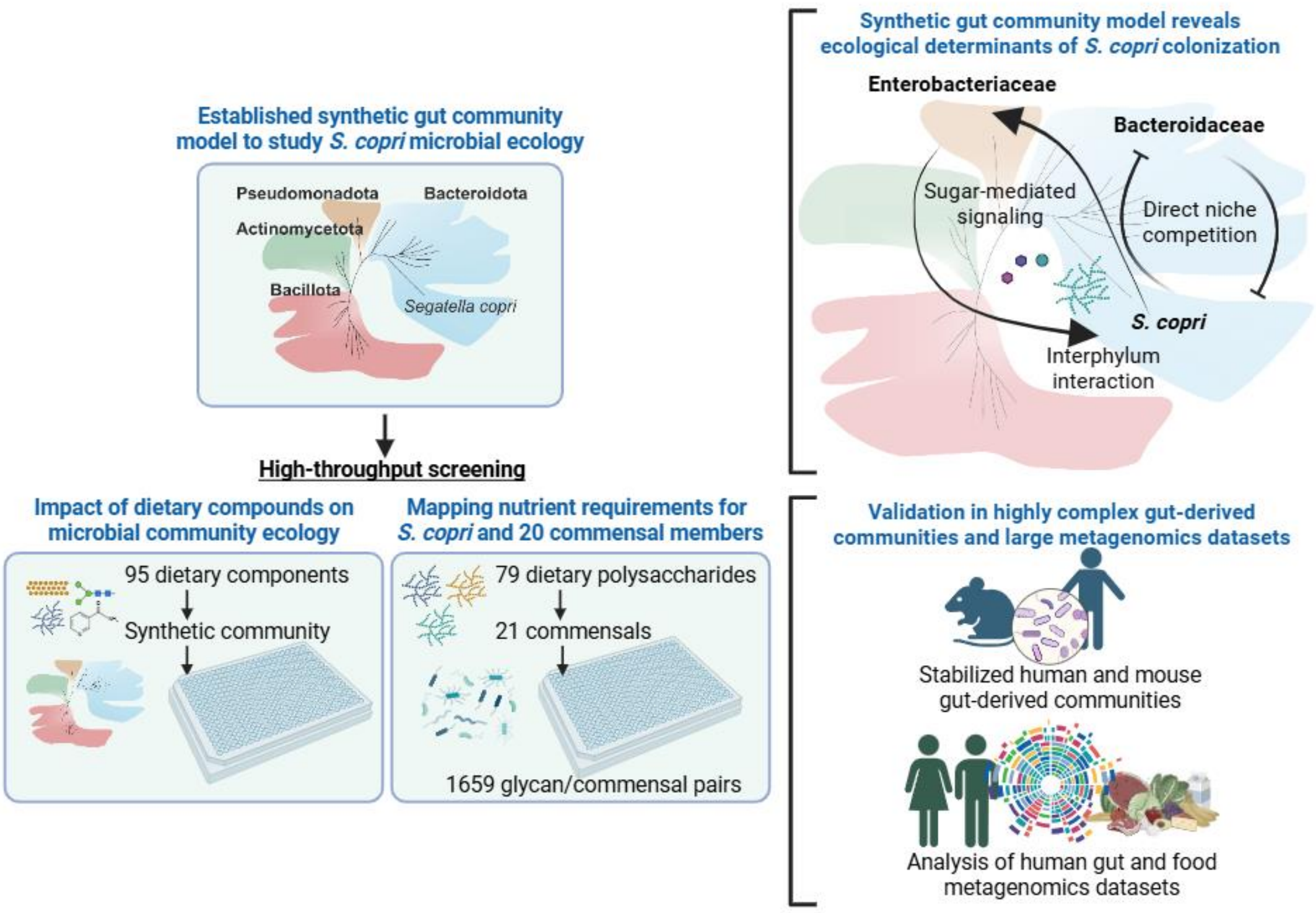

Figure R2. Schematic illustration of the experimental approaches and the main findings from this study. Specifically, we: (1) established a synthetic gut community that supports stable *S. copri* colonization; (2) systematically assessed the effects of a broad range of dietary glycans and vitamins on community ecology; (3) mapped the carbon nutrient requirements of *S. copri* and 20 commensal community members for anaerobic growth; (4) provided the first mechanistic evidence of direct competition between Bacteroidaceae and *Segatella*; (5) identified a beneficial interphylum interaction that promotes *S. copri* expansion through dietary components and is conserved among Enterobacteriaceae; (6) demonstrated, using representative *Segatella* isolates, that this interaction extends beyond *S. copri*; and (7) employed genetic approaches in both *S. copri* and *E. coli* to show that polysaccharide degradation is required for this interaction and that sugar-dependent signaling in *E. coli* drives its ecological relationship with *S. copri*.

Reviewer #3 (Remarks to the Author):

I thank the authors for their thorough responses to my previous comments, and for conducting follow-up experiments and revising the manuscript accordingly. I have no further comments or suggested edits on the revised version.

Authors' Response: We appreciate the reviewer's suggestions that improved the manuscript during the review process. We are particularly grateful for their explicit support of the revised version.

Response to reviewers' comments:

Reviewer #2:
Remarks to the Author:
Pardon my mistake about the synthetic community experiment. I jumped to the conclusion that those experiments had been done in mice given the implications of the work on colonization determinants, however colonization of mice with Segatella can be tricky.

The authors have addressed other comments.

Authors' response: We appreciate the reviewers' efforts and constructive comments during the review process. We are glad that the misunderstanding could be resolved.
